# Predicting the Reactivity of Acyclic Silylenes and Germylenes in Hydrogen Activation Using Machine Learning

**DOI:** 10.1002/advs.77947

**Published:** 2026-09-27

**Authors:** Michelle Kleinhaus, Henning Remm, Prasenjit Palui, Alexander Linke, Viktoria H. Gessner

**Affiliations:** ^1^ Chair of Inorganic Chemistry II Faculty of Chemistry and Biochemistry Ruhr‐University Bochum Bochum Germany

**Keywords:** bond activations, carbenes, machine learning, main group chemistry, silicon

## Abstract

Heavier carbenes can mimic transition metals in activating small molecules and strong bonds. Incorporating this ability into catalytic cycles demands careful, experimentally challenging tuning of their molecular properties via substituent variation. This study employs computational chemistry and data science to investigate dihydrogen activation by heavier carbenes to aid future candidate selection. Analyzing around 600 acyclic silylenes and germylenes derived from 40 different substituents, we found that a simple linear model predicts the activation barrier and reaction energy of H_2_ activation based solely on increments specific for the elements in the α‐position to the tetrel centre. We also established a multiple linear regression model, providing insights into quantitative structure‐reactivity relationships. While initially developed for H_2_ activation with silylenes, we demonstrate that this approach also is applicable to H_2_ activation with germylenes and H_2_O activation with silylenes, demonstrating its versatility in small molecule activation. The predicted activation energies were experimentally verified through kinetic studies, which confirmed the validity of our models and allowed us to identify candidates with promising substitution patterns. In doing so, we aim to inform the design of carbene‐like species capable of small‐molecule activation, thereby accelerating the development of main‐group catalysts.

## Graphical abstract

Statistical models for the prediction of the energy profile of H_2_ activation by silylenes and germylenes were developed. A simple model based on increments of the elements in α‐position enabled robust energy prediction, extrapolation to new structures, and transferability to new tetrylenes and substrates. The predictions were validated by experimental studies, enabling the identification of promising classes of heavier carbenes for H_2_ transfer.

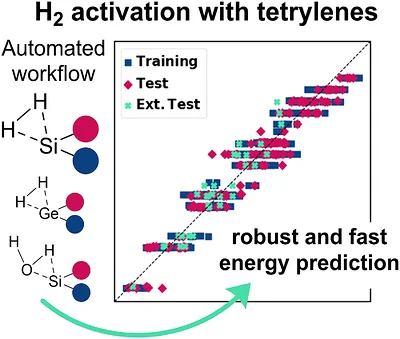

## 1 Introduction

**FIGURE 1:**
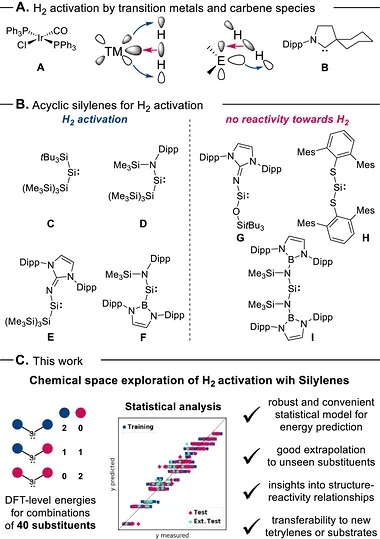
(A) Orbital schemes of H_2_ activation by transition metals and tetrylenes. (B) Reported acyclic silylenes for H_2_ activation (C) This work: Generation and exploration of the chemical space of H_2_ activation by tetrylenes using computational chemistry and data science.

Low‐valent main group compounds have long been considered as lab curiosities of limited stability and accessibility. However, research endeavors in the past three decades have changed this assumption and impressively demonstrated that species with most unusual bonding situations and electronic structures can be isolated and most importantly be applied in the activation of small molecules and strong bonds [1, 2, 3, 4]. This has sparked interest in applying such compounds in catalysis, with the prospect of developing sustainable, transition metal‐free alternatives to existing protocols. One of the most researched classes of low valent main group compounds applied in small molecule activation are carbenes and their heavier analogues, R‐E‐R’ (E = C‐Pb) [5]. This interest was particularly fueled by the landmark report by Bertrand and coworkers in 2007 showing that alkyl(amino)carbenes (e.g. **B**, Figure 1A) are able to activate dihydrogen at mild temperatures, a transformation long thought to be exclusive to precious transition metals such as palladium, rhodium, iridium or ruthenium (e.g. **A**, Figure 1A) [6]. This transition‐metal‐like behavior is enabled by the availability of an energetically high lying lone pair and a low‐lying vacant p‐orbital at the group 14 element, a feature reminiscent of the often partially filled d‐orbitals of the transition metals.

With the motivation to substitute precious metals in future hydrogenation reactions, vast research efforts have focused on the expansion of hydrogen activation to other carbenes as well as their heavier congeners. Investigations into the use of different substitution patterns and their impact on H_2_ activation led to a first understanding of the important electronic properties. For example, it has in general been accepted that a small HOMO‐LUMO and small singlet‐triplet energy gap enhance the activity of the divalent group 14 compounds toward bond activations [6, 7, 8, 9, 10, 11, 12]. Owing to the increased stability of the lone‐pair orbital within the group and the reduced back‐donation interaction between this orbital and the σ*‐orbital of H_2_ within the group, the activity decreases from carbon to lead [13, 14]. Thus, several acyclic silylenes [15, 16, 17, 18, 19, 20, 21, 22, 23, 24], but only few germylenes [10, 25, 26, 27, 28, 29] have been reported to undergo H_2_ activation at the group 14 element center (Figure 1B, Figure S3). However, the development of catalytic protocols requires consideration of not only the kinetic but also the thermodynamic profile of H_2_ activation to also enable efficient H_2_ transfer. To date, no carbene system has been identified that can both activate and transfer H_2_ in a manner comparable to that long established for transition metals. To bridge this gap, tetrylenes with carefully designed substitution patterns are required. However, the ability of silylenes and germylenes to activate H_2_ has proven difficult to predict and remains incompletely understood. Consequently, optimization of H_2_ activation by carbene species still relies on laborious trial‐and‐error approaches, in which substitution patterns are varied largely on the basis of chemical intuition.

Like the substitution pattern in tetrylene chemistry, the choice of ligands in transition metal complexes is decisive for their performance as homogeneous catalysts. To accelerate catalyst design, the quantification of steric and electronic ligand properties has early been addressed first by experimental [30, 31, 32, 33, 34, 35, 36] and later by computational methods [37]. Based on this featurization of ligands improved catalyst structures can nowadays be predicted using computational and machine learning methods, which has led to remarkable advances in the past years [38, 39, 40, 41, 42, 43, 44, 45, 46]. While this approach has been used in transition metal and organocatalysis, materials chemistry, and drug design [47, 48], including the prediction of reaction barriers for dihydrogen activation [49], a similar strategy has rarely been applied to the design of main group species [50, 51].

We envisioned that the field of main group element catalysis could benefit from such an approach and the computer‐aided selection and prediction of improved structures. To this end, we aimed at the computational generation and statistical exploration of a chemical space that allows to virtually describe and predict the propensity of tetrylenes to activate H_2_. Here, we demonstrate that carbene systems can indeed be modelled like transition metal complexes and can be described by similar features as used in catalyst design (Figure 1C). This strategy allowed us to predict the activation and reaction energy for H_2_ activation with high accuracy and robustness based on the elements in α‐position to the silylene, an approach that also can be transferred to other tetrylenes and substrates. In addition, multiple linear regression models with DFT‐level descriptors allow for a further understanding of the properties crucial for bond activations. Finally, we experimentally validated the predicted energies for two selected classes of germylenes, confirming our predictions and revealing promising substitution patterns for potential catalytic applications that should be prioritized in future studies.

## 2 Results and Discussion

### 2.1 Definition of the Chemical Space and Data Generation

**FIGURE 2:**
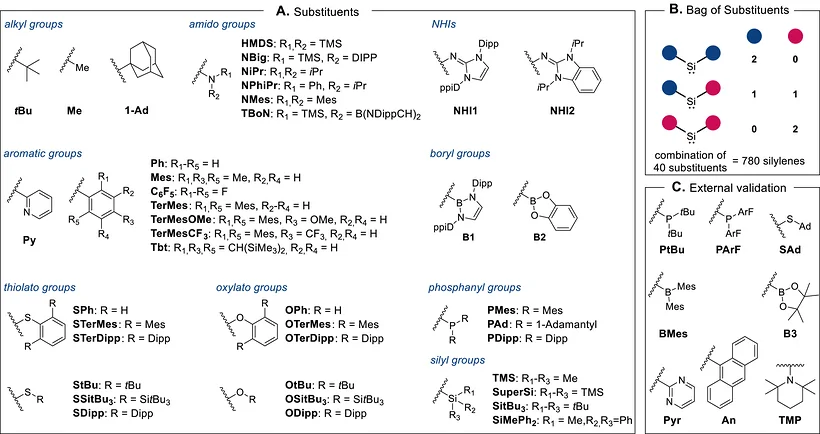
(A) Substituent space with 40 substituents containing amido, aryl, oxylato, thiolato, alkyl, phosphanyl, silyl, *N*‐heterocyclic imines and boryl substituents. (B) Combinatorial Bag of Substituents (BoS) approach to generate full chemical space of silylenes. (C) Additional eight substituents for the external test set for validation of the statistical models.

To explore the chemical space of heavier carbenes capable of hydrogen activation, we conducted a virtual screening of compounds of type RER' with E = Si and Ge. We decided to focus our studies on silylenes and germylenes which we expected to be the most promising candidates for reversible H_2_ activation due to the higher stability of the +2 oxidation state compared to carbenes combined with the higher activity and R‐E bond strengths relative to the tin and lead analogues [25, 26, 27, 28, 29]. Initially, we focused on silylenes, which we generated in silico by combinatorial enumeration of different R substituents bound to the silicon center. To generate a large but also meaningful chemical space, we chose substituents previously described in literature to form stable acyclic silylenes or germylenes, some of which were capable of activating dihydrogen (Figure 1B) [15, 16, 17, 18, 19, 20, 21, 22, 23, 24, 52, 53, 54, 55, 56, 57, 58, 59, 60, 61, 62, 63, 64, 65, 66, 67, 68], and enlarged the library by derivatives of these substituents. This selection was made to ensure synthetic accessibility of the calculated systems. Overall, 40 substituents of different sizes and with different donor atoms (Figure 2A) were used, including amido, alkyl and aryl, oxylato, thiolato, phosphanyl, silyl, *N‐*heterocyclic iminyl (NHI) and boryl groups. Next, the silylenes were virtually generated from combinations of these substituents and thus used in further studies (Figure 2B and Figure S1). An additional set of eight different substituents was designed for the generation of an external validation set which was held back during all chemical space explorations and statistical modelling and only used in the end to evaluate the generalizability of our models (Figure 2C, Figure S3, Table S1).

**FIGURE 3:**
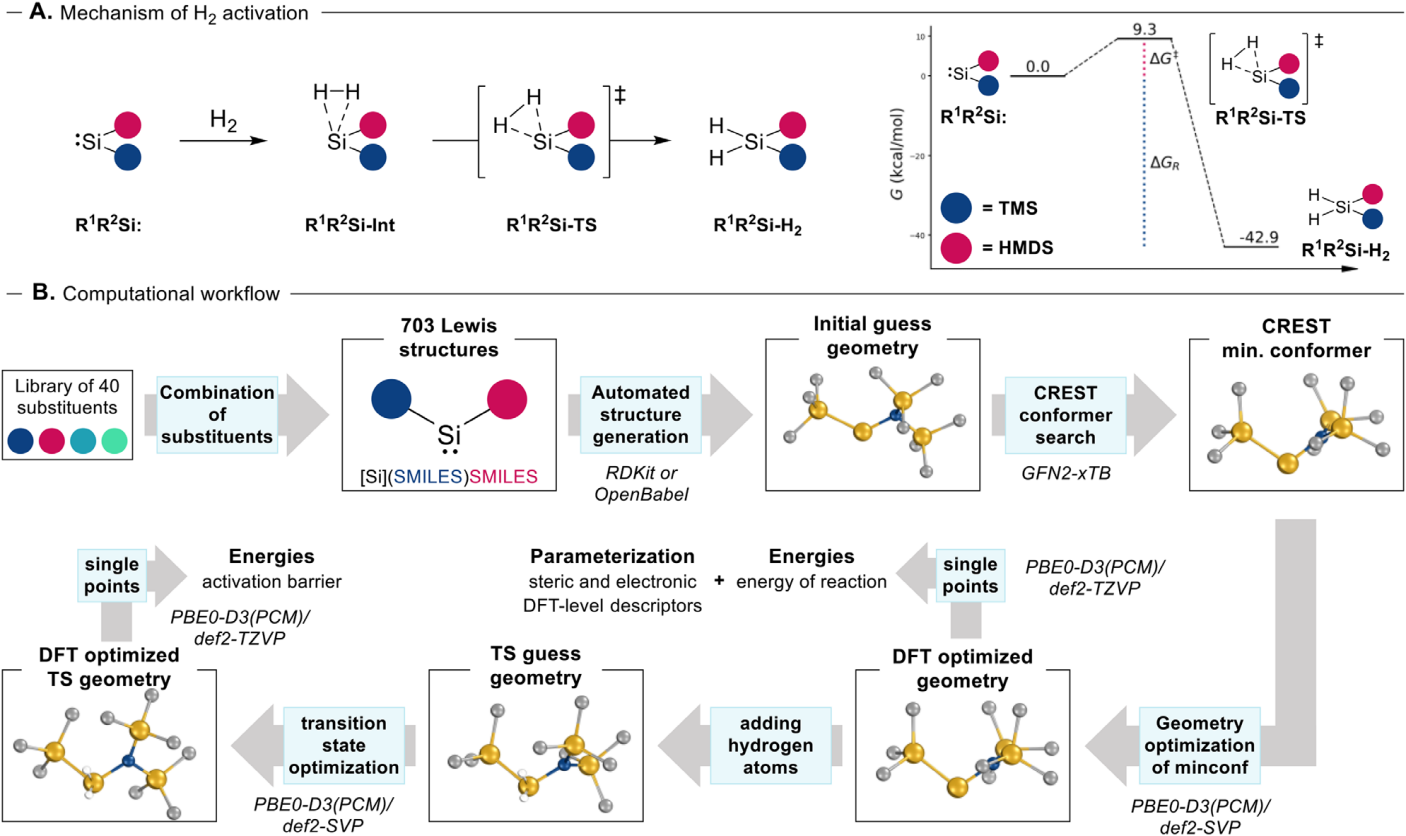
(A) Proposed mechanism for the H_2_ activation with silylenes used in the computational workflow with an example free energy profile for TMS‐Si‐HMDS. (B) Computational workflow to obtain the free energy profile and DFT‐level descriptors.

For each of the thus obtained 703 silylene structures the energy for transition state **R^1^R^2^Si**‐**TS**, product **R^1^R^2^Si**‐**H_2,_** and silylene **R^1^R^2^Si**: were calculated through an automated procedure (Figure 3B, see the Supporting Information for details). The initial geometries for the silylenes and the H_2_ activation products were generated from the corresponding SMILES strings via an initial geometry optimization at the GFN2‐xTB level of theory with the GBSA solvent model for THF [69, 70], followed by a subsequent conformer ensemble generation using CREST at the same level of theory (for CENSO check, Figure S4) [71, 72]. The lowest energy conformer was chosen for further optimization at the density functional theory (DFT) level PBE0‐D3(PCM)/def2‐TZVP//PBE0‐D3(PCM)/def2‐SVP [73, 74, 75, 76, 77, 78, 79]. The optimized ground states of the silylenes **R^1^R^2^Si**: were used to extract the electronic and steric properties (Table S2–S3, Figures S3–S8). For the transition states, the first structure guess was obtained from the structure of the silylene by addition of two hydrogen atoms to **R^1^R^2^Si**: using mean distances, angles, and dihedrals. It should be noted that during TS search two different modes of hydrogen activation were observed for some structures: the targeted single‐site activation at the silicon center and the cooperative H_2_ activation across the Si‐R linkage [80, 81, 82]. These cooperative H_2_ activations were mainly found for silylenes with boryl substituents due to the availability of an empty p‐orbital at boron, but also for some silyl substituents. Within this work we focused exclusively on single‐site H_2_ activations and therefore excluded those examples from the dataset. Furthermore, it must be noted that in some cases the value for the “barrier” (energy difference between **R^1^R^2^Si**‐**TS** and **R^1^R^2^Si**:) is negative. This can be explained by the formation of an intermediate **R^1^R^2^Si**‐**Int** (Figure 3A), in which H_2_ coordinates to the silicon center through an interaction of the σ‐bond of H_2_ and the vacant p‐orbital of the silicon center analogous to σ‐complexes in transition metal complexes. For highly reactive and instable silylenes, **R^1^R^2^Si**‐**Int** as well as **R^1^R^2^Si**‐**TS** are energetically favored compared to the free silylene and H_2_. In these cases, the energy difference is not equal to the actual barrier of the reaction but still reflects the impact of the substituent on H_2_ activation relative to the silylene. Thus, and for clarity, we used the term “barrier” in the following for all energy differences between the free silylene and the H_2_ activation transition state (see the Supporting Information for further details).

To challenge the transferability of our approach to other carbene‐like species and substrates, we applied our approach using the same workflow to H_2_ activation with germylenes and to H_2_O activation with silylenes.

### 2.2 Energy Increments for the Activation Energy

**FIGURE 4:**
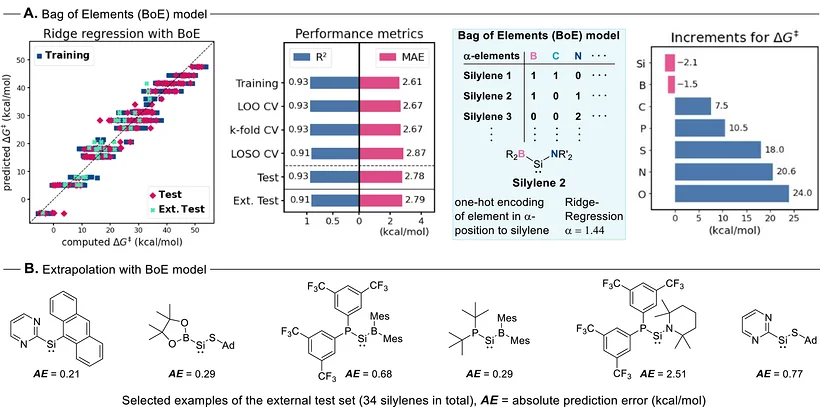
(A) Bag of Elements (BoE) model with a linear Ridge regression (α = 1.44) using one‐hot encoding of the element in α‐position to the silicon with a 70:30 random train‐test split. LOO CV: Leave‐One‐Out cross‐validation. k‐fold CV: 200 times repeated 4‐fold cross‐validation. LOSO CV: Leave‐One‐Substituent‐Out cross‐validation. Increments are calculated from the coefficients for every feature (element in α‐position) in the linear model. (B) Selected examples of the external test set demonstrating the strong ability of extrapolation of the BoE model toward two new and unseen substituents.

With the energy profiles of the H_2_ activation for 601 silylenes at hand, we sought to identify systematic patterns between the substituents and the resulting energies that would enable facile prediction of the activation potential of new silylenes. Principal component analysis of the approx. 100 DFT‐level descriptors (Figure S11) already indicated that steric properties have only a minor impact on the free energy profile. Instead, the activation is mainly determined by electronics effects, particularly by the nature of the elements in α‐position to the silicon center. Based on these observations and the Benson group theory in thermochemistry [83], we hypothesized that the calculated activation and reaction energies for the H_2_ activation can be expressed as a sum of contributions from each α‐element (Figure 4A). This “Bag of Elements” (BoE) approach mirrors Tolman's “Bag of Substituents”, used to describe the electronic properties of phosphines as the sum of substituent contributions [84, 85]. We would like to point out that although steric effects appear to have a minor influence on the free energy profile according to the dataset examined in this work, steric bulk plays a crucial role in stabilizing highly reactive species such as silylenes—most notably by preventing dimerization reactions. Moreover, steric effects may become more significant for larger substrates than dihydrogen, which can engage in additional dispersive or repulsive interactions with the substituents.

To construct the BoE model, we described all silylenes as a matrix using one‐hot encoding, with each matrix element expressing how often (0, 1 or 2) an element (B, C, N, O, Si, P, S) is present in the silylene. Linear Ridge regression using *L_2_* regularization with a random 70:30 train‐test‐split led to a highly predictive model for the activation barrier (Figure 4A, see Figure S9 for reaction energy), confirming the additivity of the element energy increments as reflected in the high quality of performance metrics. Next to standard cross‐validations (CV), such as Leave‐One‐Out (LOO) or k‐fold CV, we used Leave‐One‐Substituent‐Out cross‐validation (LOSO CV) as additional technique, where, analogously to the LOO CV, iteratively all data points for one substituent are excluded and predicted by the model trained on the remaining data points. Thus, the model's ability to extrapolate to new, unseen substituents is tested.

Satisfyingly, the BoE model proved to be robust across all CV techniques and could accurately predict the 34 silylenes in the external validation set. Simple addition of the energy increments of the two elements in α‐position to the silylene (listed in Figure 4) yields the dihydrogen activation barrier with an approximate error of only 2.8 kcal/mol. The element increments were found to be robust toward changes in the size and composition of the training dataset (Figures S12, S13 and S14, Table S4). Examples of new silylenes with the absolute error of the predicted activation barrier are shown in Figure 4B.

The reaction energy can similarly be predicted with an error of only 3.7 kcal/mol (see Figure S16). Thus, predictions in the fastest way thinkable can be made. While many statistical models rely on complex descriptors obtained from time‐consuming computational workflows and lack in their ability of extrapolation, this model can be applied without a computer at hand to any silylene with two of these elements in α‐position to silicon.

Inspired by a recent report advocating new machine learning approaches to identify promising candidates because of the data‐inefficiency of many machine learning models for activation barrier prediction [86], we investigated the minimum number of training data points required to build robust activation barrier models. To our delight, even different 5:95 random splits from various seeds with only 26 data points remaining in the training set yielded good and robust models with only slightly increase in the mean absolute error (MAE) values by ∼1 kcal/mol in the cross‐validations and external test set (Figure S15).

### 2.3 Structure‐Activity Relationships for H_2_ Activation

The BOE model demonstrated that the activation barrier can reliably be predicted by element energy increments. However, the available data set also allows for an analysis of relationships between these energy values and physico‐chemical properties of the silylenes and their substituents. To describe silylenes and their substituents and to guarantee interpretability of the results we selected a descriptor‐based parametrization of the molecules including steric and electronic properties (Tables S2+S3) [37, 38, 39, 40, 41, 42, 43].

**FIGURE 5:**
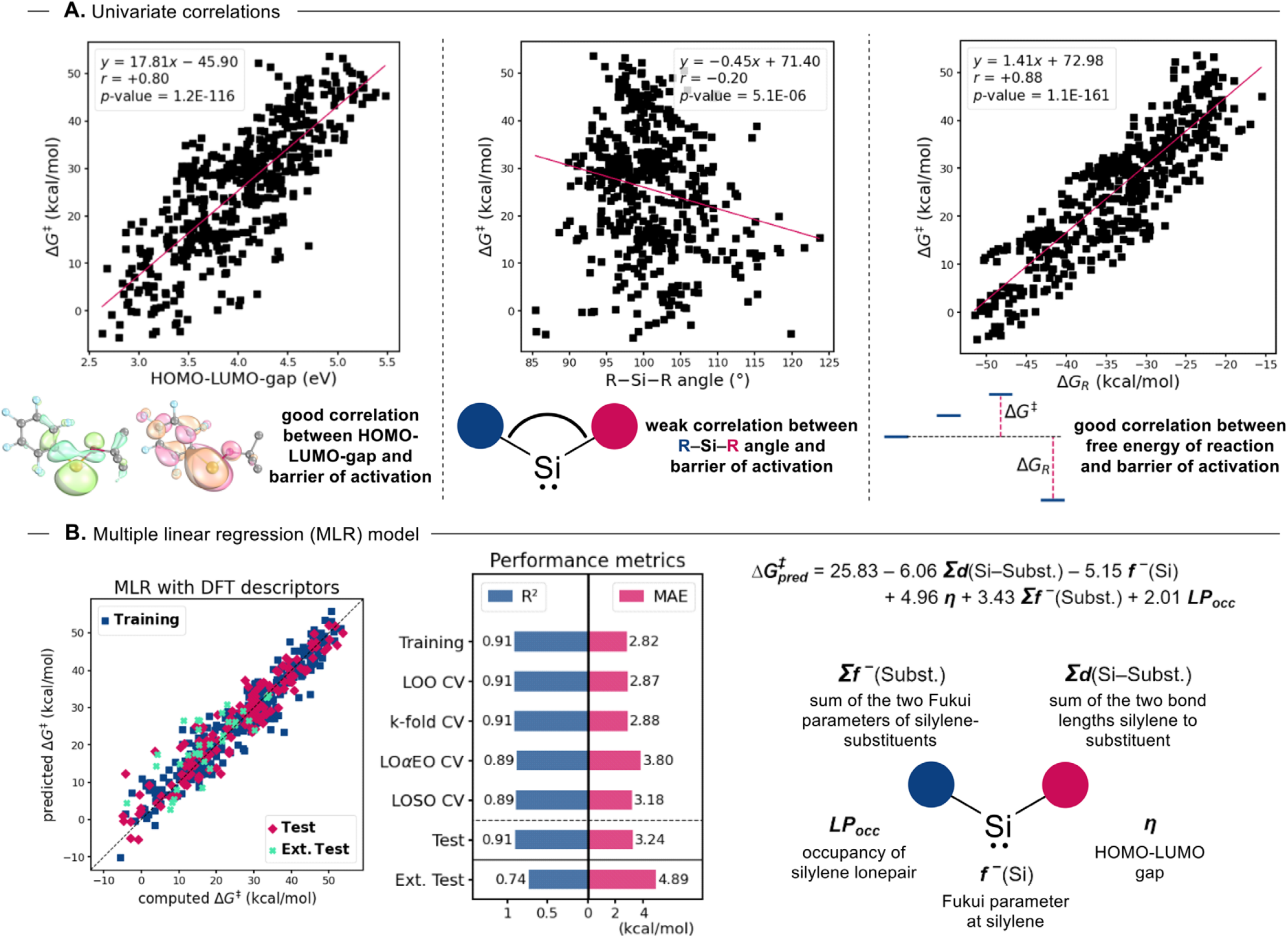
(A) Univariate linear correlations of descriptors with the barrier for H_2_ activation with silylenes. (B) Multiple linear regression (MLR) model on DFT‐level descriptors with a 70:30 random train‐test‐split. LOO CV: Leave‐One‐Out cross‐validation. k‐fold CV: 200 times repeated 4‐fold cross‐validation. LOαEO CV: Leave‐One‐α‐Element‐Out cross‐validation. LOSO CV: Leave‐One‐Substituent‐Out cross‐validation. Feature selection is based on a stepwise algorithm based on internal cross‐validation and linear independence of the features.

A total of 98 features were obtained from DFT calculations, population analyses, and conceptual DFT methods to characterize each silylene and the respective substituents (see the Supporting Information for details, Figures S5–S10). The HOMO‐LUMO gap is often used in literature to explain the reactivity of carbenes toward small molecule activation [7, 8, 9, 10, 11, 12, 14, 21, 22, 23, 24]. Indeed, this descriptor was found to strongly correlate (*r* = +0.80, Figure 5A) with the activation barrier in a linear fashion and is among the strongest correlating descriptors in our dataset. It confirms that small energy gaps decrease the activation barrier. Another frequently discussed feature is the R1‒Si‒R2 angle, which however exhibits only a weak linear correlation with the barrier according to our data (*r* = –0.20), with larger angles slightly decreasing the activation barrier. Interestingly, the best univariate linear correlation is achieved between the activation barrier and the overall reaction energy (*r* = +0.88), with higher barriers also leading to less stable silanes (vide infra for a more detailed discussion on implications for catalytic applications).

We also investigated non‐linear univariate correlations, which confirmed that the HOMO‐LUMO gap remains one of the best single descriptors for the energy barrier (Table S5). However, despite its strong correlation, using this feature alone in a statistical model was insufficient for reliable predictions of the activation energy (test set MAE = 6.1 kcal/mol). We therefore used multiple linear regression (MLR) to systematically enhance the model based on the HOMO‐LUMO gap with additional molecular features (see the SI for details on the feature selection; Figures S22 and S23).

A five parameter‐model was obtained that showed similar performance to the Bag of Elements model, but with weaker generalization ability, as seen in the performance on the external test‐set (Figure 5B). Next to the LOSO cross validation we also performed a Leave‐One‐α‐Element‐Out (LoαEO) cross validation, were all data points containing one element in α‐position systematically were excluded and predicted by the model trained on the remaining dataset. This CV quantifies the ability to extrapolate to new elements in α‐position. The MLR model proved robust under this cross‐validation, highlighting its extrapolative strength. However, the simple BoE model outperforms the MLR model, suggesting that the element increments encode more comprehensive chemical information than the five parameters in our MLR model.

The additional features in the MLR model include the sum of the two Si–Substituent bond lengths, the sum of the substituents Fukui parameters *f ^–^*, the Fukui parameter *f ^–^* at the silylene as well as the NBO derived occupancy of the silylene lone pair. It quickly becomes clear, that all these descriptors are exclusively electronic in nature and describe the electronic environment around the silylene center. This is in agreement with the findings from the chemical space analysis and the BoE model, confirming that steric effects can be neglected in determining the barrier of dihydrogen activation. From this finding and the BoE model's excellent performance, we conclude that silylene electronic properties are governed primarily by the α element, while the detailed structure of substituents beyond the α position has little impact on the activation barrier. This observation is fundamentally important for the design of tetrylenes for H_2_ activation, as the design of the substituents now can be focused on the synthetic accessibility, ease of practical handling, and suppression of possible side reactions such as dimerization of the tetrylenes.

### 2.4 Transferability of the Boe Approach

**FIGURE 6:**
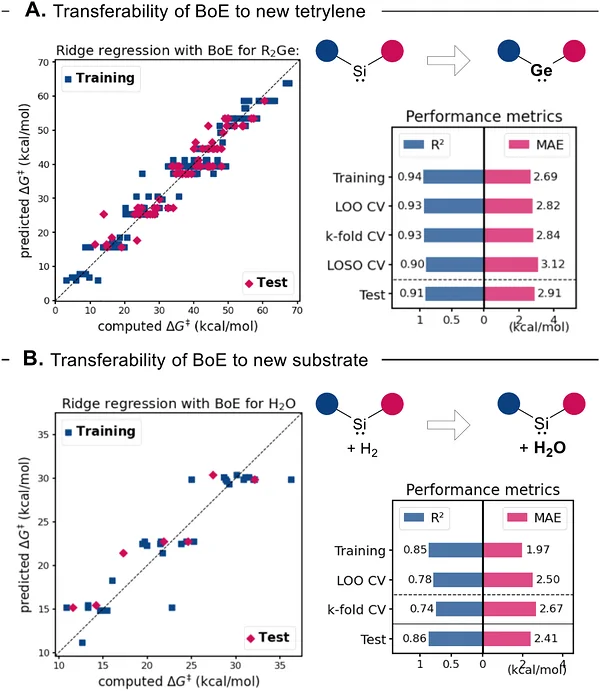
(A) Application of BoE model for activation barrier prediction of H_2_ activation with germylenes. (B) Application of BoE model for activation barrier prediction of H_2_O activation with silylenes.

Having established statistical models to rapidly predict the ability of silylenes to undergo H_2_ activation and understanding the underlying structure‐reactivity relationships, we next sought to transfer the BoE approach to heavier analogues. To this end, we used a smaller set of around 200 germylenes, substituted by the same 40 substituents user in the silylenes, to apply the BoE model for activation barrier prediction (Figure 6a, for reaction energy prediction see Figures S17+S18). Similar as for the silylenes, very good and robust performance metrics are obtained. Given the very strong linear correlation between the activation barriers of silylenes with those of the respective germylenes (*r* = +0.98, Figure S21) and the similar performance of the BoE model, we conclude that H_2_ activation by germylenes follows the same structure‐reactivity relationships as with silylenes. As expected, due to the increasing stability of the +2 oxidation state of germanium, a higher activation energy is in general required, and more stable products are formed. For an activation which requires 25 kcal/mol with a silylene – i.e which is just possible at room temperature – the activation barrier for the analogous germylene is around 35 kcal/mol and thus requires elevated temperature. In contrast, the reaction energies exhibit the opposite trend. Specifically, hydrogen activation by a germylene is approximately 25 kcal/mol less exergonic than the corresponding reaction involving a silylene.

Next to transferring the BoE approach to further carbene analogs, we tested its applicability on new substrates. To this end, we calculated H_2_O activation with silylenes and again were able to robustly predict the activation barrier (Figure 6b, for reaction energy prediction see Figures S19+S20). These results demonstrate the generality of the BoE approach and its transferability to predict small molecule activation with tetrylenes. However, the effectiveness of this approach may diminish as the size of the activated substrates increases, given the expected growing importance of steric effects.

### 2.5 Implications for Future Molecular Design

As described above, H_2_ activation by heavier carbenes shows a strong positive linear correlation between activation barrier and reaction energy, highlighting the challenge for catalytic applications: the barrier must be reduced to an accessible level without forming silanes that are too stable to transfer the activated hydrogen. For example, boryl and silyl groups generally lead to low activation barriers – in line with the silylenes hitherto known to activate H_2_ (Figure 1B) – but they form highly stable products, making H_2_ elimination or transfer challenging. On the other hand, N‐ and O‐substituents lead to the opposite trend: they yield less exergonic reaction profiles but impose high activation barriers.

**FIGURE 7:**
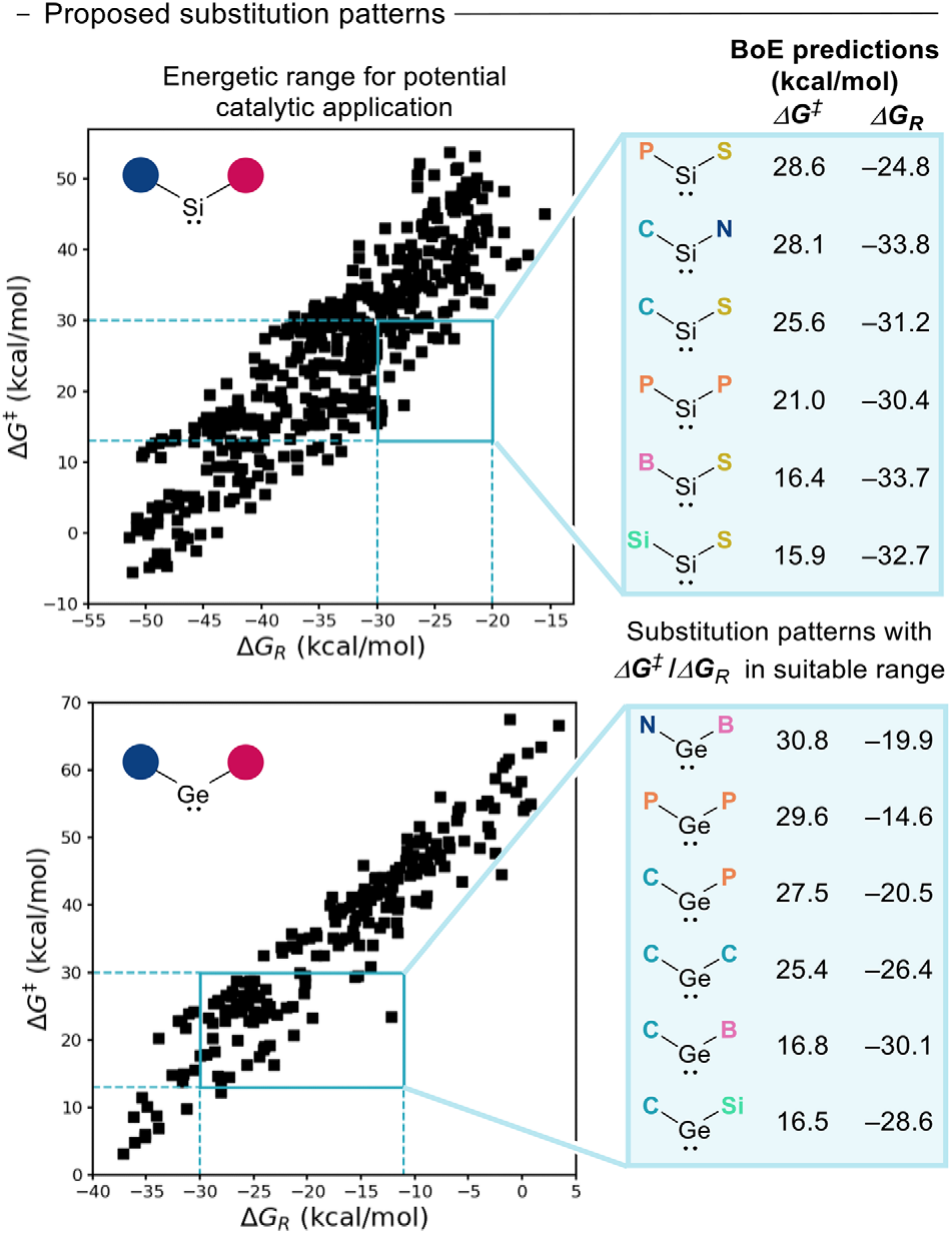
Selected interesting substitution patterns for potential catalytic applications of tetrylenes in small molecule activation based on energetic window of ±30 kcal/mol for the activation barrier and energy of reaction and the respective Bag of Elements predictions for both energies.

To identify promising candidates, we searched our virtual library of silylenes and germylenes based on the BoE predictions to select substitution patterns for potential catalytic applications (Figure 7). We selected a ± 30 kcal/mol range for the activation barrier and reaction energy as filtering criteria, defining an energetic window in which H_2_ activation under mild reaction conditions should, in principle, be possible. Interestingly, phosphido substituents – as studied experimentally by Izod and coworkers for germylenes [87, 88, 89, 90] – occupy this energetic window for both tetrylenes suggesting a so far untapped potential in small molecule activation. For silylenes, also thiolato‐substituents combined with other groups are potential candidates, while for germylenes alkyl‐ and aryl‐substituents seem to be most promising, considering their frequent presence in this energetic window and their respective BoE predictions. Nonetheless, a truly reversibly hydrogenation reaction will not be possible with these (or any other) substituents. However, this does of course not exclude hydrogenation reactions of unsaturated compounds such as alkenes, carbonyl compounds or imines, for which hydrogen transfer has a different activation barrier than the acceptor‐less dehydrogenation reaction studied here.

**FIGURE 8:**
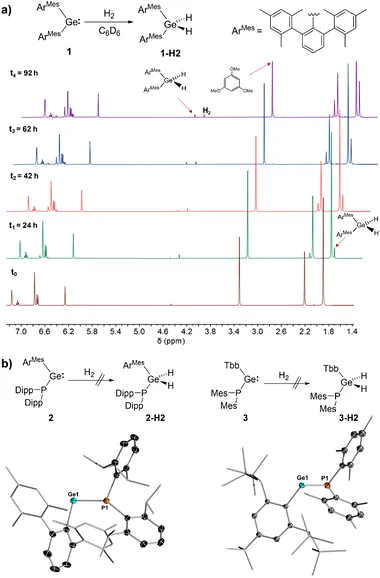
(a) Kinetic studies of H_2_ activation with diarylgermylene **1** at 298 K using ^1^H NMR spectroscopy and (b) (aryl)(phosphido)germylenes **2** and **3** investigated toward H_2_ activation together with their molecular structures. For details on the single crystal X‐ray diffraction analyses, see the Supporting Information (Table S11, Figures S41+S42). Selected bond lengths [Å] and angles [°]: **2**: Ge1‐P1 2.232(1), C*^ipso^* (Ar^Mes^)‐Ge1‐P1 102.3(1); **3**: Ge1‐P1 2.225(1), C*^ipso^* (Tbb)‐Ge1‐P1 106.9(1).

To validate our predictions and further narrow down suitable candidates for future studies, we sought to experimentally determine the limits for H_2_ activation with heavier carbenes. For these studies, we selected diarylgermylene **1**, as it has been reported to activate H_2_ at mild temperatures (60‐70 °C), and was therefore assumed to be suitable for experimental determination of the activation barrier through kinetic studies to cross‐validate the predicted energy barrier (Figure 8) [25]. To the best of our knowledge, no experimental values for activation barriers for H_2_ activation by silylenes and germylenes have been reported to date. Additionally, we chose two (aryl)(phosphido)germylenes, compounds **2** and **3**, which have not been previously investigated for H_2_ activation. However, these germylenes are predicted to activate H_2_ with an activation barrier of approx. 27.5 kcal/mol suggesting that this process may be accessible at elevated temperatures.

Initially, we probed the reactivity of diarylgermylene **1**, which was prepared according to the literature (see the Supporting Information for details) [91]. For H_2_ activation, a solution of **1** in benzene was exposed to 0.5 bar of H_2_ in a J‐Young NMR tube. NMR spectroscopic monitoring of the reaction process revealed that the activation was slow at 298 K, resulting in less than 50% conversion after 92 h (Figure 8a and Figure S32). For determining the activation energy, initial rates were measured by for reactions performed at 298, 303 and 313 K with 1,3,5‐trimethylbenzene as internal standard (see Figures S31–S40, Tables S6–S10). Eyring analysis yielded an activation barrier of 25.6 ± 0.6 kcal mol^−1^, perfectly aligning with our ML prediction (25.4 kcal/mol, Figure 8).

Next, we analyzed the propensity of (aryl)(phosphido)germylenes **2** and **3** for H_2_ activation. The two germylenes were prepared from the corresponding aryl‐germanium halides by reaction with the metal phosphide and could be isolated as an orange‐brown solid (**2**) and a maroon solid (**3**) in 59 and 70% yield, respectively (NMR analyses: Figures S24–S30). The structures of both germylenes are isostructural and were elucidated by single‐crystal X‐ray diffraction analysis (Figure 8b). In the solid state, each compound features a trigonal‐planar phosphorus center (Σ∡P = 360° (**2**), 359.6° (**3**)) connected to a bent, two‐coordinate germanium atom (∡Ge = 102.3(1)° (**2**), 106.9(1)° (**3**)) via short bonds, indicative of weak π‐interaction. Accordingly, the Ge–P bonds (2.232(1) Å (**2**) and 2.225(1) Å (**3**)) are considerably shorter than those reported for aryl‐phosphido germylenes and the cyclic amino(phosphido)germylene, where the phosphorus centers adopt a pyramidal geometry [10, 90, 92]. However, the structural metrics resemble those observed in the few diphosphido germylenes bearing mixed planar and pyramidal phosphorus environments, further supporting Ge–P π‐conjugation [87, 93].

The activity of germylenes **2** and **3** toward H_2_ activation was tested using the same protocol as described above. As suggested by the already slow progress of the reaction with **1**, no H_2_ activation was observed for these germylenes at room temperature, whereas an unselective decomposition of germylenes occurred upon heating to 100 °C. This observation is consistent with the higher barrier predicted for these systems, further narrowing down suitable substitution patterns for small molecule activation and transfer.

Overall, the ML predictions and experimental validations suggest that dialkyl‐ and diarylgermylenes as well diphosphidosilylene and (alkyl/aryl)(thiolato)silylenes are the most promising classes of heavier carbenes to realize H_2_ activation and transfer at mild conditions. Future studies should focus on these substitution patterns to maximize the stability of low‐valent main‐group species against side reactions (e.g., dimerization and intramolecular C–H activation), while preserving an accessible site for H_2_ binding and transfer to other substrates.

## 3 Conclusion

In summary, we have developed a computational workflow to statistically explore the chemical space of dihydrogen activation at the group 14 element centers in silylenes and germylenes. By analyzing reaction energies and barriers for silylenes with 40 different substituents, we discovered that energy increments, solely based on the elements in the α‐position to the silicon center, enable rapid and accurate prediction of activation barriers and reaction energies. This finding provides a straightforward method for estimating free energy profiles, which facilitates extrapolation to new substituent structures and transferability to other tetrylenes and small substrates. Our results, supported by a multiple linear regression model using DFT‐level descriptors, indicate that dihydrogen activation is predominantly influenced by electronic properties at the silicon center. Although steric effects and substituent structures beyond the α‐position are important for stabilizing these reactive species, they play a less significant role in the energy profile of H_2_ activation. This influence may, however, increase for larger substrates than dihydrogen, which can engage in additional dispersive or repulsive interactions.

Experimental cross‐validation confirmed the validity of our predicted energies and enabled – based on our virtual library – the identification of promising substitution patterns of tetrylenes for potential catalytic applications. These insights are expected to significantly influence future design strategies of tetrylenes, but also other neutral and charged carbene‐like species, for dihydrogen activation [94].

### 3.1 Computational Details

Details about the experimental details, density functional theory calculations and statistical modelling are given in the Supporting Information. A Jupyter notebook containing all presented statistical models and metrics as well as all relevant data and optimized geometries are provided in an online repository under https://radar4chem.radar‐service.eu/radar/en/dataset/BLZftsSEkmleGhbh?token = hpPEaEBWgkhlEkCwRpaO.

## Author Contributions

**Viktoria H. Gessner**: conceptualization, investigation, validation, visualization, formal analysis, supervision, resources, Writing – review and editing, funding acquisition. **Michelle Kleinhaus**: investigation, validation, methodology, formal analysis, data curation, visualization, writing – original draft, software. **Henning Remm**: writing – original draft, investigation, visualization, software, formal analysis, data curation, validation. **Prasenjit Palui**: writing – original draft, investigation, visualization, formal analysis. **Alexander Linke**: investigation.

## Conflicts of Interest

The authors declare no conflicts of interest.

## Supporting information




**Supporting File**: advs77947‐sup‐0001‐SuppMat.pdf.

## Data Availability

The data that support the findings of this study are openly available in RADAR4Chem at https://radar4chem.radar‐service.eu/radar/en/dataset/BLZftsSEkmleGhbh?token=hpPEaEBWgkhlEkCwRpaO.
