# Supplementary material for: Predicting the Reactivity of Acyclic Silylenes and Germylenes in Hydrogen Activation Using Machine Learning
Source: Adv Sci (Weinh). 2026 Sep 27:e77947. Online ahead of print. doi: 10.1002/advs.77947 (PMC13616320; doi:10.1002/advs.77947)
Supplement: Supplementary file 1 [file ADVS-9999-e77947-s001.pdf]

Predicting the Reactivity of Acyclic Silylenes and Germylenes in Hydrogen Activation Using Machine Learning

Michelle Kleinhaus,[a,+] Henning Remm,[a,+] Prasenjit Palui[a,+] Alexander Linke[a] and Viktoria H. Gessner[a]*

[a] Inorganic Chemistry II, Faculty of Chemistry and Biochemistry, Ruhr-University Bochum Universitätsstraße 150, 44801 Bochum, Germany; E-mail: viktoria.gessner@rub.de

[+] These authors contributed equally to this work.

Table of contents

1. Computational workflow 2

1.1. Calculations of the activation energies and reaction enthalpies 2

1.2. Chemical space 5

1.3. Conformer cross-checks with CENSO 7

2. DFT-level descriptors 8

2.1. Global features 8

2.2. Substituent specific features 10

2.3. Definition of steric features 11

2.4 Definition of electronic features 15

3 Data Science 17

3.1. Principal component analysis (PCA) 17

3.2 Bag of Elements: $\Delta G^{‡}$ for silylenes 18

3.3 Bag of Elements: ΔG_R for silylenes 24

3.4 Bag of Elements: ΔG_R for germylenes and increments for $\Delta G^{‡}$ 24

3.5 Bag of Elements: ΔG_R for H_2O activation with silylenes and increments for $\Delta G^{‡}$ 25

3.6 Linear correlation of energies of silylenes and germylenes 26

3.7 Linear and Non-linear univariate correlations 27

3.8 Multiple linear regression: Feature selection 28

4 Experimental Studies 30

4.1 General information 30

4.2 Synthesis of germylenes 31

4.3 H_2 activation with diarylgermylene 1 37

5 Crystal structure determination 44

5.1 General Information 44

5.2 Details on the structure solutions 44

5.3 Crystal structure analysis of 2 45

5.4 Crystal structure analysis of 3 46

6 References 47

Data availability: Details about the density functional theory calculations and statistical modelling are given below. A Jupyter notebook containing all presented statistical models and metrics as well as all relevant data and optimized geometries are provided in a RADAR4Chem folder under https://radar4chem.radar-service.eu/radar/en/dataset/BLZftsSEkmleGhbh?token=hpPEaEBWgkhlEkCwRpaO .

1. Computational workflow

1.1. Calculations of the activation energies and reaction enthalpies

The computational workflow was used for the automated treatment of all silylenes and germylenes and is discussed for the case of silylenes. For each system, we attempted to obtain an optimized geometry and energy on DFT level for the silylene **R^1R^2Si:**, the silane product **R^1R^2Si-H_2** and the transition state **R^1R^2Si-TS** for the single-site H_2 activation at the silicon center.

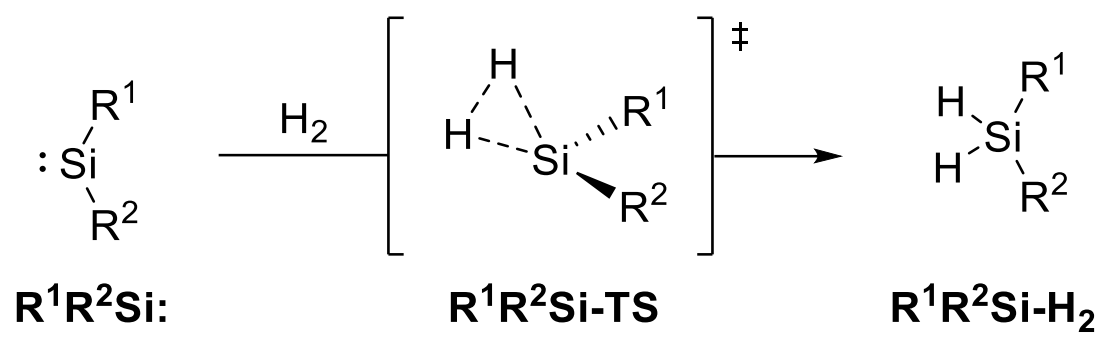

Scheme S1. Single-site H_2 activation at silicon center of silylene **R^1R^2Si:**.

Ground states

Initial geometries guesses for silylenes and silanes were obtained from RDKit[1] or Open Babel[2] based on SMILES strings. The SMILES strings for silylenes and silanes were generated through combination of substituents (Table S1) as follows:

- silylene: [Si](SMILES **substituent1**)SMILES **substituent2**
- silane: [SiH2](SMILES **substituent1**)SMILES **substituent2**

These initial geometries were optimized at the GFN2-xTB level of theory[3], followed by a subsequent conformer ensemble generation using CREST (version 2.11) at the GFN2-xTB level with the GBSA solvent model and the solvent parameters of THF (xTB version 6.4.1).[4] From the generated conformer ensemble, the conformer with the lowest energy was chosen for further optimization on DFT-level, respectively. All DFT calculations were performed using Gaussian (version 16 C.01).[5] The geometry optimizations, subsequent frequency and single point calculations were performed at the PBE0-D3(PCM)/def2-TZVP//PBE0-D3(PCM)/def2-SVP level of theory THF as solvent.[6, 7, 8, 9,10] For each silylene an NBO analysis and additional single point calculations were performed for the radical cation and radical anion on the optimized geometry for the silylene. NBO analysis was performed using the NBO program (version 7.0).[11]

Transition states

The optimized geometries of the silylenes were used for a generation of initial guess geometries of the transition states. Data for the optimal position of the hydrogen atoms was obtained from manual transition state searches using relaxed surface scans. As in the TS the hydrogen atoms can be located at two sides of the silylene, two initial structures were generated, respectively. A constrained geometry optimization was performed first with the following parameters (B(OND): H-Si, A(NGLE): H-Si-L1, D(IHEDRAL): H-Si-L1-L2):

a. **For H1**: B1 = 1.55 Å, A1 = 109.6°, D1 = (-)127.4°
b. **For H2**: B2 = 1.69 Å, A2 = 91.0°, D2 = (-)92.5°

L1 and L2 are the two substituents to the silylene. After the first constrained geometry optimization an unconstrained optimization the transition state was performed. The transition states were confirmed by the presence of the right vibrational mode with one imaginary frequency. In some cases, the convergence of the transition state, silylene or activation product was not successful so that some datapoints are missing. In addition, two different modes of hydrogen activation were observed for some structures: the targeted single-site activation at the silicon center and the cooperative H_2 activation across the Si-R linkage. These cooperative H_2 activations were mainly found for silylenes with boryl substituents due to the availability of an empty p-orbital at boron, but also for some silyl substituents. Within this work we focused exclusively on single-site H_2 activations and therefore excluded those examples from the dataset.

Negative barriers. As stated in the manuscript, some silylenes showed a negative “barrier” (energy difference between **R^1R^2Si-TS** and **R^1R^2Si:**). This comprises 16 silylenes with silyl and/or boryl group, i.e. those which are expected to be most reactive. This observation can presumably be explained by the formation of an intermediate **R^1R^2Si-Int** (Figure 3A), in which H_2 coordinates to the silicon center through an interaction of the σ-bond of H_2 and the vacant p-orbital of the silicon center analogous to σ-complexes in transition metal complexes. For highly reactive silylenes, both **R^1R^2Si-Int** as well as **R^1R^2Si-TS** are energetically favored compared to the free silylene and H_2. In these cases, the energy difference is not equal to the actual barrier of the reaction but still reflects the impact of the substituent on H_2 activation relative to the silylene. Therefore, and for clarity, the term “barrier” was used for all energy differences between the free silylene and the H_2 activation transition state.

In order to verify the formation of a silylene H_2 adduct for these systems, we exemplarily calculated the energy profile for the silylene with a C_6F_6 and a TMS group. As shown in Figure S1, a stable silylene-H_2 complex could be located on the energy surface, which is only slightly more stable than the silylene and free H_2 (-0.1 kcal/mol). This complex activates H_2 via a transition state that remains slightly lower in energy. In such cases, we consider the H_2 activation to be essentially barrierless. To assess how the model would respond to setting all negative barriers to zero, we performed the ridge regression (see Chapter 3) with all negative barriers replaced by zero. The results, shown in Figure S2, indicate that the impact of these 16 values on the model is minimal. However, since these values arise from the consistent

energy definition used throughout the dataset and retain information on the relative stabilization of the H_2-activation pathway, we retained them in the regression analysis.

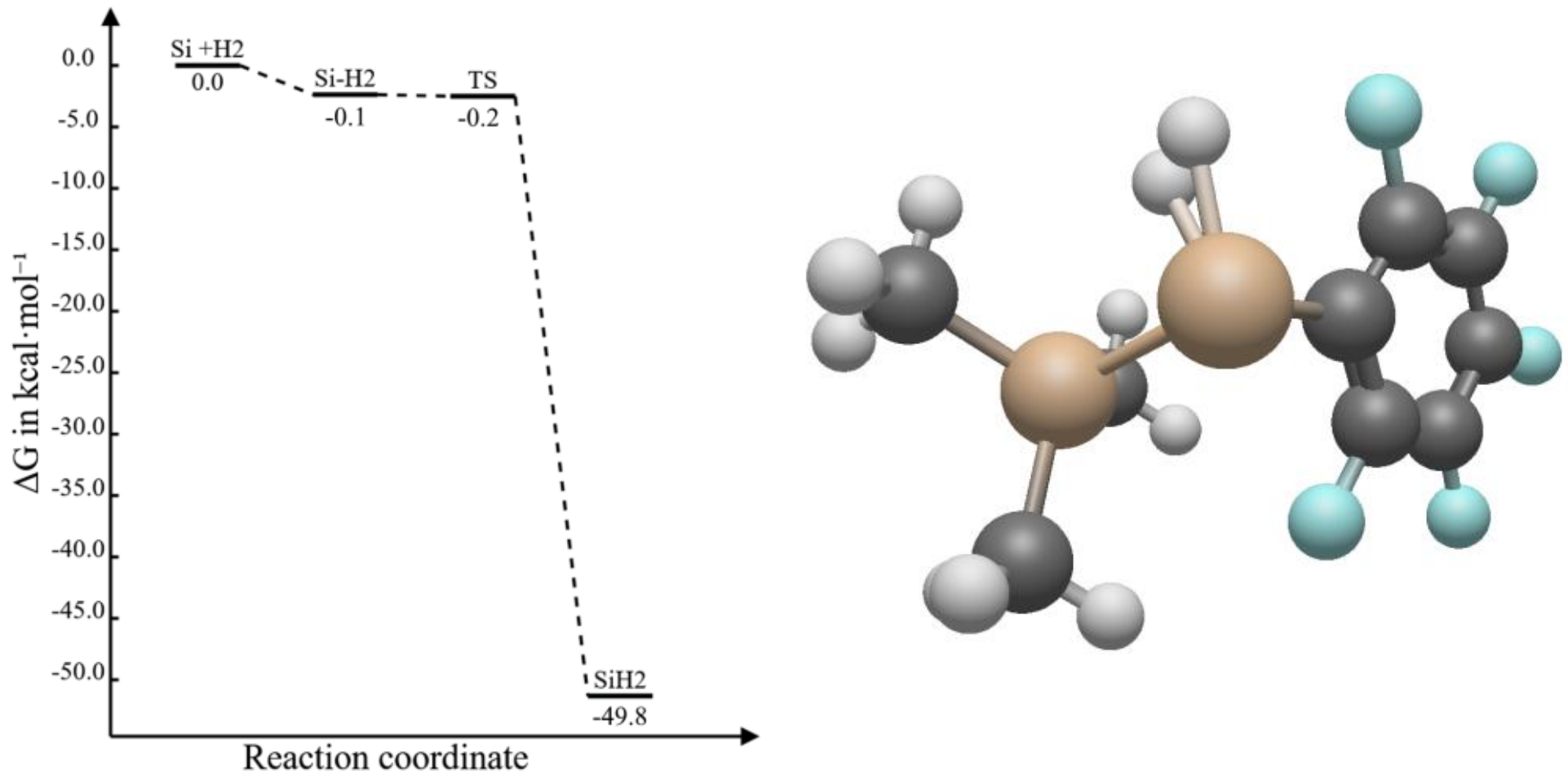

Figure S1. (left) Reaction profile of the H_2 activation with $(C_6F_5)Si(SiMe_3)$ and (right) H_2 complex of the silylene preceding H_2 activation (PBE0-D3(PCM)/def2-TZVP//PBE0-D3(PCM)/def2-SVP).

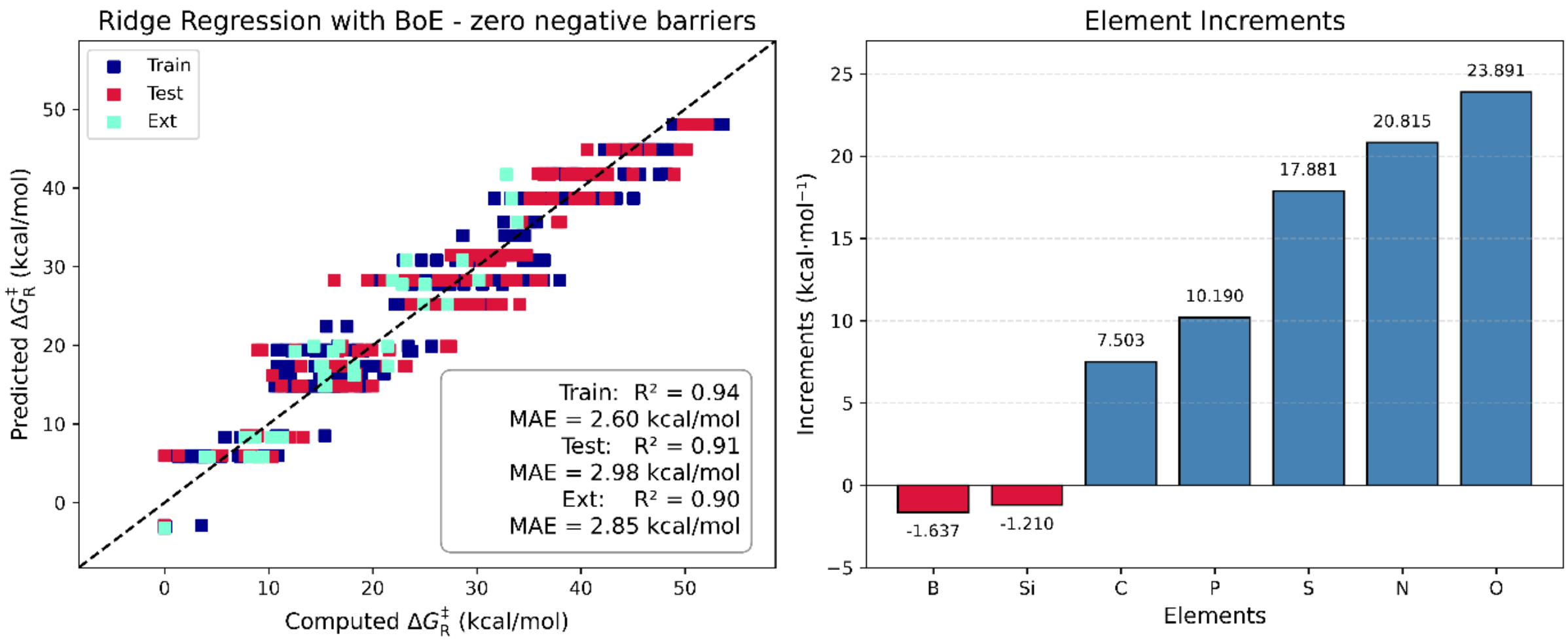

Figure S2. Bag of Elements (BoE) model with a linear Ridge regression (α = 1.44) for the activation barrier on the one-hot encoding of the element in α-position to the silicon with a 70:30 train-test split (random_state = 42) and all negative barriers set to zero.

Dataset composition.

Our dataset comprises 40 unique substituents, plus an additional eight substituents that belong to the external validation set, and it was assembled in several stages. Initially the data set contained only 23 substituents (listed as dataset 1 in Table S1); all possible pairings of these substituents were generated, giving a total of 276 silylenes. Subsequently, 17 more substituents were introduced (dataset 2 in Table S1); for these we considered only mixed combinations with the original 23 substituents, which yields 23 × 17 = 391 additional silylenes. Finally, eight extra substituents were added for the external validation set, for which each all combinations (of the eight new substituents) were calculated, resulting in 36 silylenes. In total,

276 + 391 + 36 = 703 distinct silylene structures were prepared as the initial input for the subsequent DFT calculations.

During the transition-state search, some of these systems underwent cooperative H_2 activation across the Si–R linkage instead of the intended single-site activation at the silicon center. These cooperative H_2 activations were observed mainly for silylenes with boryl substituents, due to the availability of an empty p orbital at boron, but also for some silyl substituents. In this work, we focused exclusively on single-site H_2 activation. Therefore, these systems were excluded from the dataset. In some cases, convergence to the transition state, silylene, or activation product was not achieved, so additional data points are missing. Therefore, of the 703 possible systems, only 601 are reported; among these, 34 belong to the external validation set.

The results for these 601 silylenes are listed in the CSV file *energies_tetrylenes* provided in the online repository (see link above). The test-training set is denoted as "initial", the external validation set as "ext. validation". Table S1 lists the frequency of each substituent in the dataset.

1.2. Chemical space

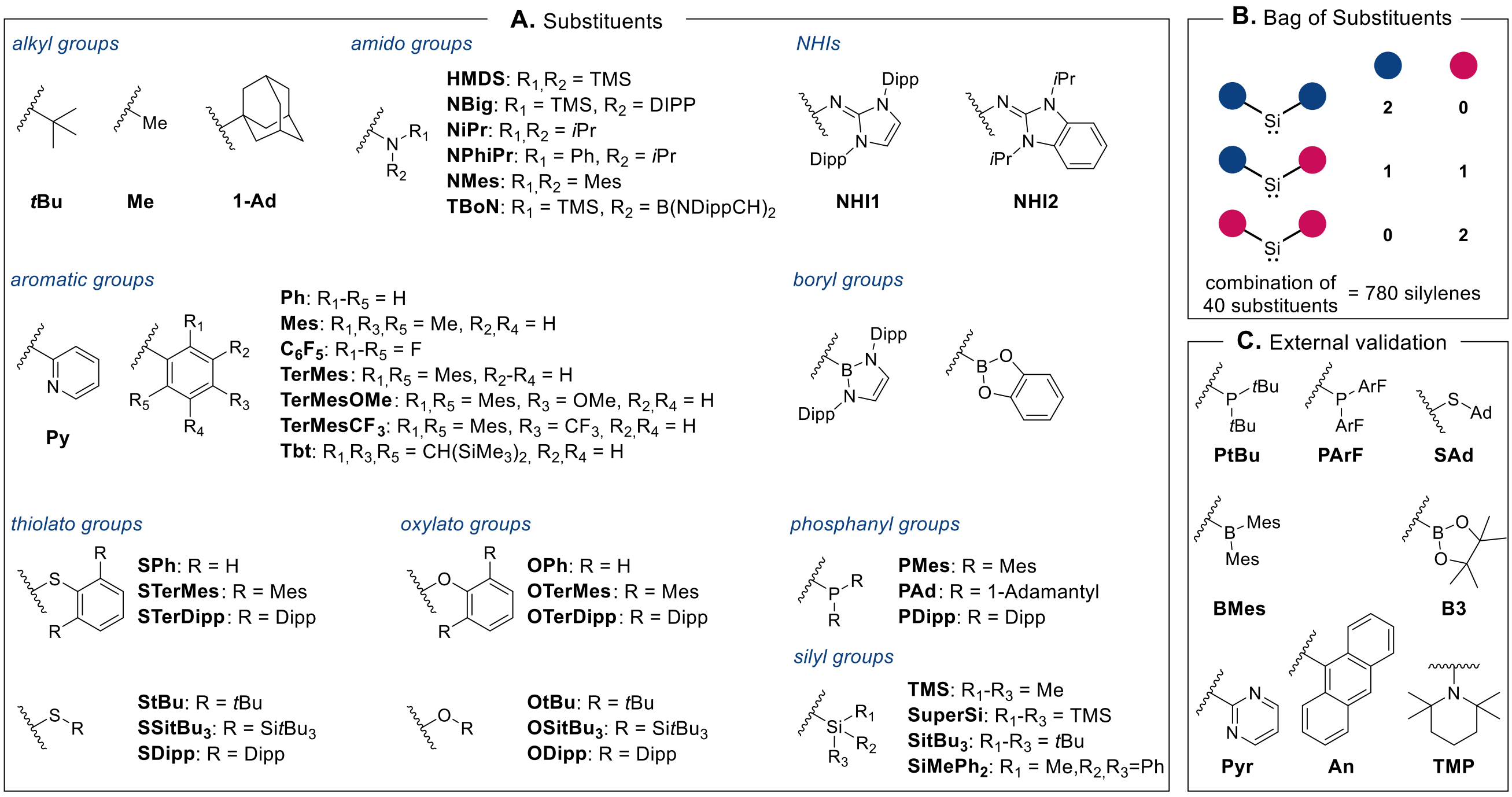

Figure S3. Scope of substituents containing amido, aryl, oxylato, thiolato, alkyl, phosphanyl, silyl, NHI and boryl substituents.

Table S1. SMILES string for each substituent used to generate the tetrylenes.

Substituent	SMILES	Counts in dataset
**Dataset 1**		
**HMDS**	N([Si](C)(C)C)[Si](C)(C)C	38
**NBig**	N([Si](C)(C)C)c1c(cccc1C(C)C)C(C)C	39
**NiPr**	N(C(C)C)C(C)C	39
**TBoN**	N(b1n(ccn1c1c(cccc1C(C)C)C(C)C)c1c(cccc1C(C)C)C(C)C)[Si](C)(C)C	33
**Ph**	c1ccccc1	41
**Mes**	c1c(cc(cc1C)C)C	39
**Py**	c1ccccn1	41
**C6F5**	c1c(c(c(c(c1F)F)F)F)F	41
**TerMes**	c1c(c2c(C)cc(C)cc2C)cccc1c1c(C)cc(C)cc1C	34
**TerMesOMe**	c1c(c2c(C)cc(cc2C)C)cc(cc1c1c(C)cc(cc1C)C)OC	36
**TerMesCF3**	c1c(c2c(C)cc(cc2C)C)cc(cc1c1c(C)cc(cc1C)C)C(F)(F)F	37
**OPh**	Oc1ccccc1	37
**OTerMes**	Oc1c(cccc1c1c(cc(cc1C)C)C)c1c(cc(cc1C)C)C	30
**SPh**	Sc1ccccc1	39
**STerMes**	Sc1c(cccc1c1c(cc(cc1C)C)C)c1c(cc(cc1C)C)C	33
**tBu**	C(C)(C)C	41
**Me**	C	41
**TMS**	[Si](C)(C)C	32
**SuperSi**	[Si]([Si](C)(C)C)([Si](C)(C)C)[Si](C)(C)C	33
**NHI1**	[N][C]1N(C=CN1c1c(cccc1C(C)C)C(C)C)c1c(cccc1C(C)C)C(C)C	30
**NHI2**	[N][C]1N(c2ccccc2N1C(C)C)C(C)C	34
**B1**	b1n(ccn1c1c(cccc1C(C)C)C(C)C)c1c(cccc1C(C)C)C(C)C	30
**B2**	b1oc2c(cccc2)o1	30
**Dataset 2**		
**NPhiPr**	N(C(C)C)c1ccccc1	21
**NMes**	N(c1c(C)cc(C)cc1C)c2c(C)cc(C)cc2C	18
**Tbt**	c1c(C([Si](C)(C)C)[Si](C)(C)C)cc(C([Si](C)(C)C)[Si](C)(C)C)cc1C([Si](C)(C)C)[Si](C)(C)C	12
**1-Ad**	C1(C2)C[C@H](C3)C[C@@H]2C[C@H]3C1	21
**SitBu3**	[Si](C(C)(C)C)(C(C)(C)C)C(C)(C)C	11
**SiMePh2**	[Si](C)(c1ccccc1)c2ccccc2	15
**OtBu**	OC(C)(C)C	18
**OSitBu3**	O[Si](C(C)(C)C)(C(C)(C)C)C(C)(C)C	18
**ODipp**	Oc1c(C(C)C)cccc1C(C)C	18
**OTerDipp**	Oc1c(c2c(C(C)C)cccc2C(C)C)cccc1c3c(C(C)C)cccc3C(C)C	13
**OBor**	Ob1n(ccn1c1c(cccc1C(C)C)C(C)C)c1c(cccc1C(C)C)C(C)C	13
**StBu**	SC(C)(C)C	23
**SDipp**	Sc1c(C(C)C)cccc1C(C)C	18
**STerDipp**	Sc1c(c2c(C(C)C)cccc2C(C)C)cccc1c3c(C(C)C)cccc3C(C)C	11
**PDipp**	P(c1c(cccc1C(C)C)C(C)C)c1c(cccc1C(C)C)C(C)C	26
**PAd**	P(C1(C2)C[C@H](C3)C[C@@H]2C[C@H]3C1)C1(C2)C[C@H](C3)C[C@@H]2C[C@H]3C1	25
**PMes**	P(c1c(C)cc(C)cc1C)c2c(C)cc(C)cc2C	25

**External test set**		
**PtBu** (PtBu)	CC(PC(C)(C)C)(C)C	9
**PArF** (P(ArF)2)	FC(C1=CC(PC2=CC(C(F)(F)F)=CC(C(F)(F)F)=C2)=CC(C(F)(F)F)=C1)(F)F	9
**Sad** (SAd)	SC12C[C@H]3C[C@@H](C1)C[C@@H](C2)C3	9
**BMes** (BMes2)	CC(C=C(C)C=C1C)=C1BC2=C(C)C=C(C)C=C2C	6
**B3** (B3)	CC1(C)C(C)(C)OBO1	8
**Pyr** (pyrimidine)	C1=CN=CN=C1	9
**An** (antracene)	C12=CC3=C(C=CC=C3)C=C1C=CC=C2	9
**TMP**(piperidine)	CC1(C)CCCC(C)(C)N1	9

1.3. Conformer cross-checks with CENSO

To confirm the reliability of our conformer treatment, we used the CENSO software package[34] with 7 of our silylenes. CENSO offers a multi-step refinement of conformer ensembles, gradually increasing the level of theory and removing high lying conformers. We performed CENSO up to Step0, where a B97-D3/def2-SVP[35,21] single point energy calculation is performed on every conformer obtained from CREST (GFN2-xTB(GBSA) level of theory). Figure S4 shows that for three of the seven silylenes a new conformer is the *minconf* after the CENSO treatment. For 2 silylenes, the CREST *minconf* has a significantly higher relative energy than the CENSO *minconf*. This indicates that the conformer treatment has a significant contribution to the error of our calculated free energy profile, as not all our selected *minconfs* are the “real” *minconfs*.

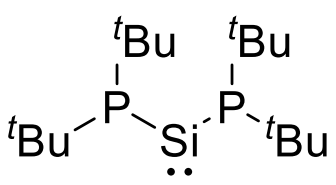

1065
ΔG = 3.48
N = 12

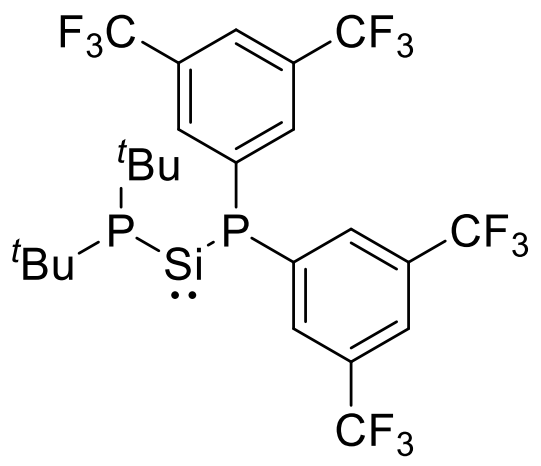

1109
ΔG = 0.00
N = 71

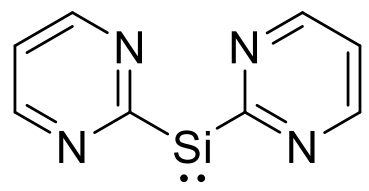

1251
ΔG = 0.00
N = 2

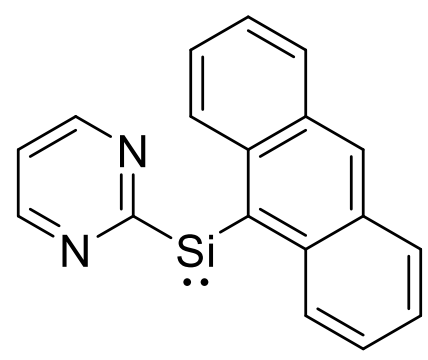

1299
ΔG = 0.00
N = 1

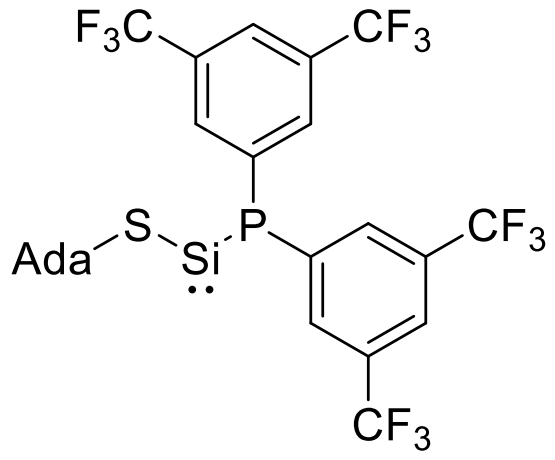

1345
ΔG = 0.24
N = 182

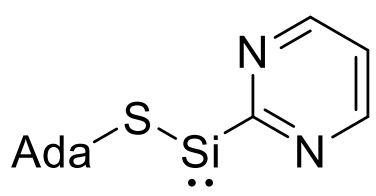

1348
ΔG = 0.00
N = 4

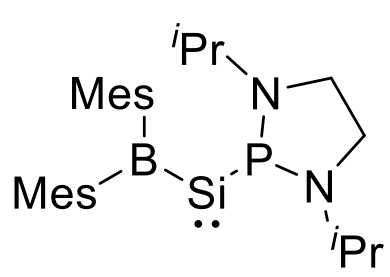

1396
ΔG = 4.96
N = 445

ΔG = *G* difference between CREST minconf and CENSO Step0 minconf in kcal/mol

N = Number of conformers

Figure S4: Silylenes, CENSO conformer treatment was used for, and the difference in relative energy before and after the CENSO treatment.

2. DFT-level descriptors

The features can be categorized in global and substituents specific features. “Global” means that this feature describes properties of the whole tetrylene or properties at the silicon/germanium center. For each tetrylene, a global feature exists only once. Substituent specific properties refer only to one substituent which is bound to the tetrylene. As each tetrylene possesses two substituents, each substituent specific property exists twice, one for **substituent1** and one for **substituent2**. To use those properties as features, we condensed the features to the following descriptors:

1. **sum:** the sum of feature(**substituent1**) and feature(**substituent2**)
2. **min:** the minimum value of both substituent features
3. **max:** the maximum value of both substituent features

2.1. Global features

Table S2. Global features used in this study. E = Si/Ge, L = Substituent

Feature name	Full name	Group	Unit
**LEL_angle**	Angle between R^1, E and R^2	Angle	degrees
**%vbur**	Percent buried volume	Buried Volume	
**vbur**	Buried Volume	Buried Volume	Å^3
**vtot**	Molecular vdW volume from buried volume calculation	Buried Volume	Å^3
**qvbur_max**	Highest quadrant buried volume	Buried Volume	Å^3
**qvtot_max**	Highest quadrant total volume	Buried Volume	Å^3
**ovbur_max**	Highest octant buried volume	Buried Volume	Å^3
**ovtot_max**	Highest octant total volume	Buried Volume	Å^3
**qvbur_min**	Lowest quadrant buried volume	Buried Volume	Å^3
**qvtot_min**	Lowest quadrant total volume	Buried Volume	Å^3
**ovbur_min**	Lowest octant buried volume	Buried Volume	Å^3
**ovtot_min**	Lowest octant total volume	Buried Volume	Å^3
**qvbur_max_delta**	Highest buried volume difference between two neighboring quadrants	Buried Volume	Å^3
**qvtot_max_delta**	Highest total volume difference between two neighboring quadrants	Buried Volume	Å^3
**ovbur_max_delta**	Highest buried volume difference between two octants	Buried Volume	Å^3
**ovtot_max_delta**	Highest total volume difference between two	Buried Volume	Å^3

	octants		
**ratio_vbur_vtot**	Ratio of buried and total volume	Buried Volume	
**Dipole_moment**	Normalized Dipole Moment	Dipole	Debye
**HOMO**	HOMO energy	Frontier Orbital derived	eV
**LUMO**	LUMO energy	Frontier Orbital derived	eV
**HOMO_LUMO_gap**	HOMO-LUMO gap, Molecular softness	Frontier Orbital derived	eV
**Energy_HOMO(N+1)**	HOMO energy of radical anion	Frontier Orbital derived	eV
**Energy_HOMO(N-1)**	HOMO energy of radical cation	Frontier Orbital derived	eV
**Chem_pot**	Chemical Potential	Frontier Orbital derived	eV
**s- @E**	Local softness parameter s- at atom E	Fukui	
**s+ @E**	Local softness parameter s+ at atom E	Fukui	
**s0 @E**	Local softness parameter s0 at atom E	Fukui	
**f- @E**	Atom-condensed fukui parameter f- for atom E	Fukui	
**f+ @E**	Atom-condensed fukui parameter f+ for atom E	Fukui	
**f0 @E**	Atom-condensed fukui parameter f0 for atom E	Fukui	
**CDD @E**	Atom-condensed fukui parameter CDD for atom E	Fukui	
**Charge @E**	NBO Partial charge of E	NBO derived	e^-
**E_LP_s_char**	Percent s-character of lone pair at E atom	NBO derived	%
**E_LP_occ**	Occupation of lone pair at E atom	NBO derived	
**Electrophilicity @E**	Local electrophilicity at atom E	Fukui	eV
**Electrophilicity**	Electrophilicity	Radical derived	eV
**IP**	Ionization Potential	Radical derived	eV
**EA**	Electron affinity	Radical derived	eV
**Hardness**	Hardness, Fundamental gap	Radical derived	eV
**Softness**	Softness	Radical derived	eV^{-1}
**SASA_area**	Molecular solvent accessible surface area	SASA	$Å^2$
**SASA_volume**	Volume enclosed by the molecular solvent accessible surface	SASA	$Å^3$
**sasa_area @E**	E atom solvent accessible surface area	SASA	$Å^2$
**sasa_volume @E**	E atom solvent accessible surface volume	SASA	$Å^3$
**Sterimol_L(red)**	Reduced Sterimol L value	Sterimol	Å
**Sterimol_B_1**	Sterimol B_1 value	Sterimol	Å
**Sterimol_B_5**	Sterimol B_5 value	Sterimol	Å

2.2. Substituent specific features

Table S3. Substituent specific features used in this study. E = Si/Ge, L = Substituent.

Full name	Group	Unit	Feature name sum	Feature name min	Feature name max
Distance between silicon atom and neighboring atom of substituent	Distance	Å	**EL_dist**	**EL_dist_min**	**EL_dist_max**
Percent buried volume of substituent	Buried Volume		**%vbur_L**	**%vbur_L_min**	**%vbur_L_max**
Sterimol B_1 value of substituent	Sterimol	Å	**Sterimol_B_1 @L**	**Sterimol_B_1 @L_min**	**Sterimol_B_1 @L_max**
Sterimol B_5 value of substituent	Sterimol	Å	**Sterimol_B_5 @L**	**Sterimol_B_5 @L_min**	**Sterimol_B_5 @L_max**
Sterimol L value of substituent	Sterimol	Å	**Sterimol_L @L**	**Sterimol_L @L_min**	**Sterimol_L @L_max**
Local solvent accessible surface area at atom of substituent which is bound to the silicon atom	SASA	$Å^2$	**sasa_area @L_1**	**sasa_area @L_1_min**	**sasa_area @L_1_max**
Local solvent accessible surface volume at atom of substituent which is bound to the silicon atom	SASA	$Å^3$	**sasa_volume @L_1**	**sasa_volume @L_1_min**	**sasa_volume @L_1_max**
Atom-condensed fukui parameter f- for atom of substituent which is bound to the silicon atom	Fukui		**f+ @L_1**	**f+ @L_1_min**	**f+ @L_1_max**
Atom-condensed fukui parameter f+ for atom of substituent which is bound to the silicon atom	Fukui		**f- @L_1**	**f- @L_1_min**	**f- @L_1_max**
Atom-condensed fukui parameter f0 for atom of substituent which is bound to the silicon atom	Fukui		**f0 @L_1**	**f0 @L_1_min**	**f0 @L_1_max**
Atom-condensed fukui parameter CDD for atom of substituent which is bound to the silicon atom	Fukui		**CDD @L_1**	**CDD @L_1_min**	**CDD @L_1_max**
Local softness parameter s- at atom of substituent which is bound to the silicon atom	Fukui		**s+ @L_1**	**s+ @L_1_min**	**s+ @L_1_max**
Local softness parameter s+	Fukui		**s- @L_1**	**s- @L_1_min**	**s- @L_1_max**

at atom of substituent which is bound to the silicon atom					
Local softness parameter s0 at atom of substituent which is bound to the silicon atom	Fukui		**s0 @L_1**	**s0 @L_1_min**	**s0 @L_1_max**
Local electrophilicity at atom of substituent which is bound to the silicon atom	Fukui	eV	**Electrophilicity @L_1**	**Electrophilicity @L_1_min**	**Electrophilicity @L_1_max**
Partial charge of atom of substituent which is bound to the silicon atom	NBO derived	e^-	**Charge @L_1**	**Charge @L_1_min**	**Charge @L_1_max**
Wiberg Bond Index of Bond between silicon atom and neighboring atom of substituent	NBO derived		**EL BO**	**EL BO_min**	**EL BO_max**

2.3. Definition of steric features

2.3.1. Angles and distances

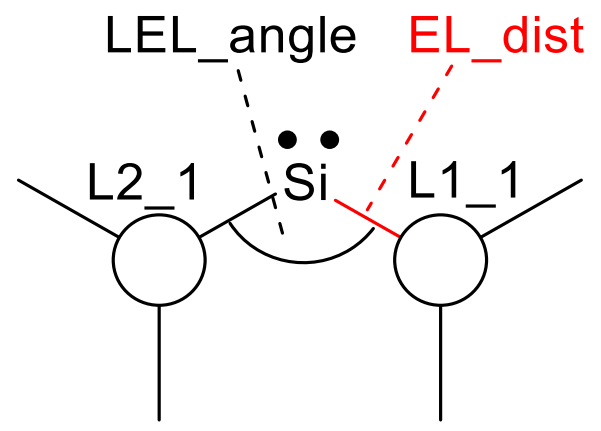

Figure S5. Representation of angle (LEL angle) and bond distance (EL_dist) which are used as descriptors.

LEL_angle: Angle in ° between L1, E(=Si, Ge) and L2.
EL_dist: Distance in Å between **L_1** (first atom) and **E** (= Si, Ge).

2.3.2. Buried Volume

The buried volume is a measure of the steric hindrance in the first coordination sphere induced by a ligand L of a transition metal complex.[12] The percent buried volume is the proportion of a sphere with radius r centered on M, which is buried by overlap with the atoms of the ligand. In case of the tetrylenes, E(=Si, Ge) is formally used as center of the sphere. The buried volume and all derived properties were measured via MORFEUS[13], using the recommended specifications: Atomic radii: Bondi scaled by 1.17; sphere radius: 3.5 Å, mesh spacing for numerical integration: 0.001, exclude H atoms. In addition to buried volume (**vbur**) the total ligand volume (**vtot**) was calculated using the same settings, but with a sphere encompassing the entire molecule.

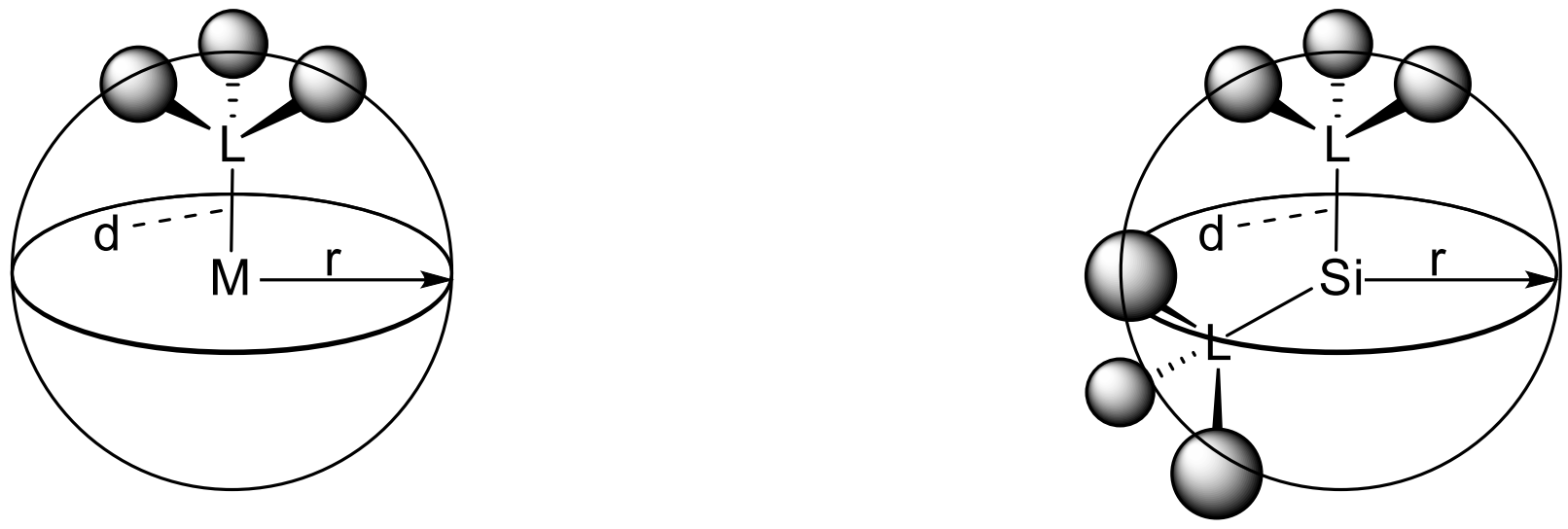

Figure S6. Representation of buried volume (left) and buried volume in this work.

Buried Volume Descriptors:

- Percent buried volume: **%vbur**,
- Percent buried volume of L1: **%vbur (L1)** -> atoms of L2 excluded,
- Percent buried volume of L2: **%vbur (L2)** -> atoms of L1 excluded
- **vbur:** buried volume in Å^3

Total Ligand Volume Descriptors:
vtot: total ligand volume in Å^3
ratio_vbur_vtot:
vbur/**vtot**

Quadrant and octant buried and total volume
The quadrants are defined by x- and y-axis, octants are additionally defined by z-axis. In tetrylenes the origin and axes are defined as follows to obtain consistent descriptors: The origin is located on E(=Si, Ge), the z-axis from E(=Si, Ge) through the middle of L1_1 and L2_1, the xz-plane by Si and L1_1.

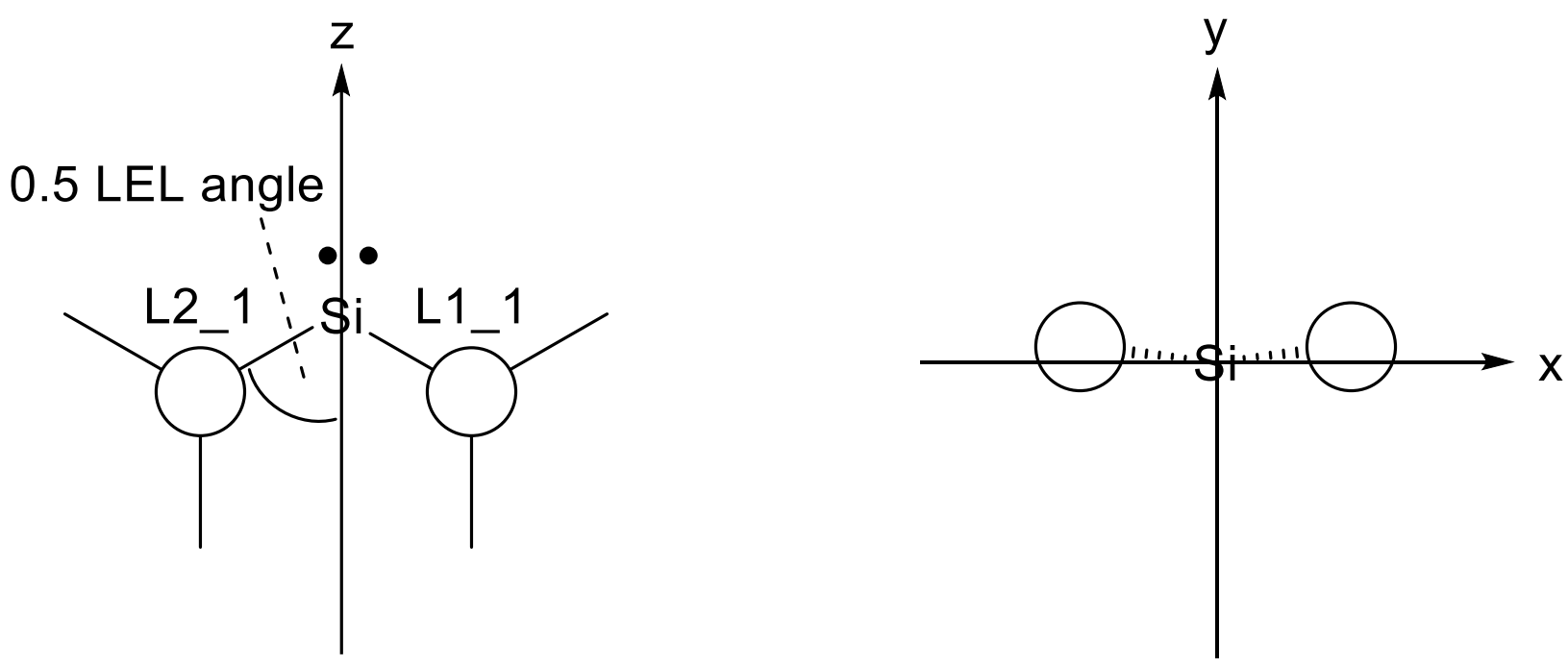

Figure S7. Representation of axes used for quadrants and octants. E (= Si, Ge) was chosen to be the origin.

For each quadrant/octant the buried and total volume were determined to obtain the following descriptors:

- **qvbur_min**, **qvbur_max:** minimum and maximum buried volume of quadrants (in Å^3)
- **qvtot_min**, **qvtot_max:** minimum and maximum total ligand volume of quadrants (in Å^3)
- **qvbur_max_delta: qvbur_max – qvbur_min**
- **ovbur_min**, **ovbur_max:** minimum and maximum buried volume of octants (in Å^3)

- **ovtot_min**, **ovtot_max:** minimum and maximum total ligand volume of octants (in $Å^3$)
- **ovbur_max_delta: ovbur_max – ovbur_min**

2.3.3. Sterimol

The Sterimol descriptors are measures of the steric size of a substituent. They were introduced by Verloop in 1976 for QSAR modeling.[14] The atom attached to the substituent in the calculation (by definition H) is atom1 and the first atom in the substituent is defined as atom2. Important Sterimol parameters are the depth of the substituent L, the minimum rotational size B_1 and maximum rotational size B_5 of the substituent. Historically, L is corrected by adding 0.40 Å.

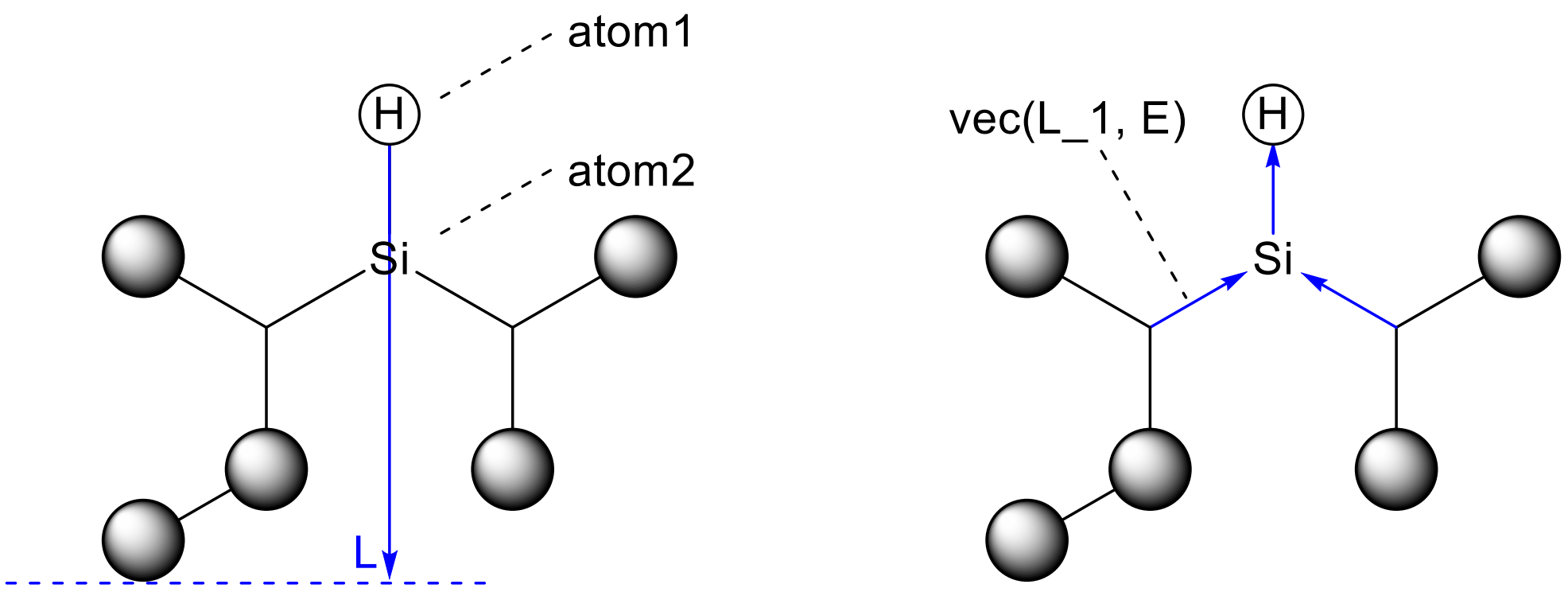

Figure S8. a) The Sterimol parameter L (depth of a substituent) is normally defined by the length of a vector going from atom1, through atom2 and ending on the tangent of the vdW surface (left). b) Determination of H position is done by normalized vectors $\overrightarrow{\mathbf{L1\_1E}}$ and $\overrightarrow{\mathbf{L2\_1E}}$.

In this work, two different types of Sterimol parameters were determined. For calculation of all parameters MORFEUS[13] was used.

1) The complete silylene is treated as the substituent. For determination of the parameters a hydrogen atom is added as atom1. The normalized vectors $\overrightarrow{\mathrm{L1\_1E}}$ and $\overrightarrow{\mathrm{L2\_1E}}$ are used to determine the position of H. E (= Si, Ge) is atom2.
   Descriptors:
   Sterimol_L_red: The depth L is determined without correction of L. L is then subtracted by the distance of atom1 and atom2. L in Å.
   Sterimol_B_1: B_1 in Å
   Sterimol_B_5: B_5 in Å

2) The Sterimol parameters are determined for each ligand in the silylene. The central silicon atom is defined as atom1, the first atom of the ligand, which is connected to the silicon center, is defined as atom2.
   Sterimol_ligand_L: The depth L is determined without correction of L.
   Sterimol_ligand_B_1: B_1 in Å
   Sterimol_ligand_B_5: B_5 in Å

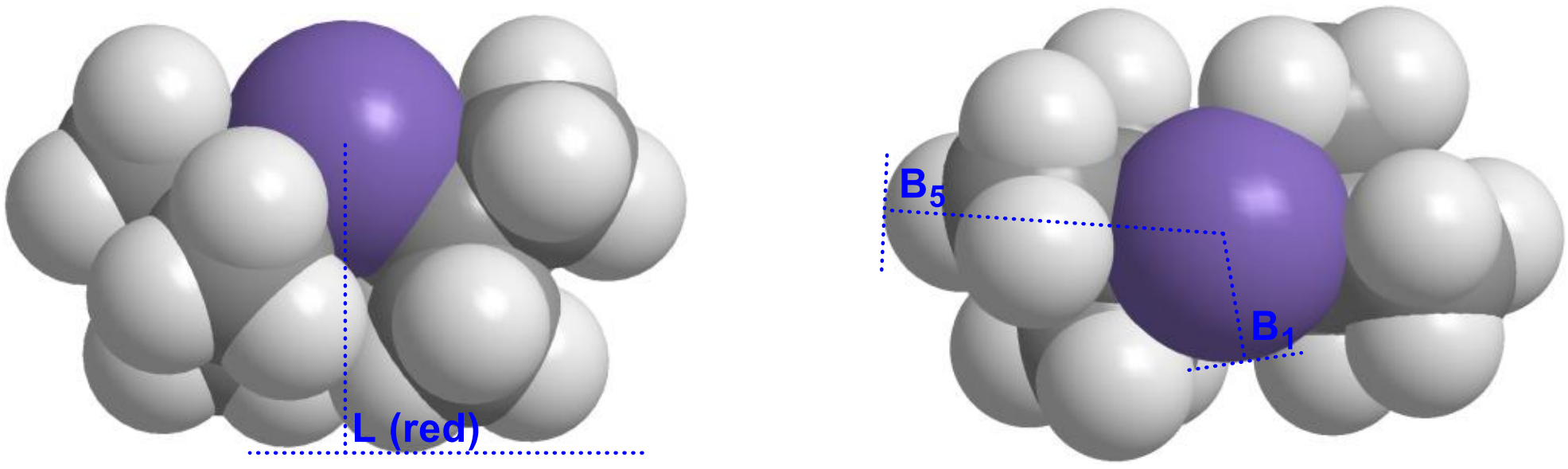

Figure S9. Sterimol parameters used in this work.

2.3.4 Solvent accessible surface area (SASA)

The solvent accessible surface area (SASA) describes the area of a molecule which is available to a solvent. The probe radius is often chosen to be 1.4 Å as it represents water. The atomic SASA can be used as a measure for the steric availability of an atom. The SASA and atomic SASA as well as the SASA volume and atomic SASA volume were calculated via MORFEUS, where a modified version of the method of Shrake and Rupley is used,[15] and atomic SASA and volumes are computed as described by Eisenhaber and coworkers.[16]

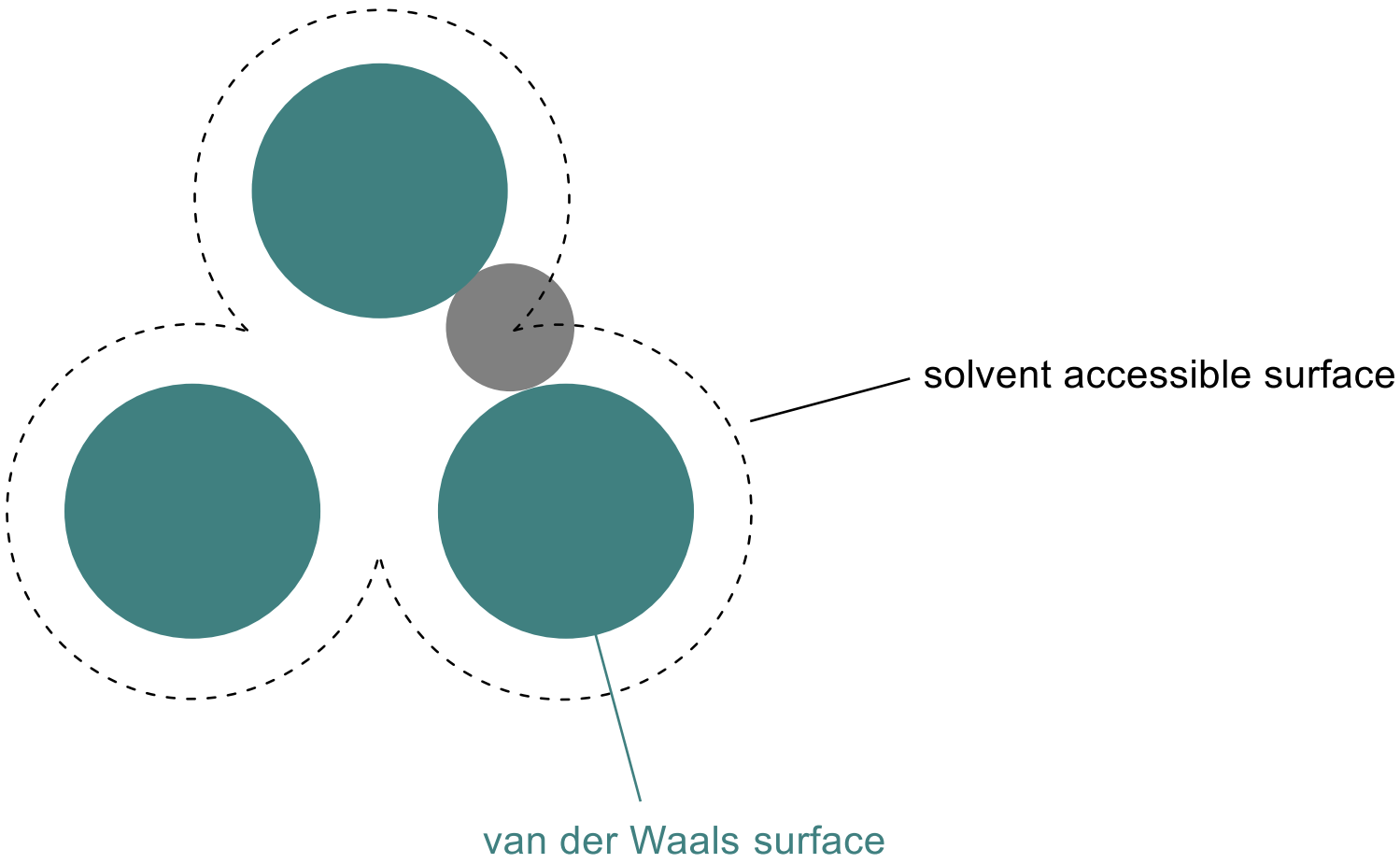

Figure S10. Representation of solvent accessible surface area.

SASA_area: solvent accessible surface area
SASA_volume: solvent accessible volume
sasa_area @E: atomic solvent accessible surface area at **E** (= Si, Ge)
sasa_volume @E: atomic solvent accessible volume at **E** (=Si, Ge)
sasa_area @L_1: atomic solvent accessible surface area at first atom of the ligand **L_1** which is attached to **E** (= Si, Ge)
sasa_volume @L_1: atomic solvent accessible volume at first atom of the ligand **L_1** which is attached to **E** (= Si, Ge)

2.4 Definition of electronic features

2.4.1 Frontier orbital-derived

- **HOMO:** energy of highest occupied (Kohn-Sham) orbital, “HOMO”
- **LUMO:** energy of lowest virtual (Kohn-Sham) orbital, “LUMO”
- **HOMO_LUMO_gap:** $E(LUMO) - E(HOMO)$

2.4.2 Radical derived

The following global molecular descriptors make use of the radical cation/anion.[17]

- **IP:** ionization potential
- **EA:** electron affinity
- **Hardness:** $\eta = IP - EA$
- **Softness:** $S = \frac{1}{\eta}$
- **Chem_pot:** Chemical potential $\mu = -\frac{IP+EA}{2}$
- **Electrophilicity:** Global electrophilicity parameter $\omega = \frac{\mu^2}{2\eta}$

2.4.3 Fukui parameters

Atom-condensed Fukui indices were calculated via Multiwfn.[18] The Fukui coefficient for electron removal/ coefficient for electrophilic attack and is a measure for nucleophilicity. The coefficient for electron addition/coefficient for nucleophilic attack is a measure for electrophilicity. The coefficient for radical attack is often used for radical reactivity.[17]

- **f- (@E/@L_1):** coefficient for electron removal $f^- = q_{N-1} - q_N$ at E(= Si, Ge)/at first atom of ligand **L_1**
- **f+ (@E/@L_1):** coefficient for electron addition $f^+ = q_N - q_{N+1}$ at E(= Si, Ge)/at first atom of ligand **L_1**
- **f0 (@E/@L_1):** coefficient for radical attack $f^0 = \frac{q_{N-1}-q_{N+1}}{2}$ at E(= Si, Ge)/at first atom of ligand **L_1**
- **CDD (@E/@L_1):** coefficient for radical attack $f^{(2)} = f^+ - f^- = 2q_N - q_{N-1} - q_{N+1}$ at E(= Si, Ge)/at first atom of ligand **L_1**
- **s- (@E/@L_1):** $s^- = S \cdot f^-$
- **s+ (@E/@L_1):** $s^+ = S \cdot f^+$
- **s0 (@E/@L_1):** $s^0 = S \cdot f^0$
- **Electrophilicity (@E/@L_1):** $local\ electrophilicity = f^+ \cdot \omega$

2.4.4 NBO derived parameters

- **Charge @E:** Natural charge at E(= Si, Ge)
- **E_LP_s_char:** s-character of lone pair at E(= Si, Ge)
- **E_LP_occ:** occupation of lone pair at E(= Si, Ge)
- **Charge @L_1:** Natural charge at first atom of ligand L_1
- **EL_BO:** Wiberg Bond Index of E–L_1 bond

2.4.5 Further properties

Dipole_moment: norm of dipole moment

3 Data Science

3.1. Principal component analysis (PCA)

Principal component analyses to initially assess the dependency of the activation energy on the approx. 100 DFT-level descriptors were performed (Figure S11).

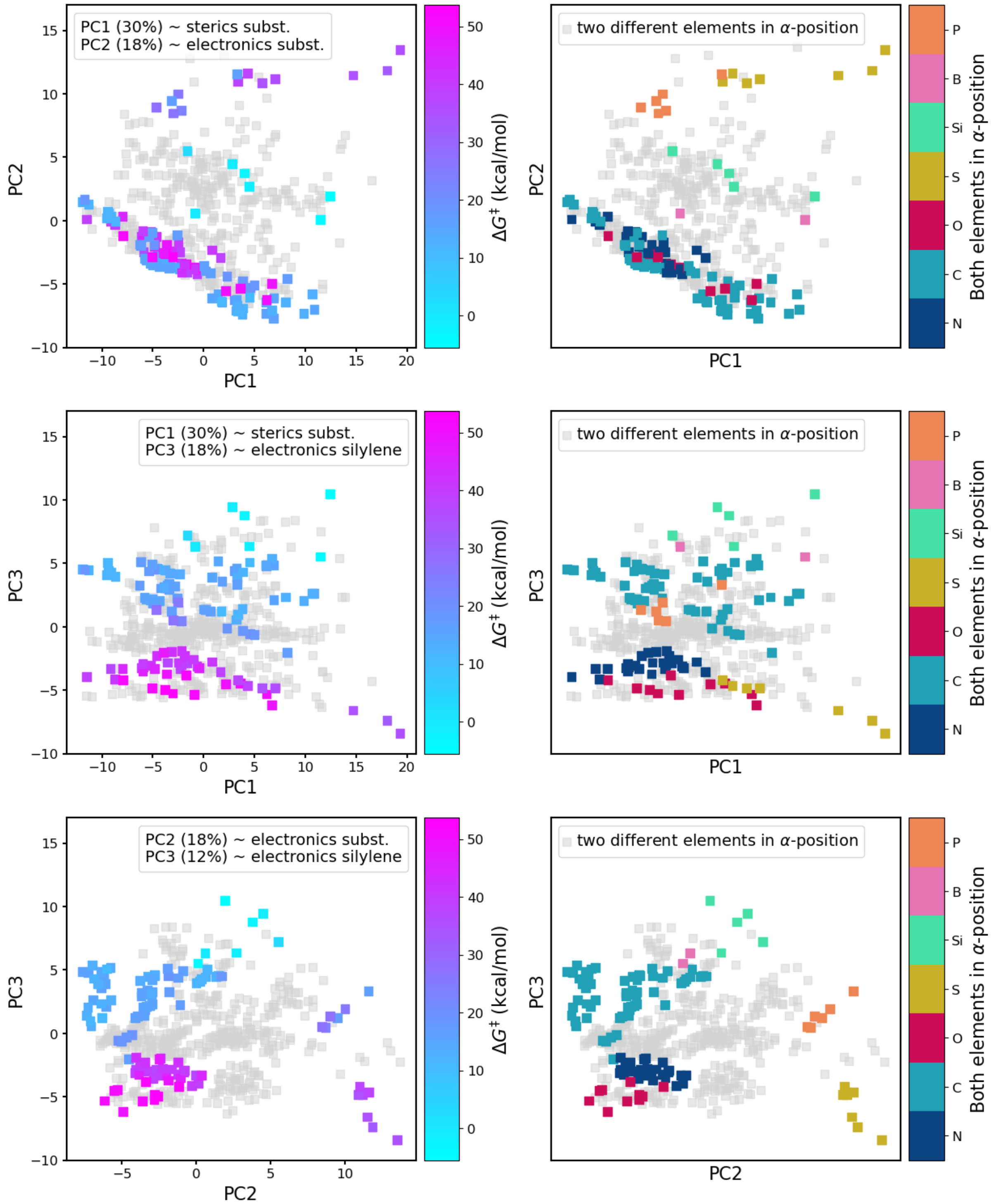

Figure S11: Scatterplots of the first three principal components obtained from all our DFT-level descriptors for all silylenes. Color-coding on the left side is the activation barrier of H_2 activation with silylenes, on the right side it is the element in α-position. Grey dots have mixed elements in α-position, colored dots have the respective element in α-position two times.

By color coding the silylenes in their respective activation barrier (Figure S11, left side) we aimed at identifying chemical space regions of particularly high or low reactivity. While plots using the first principal component, which mainly consists of steric descriptors, showed no meaningful separation of the activation barriers, a plot using the second and third principal component, which represent the electronic properties of the silylenes, showed a very good separation with high and low activation barrier regions. This already indicates that the activation barrier mainly is determined by electronic properties. Silylenes with two identical elements in α-position distribute in clusters along the edges while these with two different elements in α-position are scattered in between. Interestingly, within the clusters of one α-element no significant variation of the activation barriers is visible, while there are great differences between the different clusters. Color coding of the same principal component plot by the element in α-position to the silylene highlights that (Figure S11, right side). These findings motivated the Bag of Elements approach.

3.2 Bag of Elements: *ΔG‡* for silylenes

The bag of elements is a matrix with a column for each unique substituent of the substituents in the scope of this work. Each row represents a silylene/germylene with each matrix element expressing how often (0, 1, 2) each substituent is present in the respective silylene/germylene. The bag of substituents was used before by Tolman and Gensch. [19,20]

Linear regression with ridge regularization (scikit-learn[21]) was used to model the barrier and thermodynamics of single-site dihydrogen activation using the bag of elements in α-position. The amount of L2 regularization was optimized for each regression using α-values between 10^{-3} and 10^{2}. A 70:30 random train-test split was used. To ensure stability and robustness in performance, Leave-One-Out, Leave-One-Substituent-Out, and 200 times repeated 4-fold cross-validations were performed. In Figure S12 as well as all following illustrations of our models, a performance metrics is shown, summarizing the R^2 score as well as the mean absolute error of each validation method.

The **MAE** represents the average magnitude of errors between predicted and actual values, regardless of their direction. The smaller the MAE the more accurate the prediction.

R^2 (R-squared, or the coefficient of determination) quantifies how much of the variability in the target variable is captured by the regression model. It is defined as the proportion of the total variance that is explained by the model's predictions and is defined by the following equation:

$$R^2 = 1 - \frac{\mathrm{SS_{res}}}{\mathrm{SS_{tot}}}$$

Where SS_{res} is the sum of squared residuals and SS_{tot} the total sum of squares:

$$\mathrm{SS_{res}} = \sum_{i=1}^{n}(y_i - \hat{y}_i)^2 \qquad \mathrm{SS_{tot}} = \sum_{i=1}^{n}(y_i - \bar{y})^2$$

y_i = Actual value

$\hat{y}_i$ = Predicted value

$\bar{y}$ = Mean of actual values

An R^2 = 1 refers to a model, that perfectly explains the varition, wheras a model with R^2 = 0.5 only explains 50% of the variation. A negative value for R^2 means that the model performs worse than a horizontal line (i.e. predicting the mean).

Element increments

The final increments for the seven elements for the reaction energy of H_2 activation by silylenes were obtained from the fit by the element coefficients and intercept. As each silylene always contains two substituents the increment was calculated as: ΔG(element) = coefficient(element) + 0.5·intercept. The same approach was applied for calculating the increments for the activation energy as well as the reaction profile of H_2 activation with germylenes.

Influence of different train-test splits

Initially, we employed a 70:30 train-test split for the Bag of Elements (BoE) model, which was trained using linear Ridge regression with a regularization parameter α of 1.44 (see Figure 4 in the manuscript and Figure S12). To assess whether the selected 70:30 train:test split affected model performance due to the random partitioning of the data, we performed additional robustness analyses. These included several cross-validation approaches as well as Ridge regression models trained using different train:test ratios ranging from 70:30 to 20:80. Figure S12 summarizes the Ridge regression results based on the BoE descriptors and illustrates the corresponding element increments obtained for the different train:test ratios. Notably, robust model performance was maintained across all tested partitioning schemes, even for the more strongly imbalanced splits. Moreover, the element increments remained highly consistent across the different train:test ratios, with both their overall trends and signs being preserved. These results indicate that the model performance and the derived elemental increments are not substantially affected by the specific train:test partition selected.

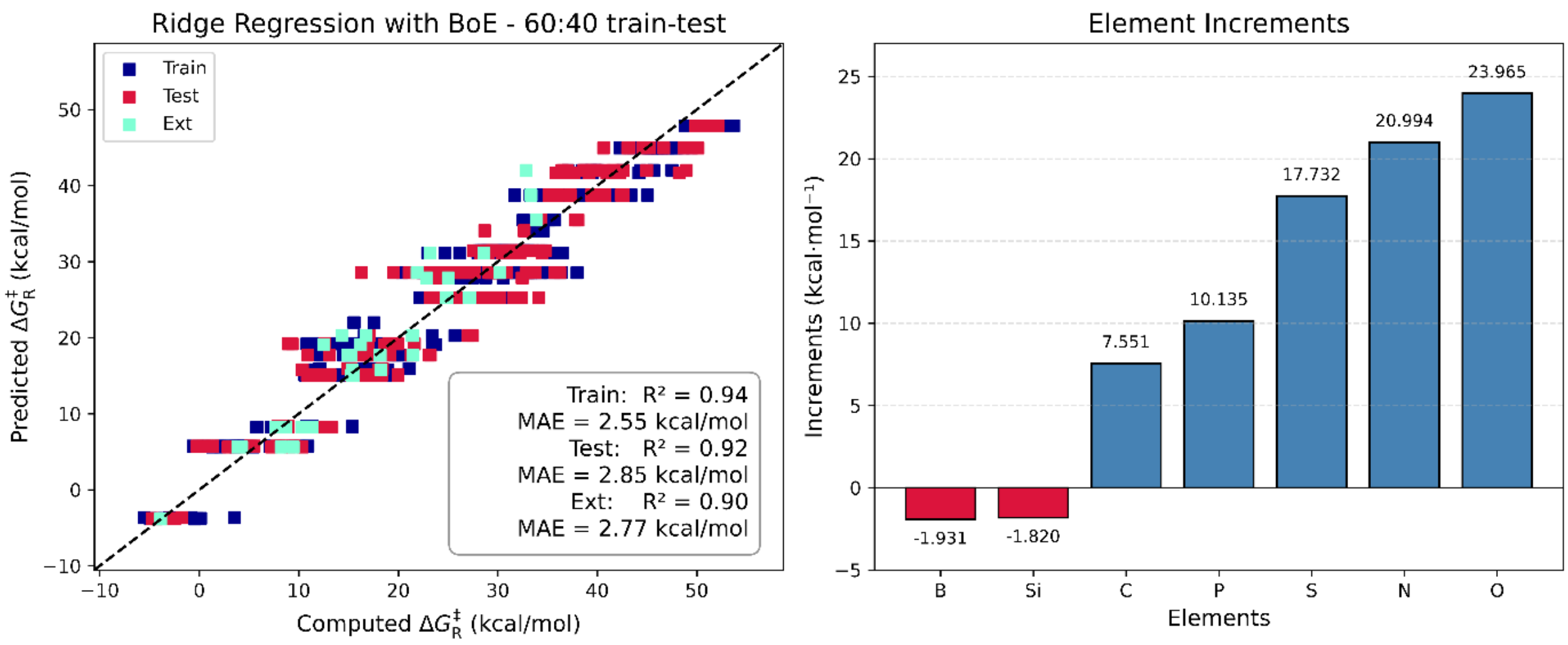

Figure S12: Bag of Elements (BoE) models with a linear Ridge regression (α = 1.44) for the activation barrier on the one-hot encoding of the element in α-position to the silicon with different random train-test splits from 60:40 to 20:80. Continued on next page.

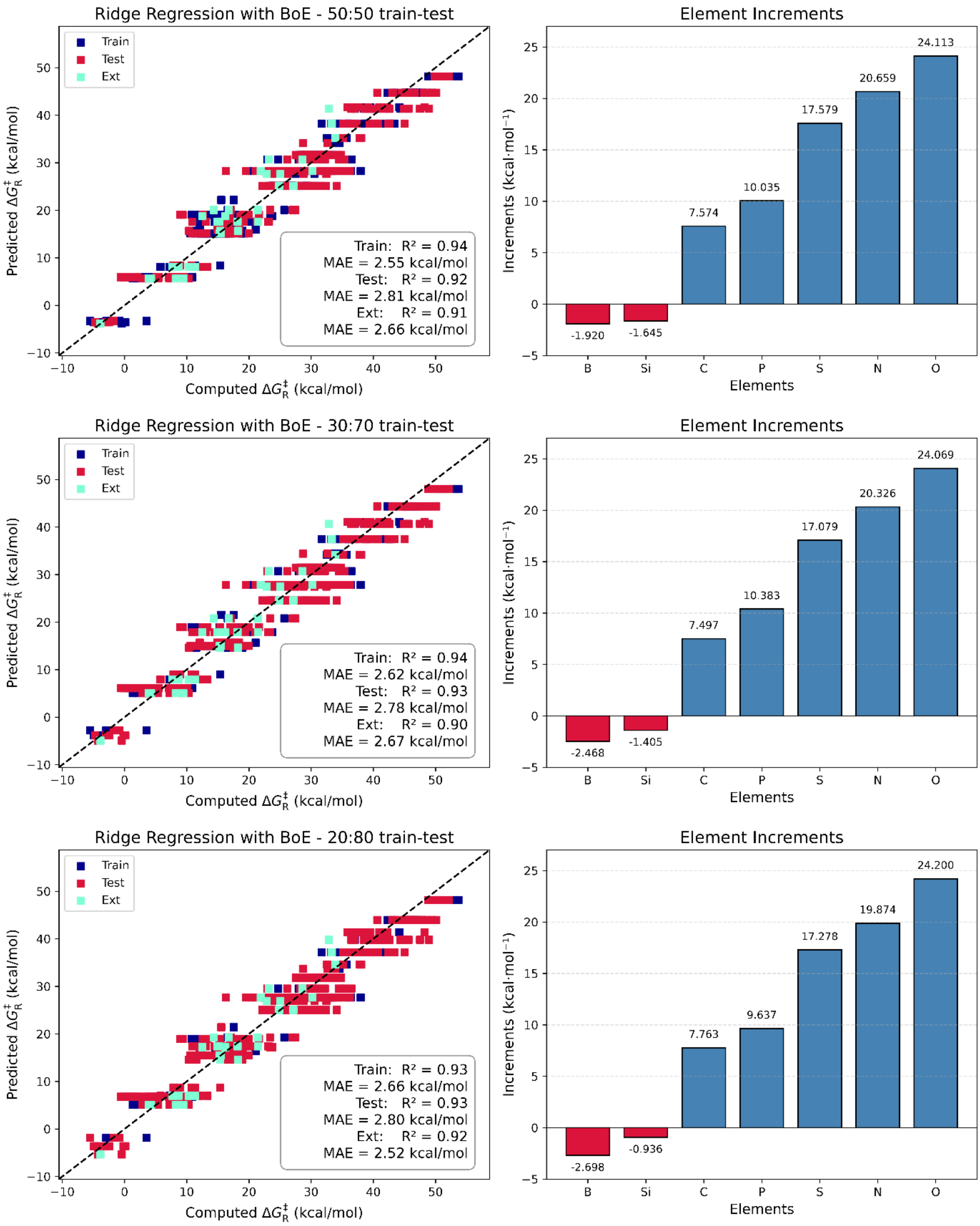

Figure S12_continued: Bag of Elements (BoE) models with a linear Ridge regression (α = 1.44) for the activation barrier on the one-hot encoding of the element in α-position to the silicon with different random train-test splits (random state = 42) ranging from 60:40 to 20:80 train-test ratio.

Next to the dependence of the performance metrics and increments on the train-test split, we assessed the stability of the derived increments with respect to the composition of the training data by repeating the Ridge regression for 1,000 randomized train/test splits, using 70% of the data for training and 30% for testing. The regularization parameter was kept fixed at α = 1.44.

For each split, the regression coefficients and intercept were recalculated, and the corresponding increments were determined.

The distributions of the resulting increments are shown in Figure S13 and S14, statistical analysis is given in Table S4. All increments retained the same sign across all 1,000 splits: the Si and B increments were consistently negative, whereas the C, P, S, N, and O increments were consistently positive. The increments for C, P, S, N, and O exhibit only small variations across the different train/test splits, with coefficients of variation ranging from 0.007 to 0.027. The B and Si increments show somewhat larger relative variations (CV = 0.209 and 0.130, respectively), but their distributions remain clearly separated from zero. Overall, the analysis demonstrates that the sign and qualitative trends of the derived increments are robust with respect to variations in the composition of the training and test sets, while the absolute magnitude of B and Si exhibits somewhat greater sensitivity.

Table S4. Results of the analysis of the stability of increments with respect to changes in the dataset composition.

Element	Increment_ mean	Increment_ std	Increment_ median	Increment_ 2.5%	Increment_ 97.5%	CV	positive _%	negative _%
Si	-2,10885	0,273275	-2,10966	-2,64207	-1,58245	0,129585	0	100
B	-1,52281	0,318576	-1,52832	-2,14182	-0,82862	0,209202	0	100
C	7,542541	0,089853	7,539847	7,365846	7,715652	0,011913	100	0
P	10,45482	0,281816	10,45965	9,894908	11,0187	0,026956	100	0
S	18,02107	0,178696	18,01259	17,68251	18,37188	0,009916	100	0
N	20,59958	0,146291	20,59547	20,31943	20,8847	0,007102	100	0
O	23,899	0,16892	23,90036	23,55026	24,20316	0,007068	100	0

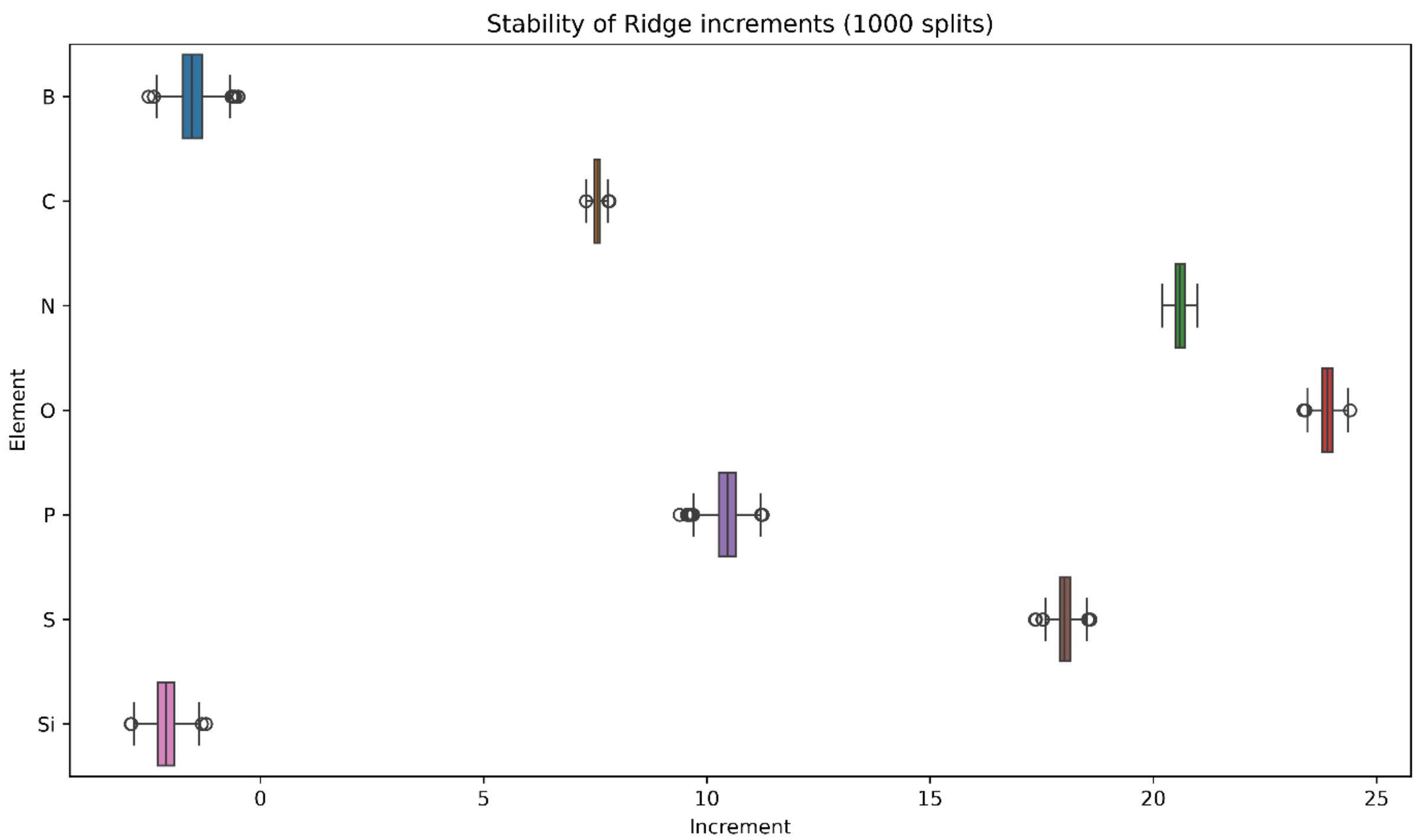

Figure S13. **Distribution of the derived increments across 1,000 randomized train/test splits.** Boxplot, showing the distributions of the increments obtained from Ridge regression using 70% of the data for training and 30% for testing (α = 1.44).

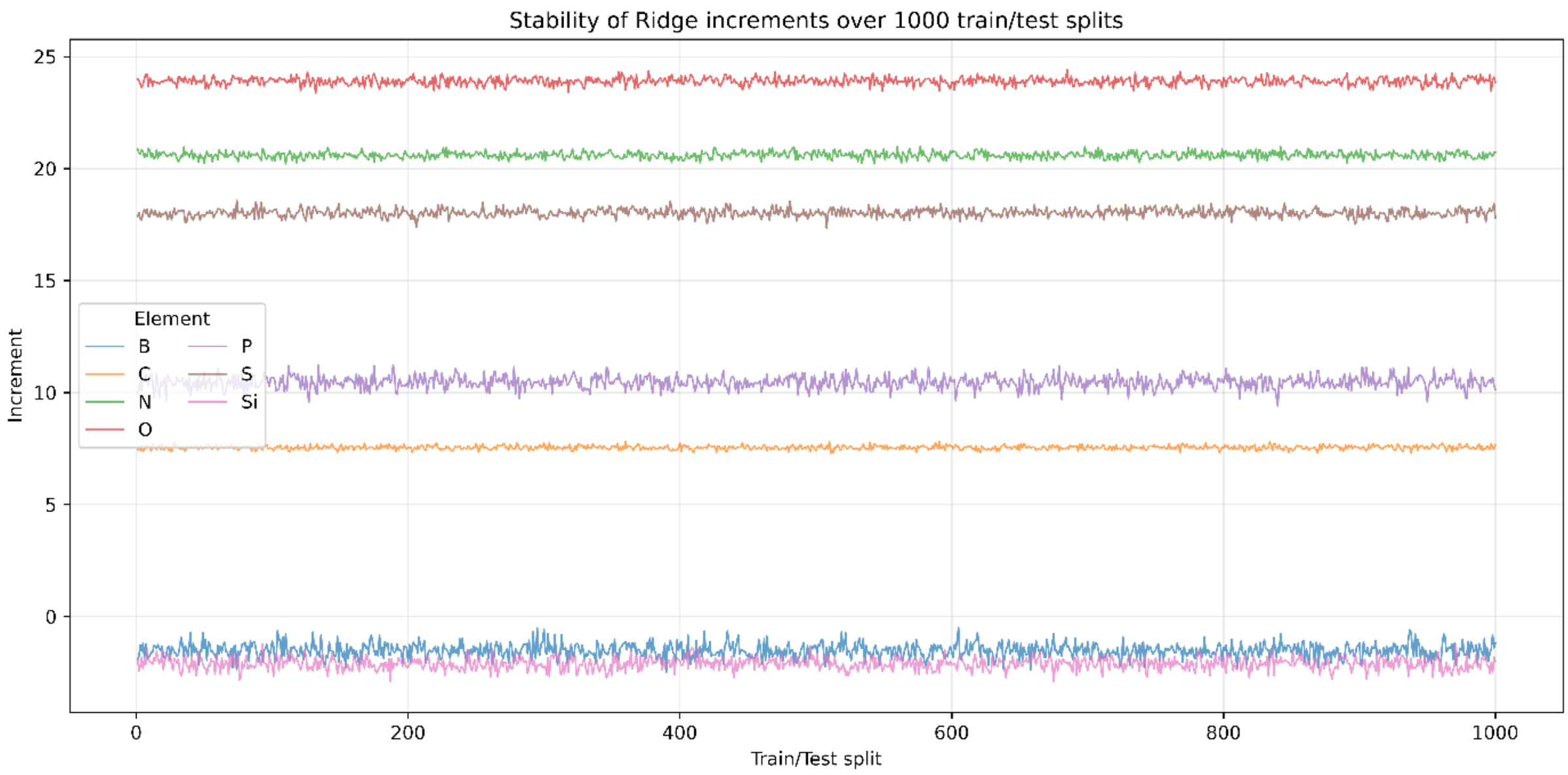

Figure S14. **Distribution of the derived increments across 1,000 randomized train/test splits.** Individual increment values obtained for each element from the 1,000 randomized train/test splits (70% training, 30% testing; α = 1.44).

Lower limit of training points

To test the lower number of training points required for training our model, we also tested – next to various cross-validation methods – a 5:95 train-test split (Figure S14). Even with a random train-test split of 5:95 from various seeds good and robust models were obtained.

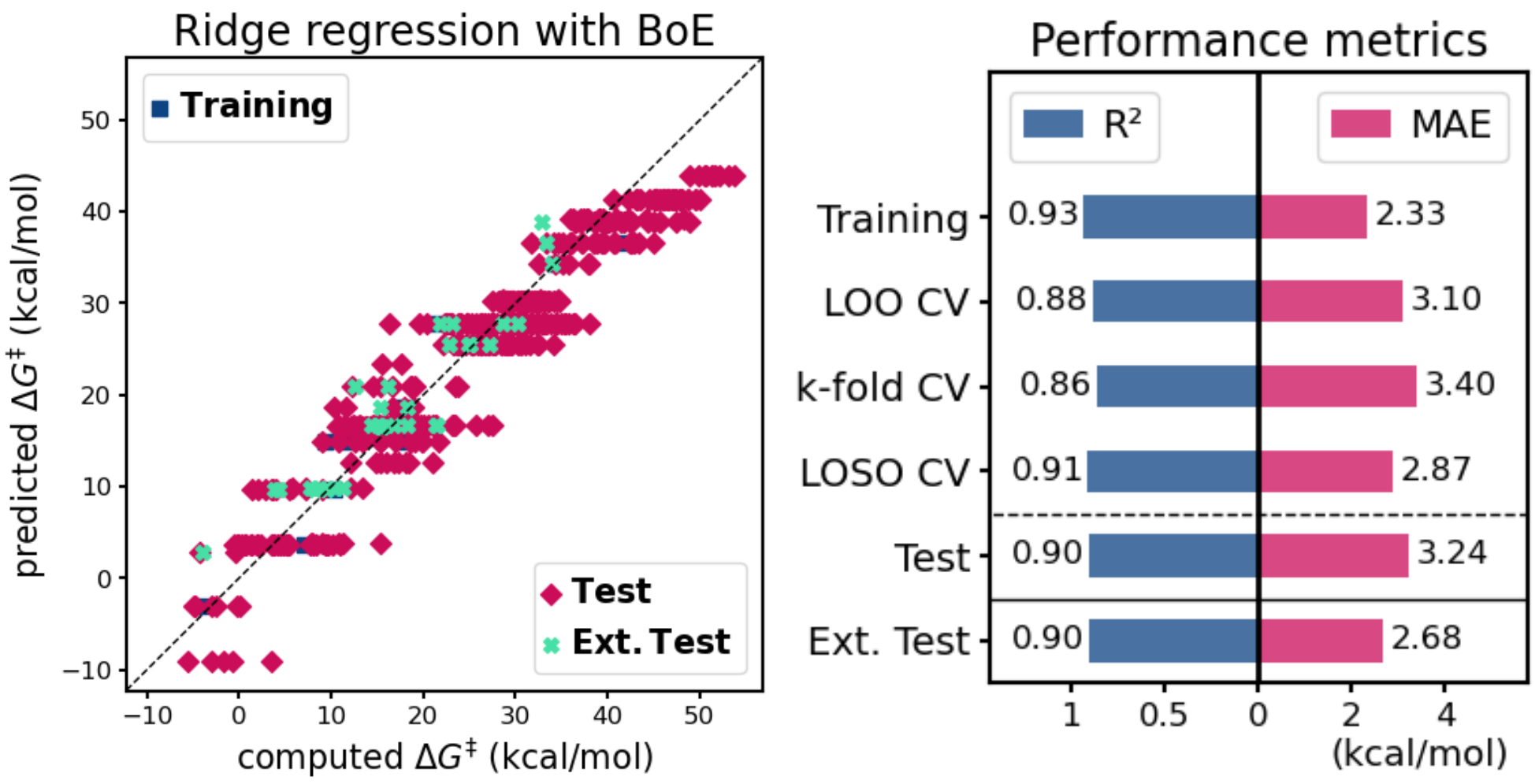

Figure S15: Bag of Elements (BoE) models with a linear Ridge regression (α = 1.44) for the activation barrier on the one-hot encoding of the element in α-position to the silicon with three different 5:95 random train-test splits. LOO CV: Leave-One-Out cross-validation. k-fold CV: 200 times repeated 4-fold cross-validation. LOSO CV: Leave-One-Substituent-Out cross-validation.

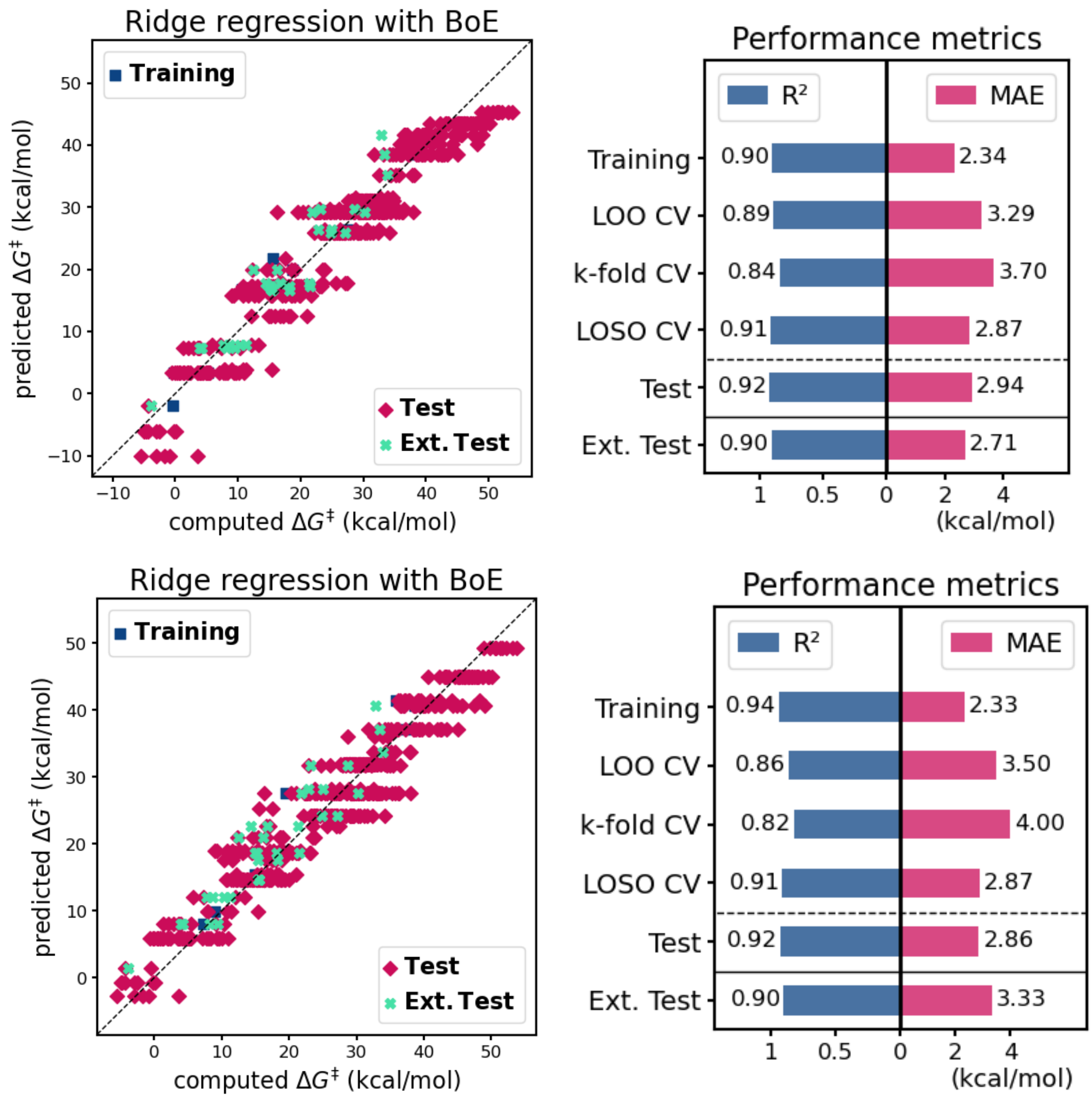

Figure S15continued: Bag of Elements (BoE) models with a linear Ridge regression (α = 1.44) for the activation barrier on the one-hot encoding of the element in α-position to the silicon with three different 5:95 random train-test splits. LOO CV: Leave-One-Out cross-validation. k-fold CV: 200 times repeated 4-fold cross-validation. LOSO CV: Leave-One-Substituent-Out cross-validation

3.3 Bag of Elements: ΔG_R for silylenes

For a 70:30 train-test split the following increments were obtained for the reaction energy:

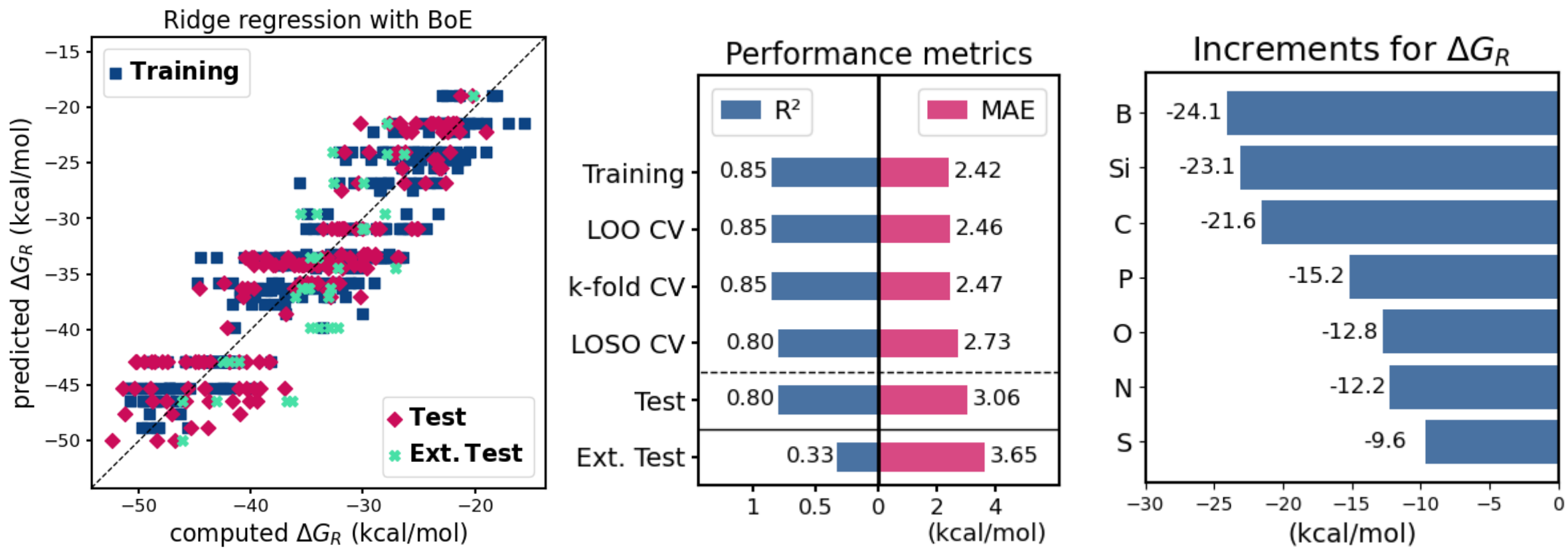

Figure S16: Bag of Elements (BoE) model with a linear Ridge regression (α = 1.44) for the reaction energy on the one-hot encoding of the element in α-position to the silicon with a 70:30 random train-test split. LOO CV: Leave-One-Out cross-validation. k-fold CV: 200 times repeated 4-fold cross-validation. LOSO CV: Leave-One-Substituent-Out cross-validation. Increments are calculated from the coefficients for every feature (e.g. element in α-position) of the linear model.

3.4 Bag of Elements: ΔG_R for germylenes and increments for $\Delta G^‡$

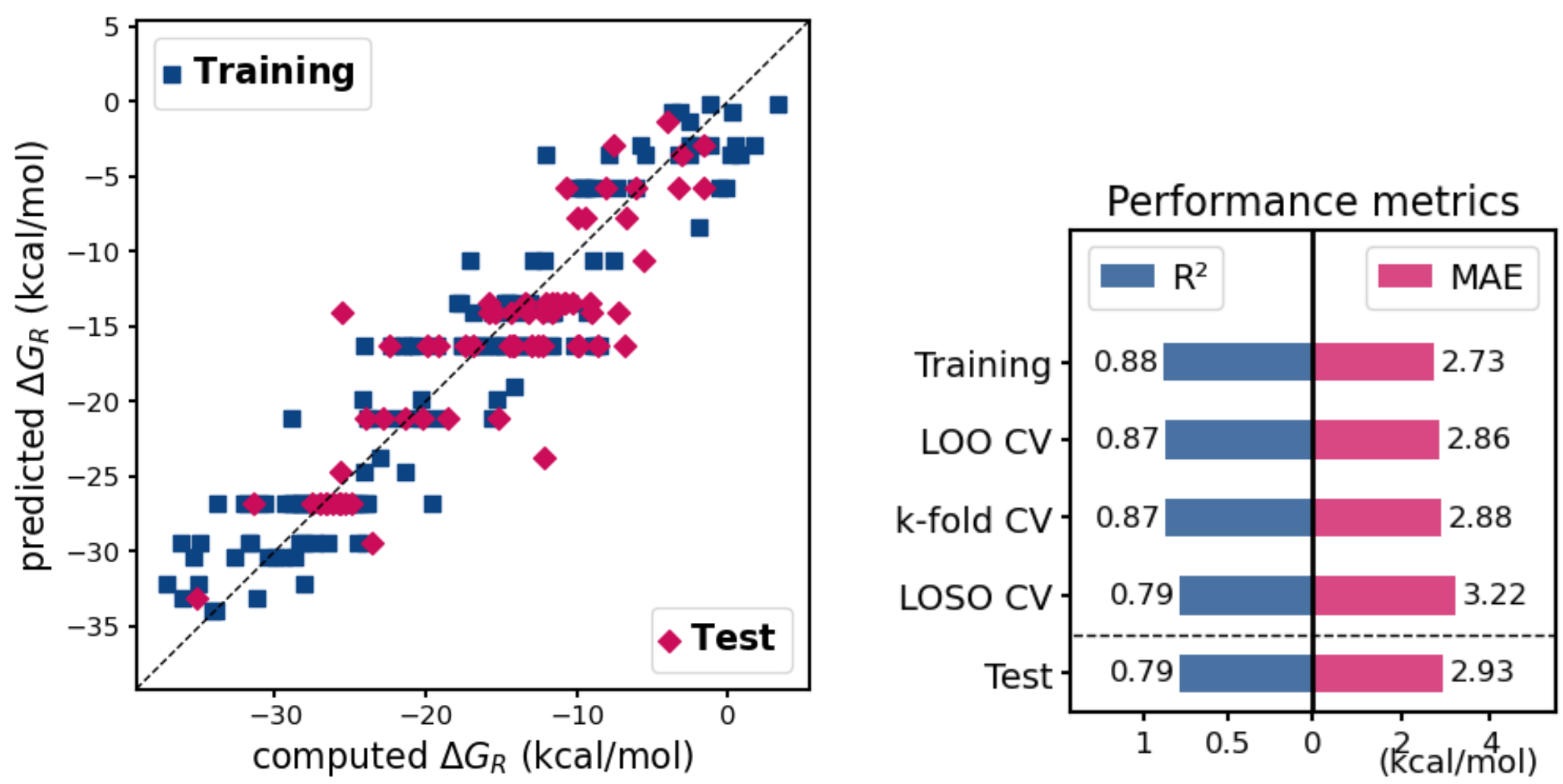

Figure S17: Bag of Elements (BoE) model with a linear Ridge regression (α = 1.44) for the reaction energy on the one-hot encoding of the element in α-position to the germanium with a 70:30 random train-test split. LOO CV: Leave-One-Out cross-validation. k-fold CV: 200 times repeated 4-fold cross-validation. LOSO CV: Leave-One-Substituent-Out cross-validation.

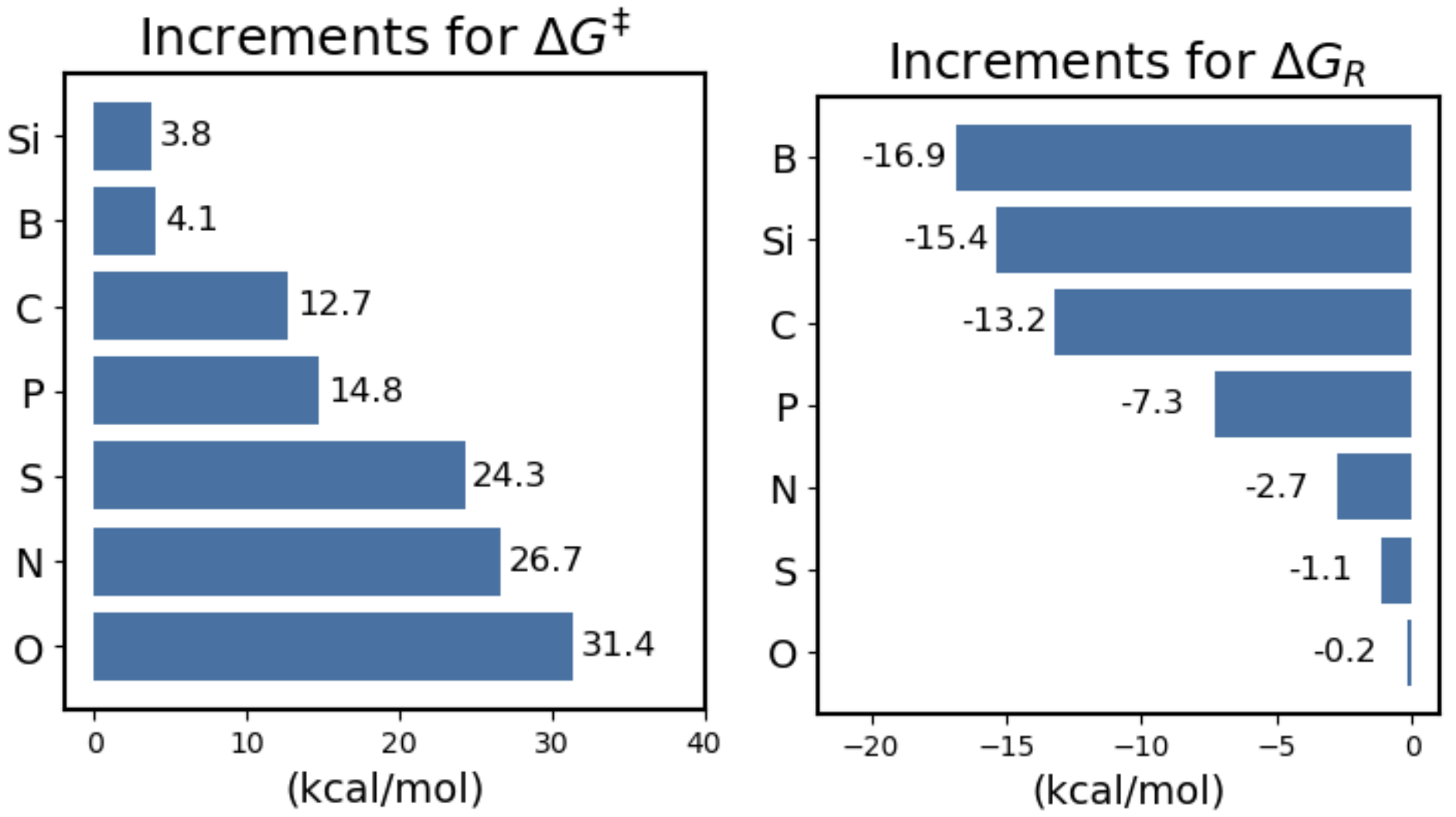

Figure S18: Increments for the (left) barrier and (right) energy of the H_2 activation with germylenes obtained from the coefficients for every feature (e.g. element in α-position) of the linear Bag of Elements model (train-test split: 70:30; α = 1.44).

3.5 Bag of Elements: *ΔG$_R$* for H_2O activation with silylenes and increments for *ΔG‡*

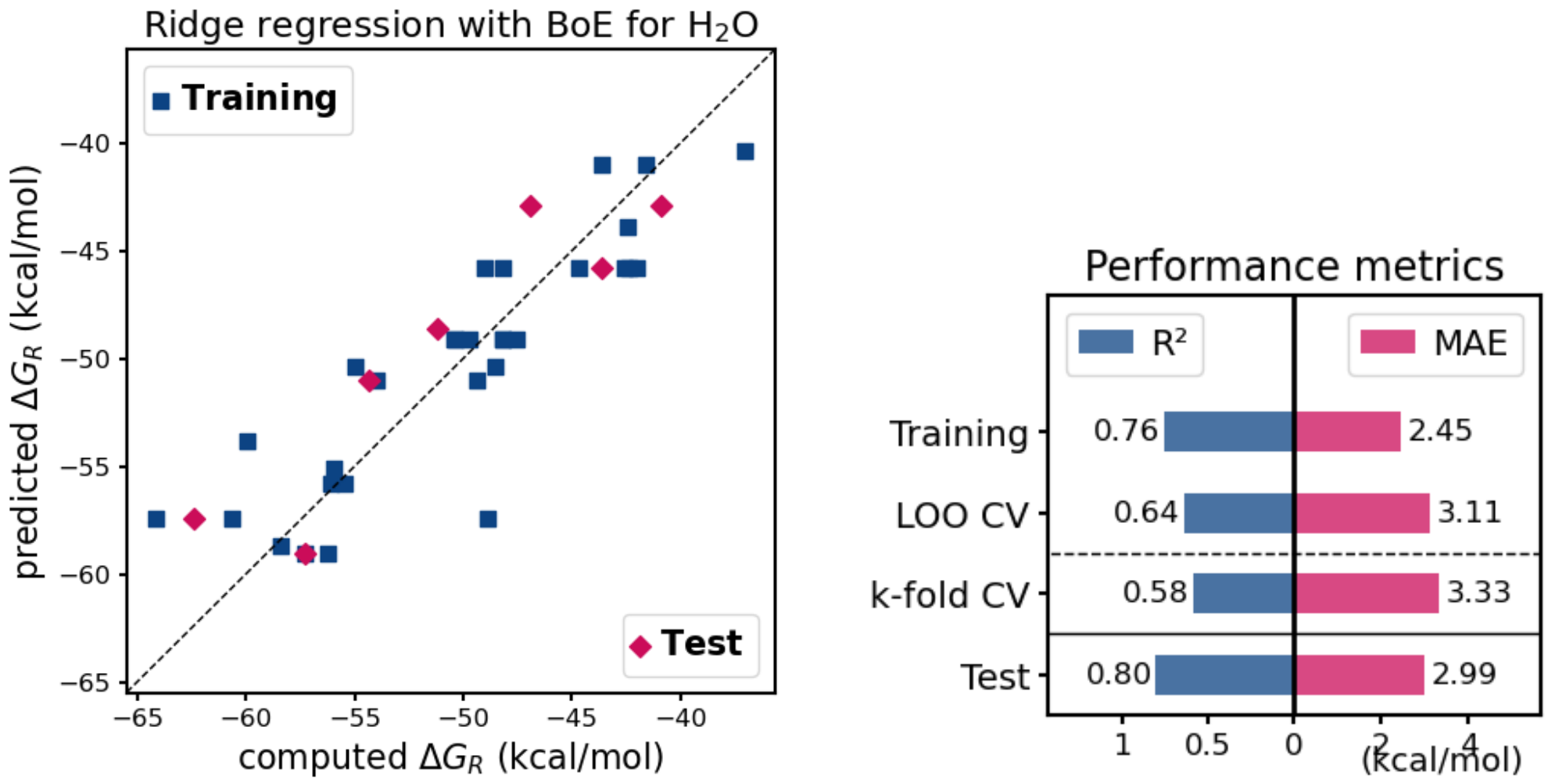

Figure S19: Bag of Elements (BoE) model with a linear Ridge regression (α = 1.44) for the reaction energy of H_2O activation on the one-hot encoding of the element in α-position to the silicon with a 70:30 equidist train-test split. LOO CV: Leave-One-Out cross-validation. k-fold CV: 200 times repeated 4-fold cross-validation. LOSO CV: Leave-One-Substituent-Out cross-validation.

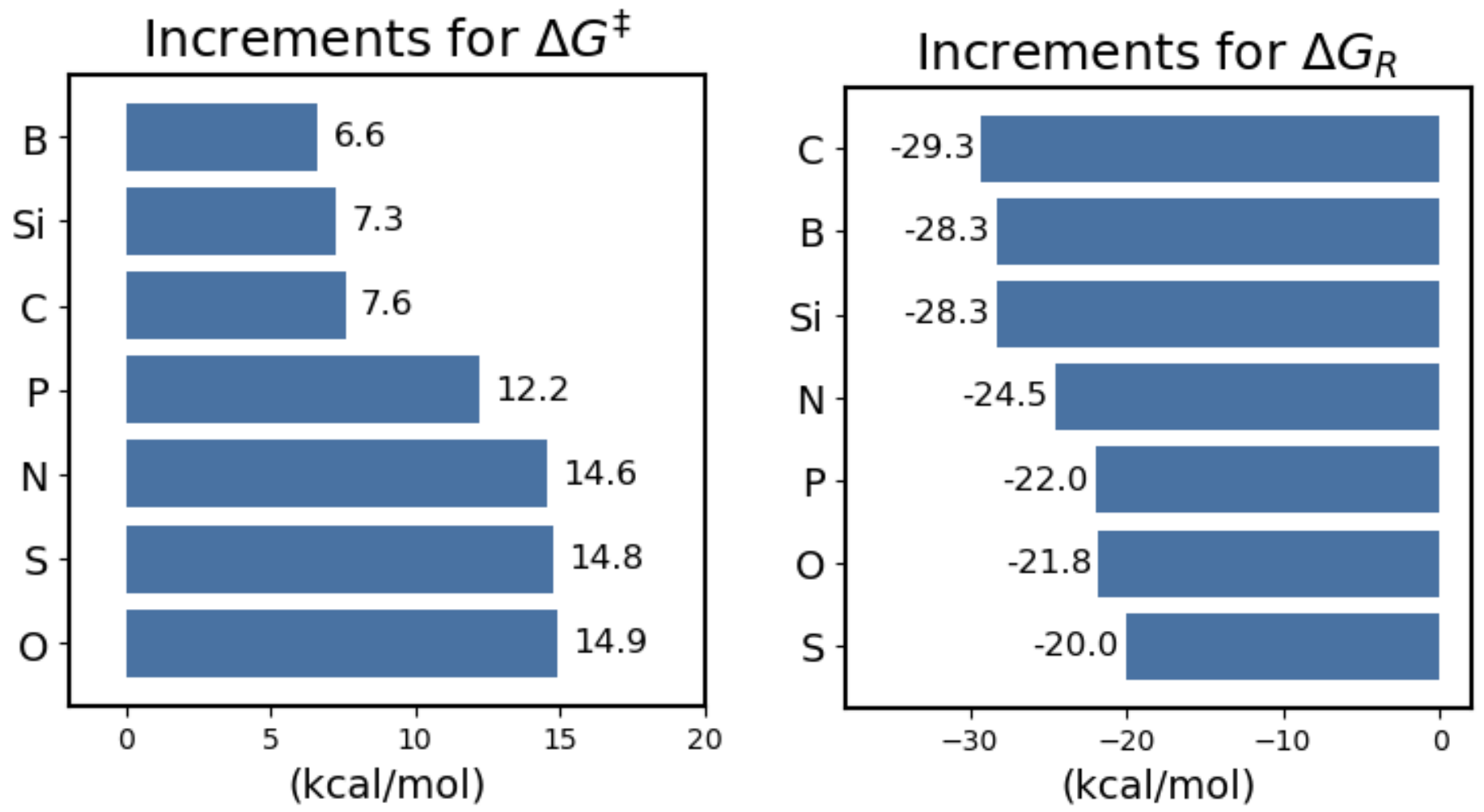

Figure S20: Increments for the (left) barrier and (right) energy of H_2O activation with silylenes obtained from the coefficients for every feature (e.g. element in α-position) of the linear Bag of Elements model (train-test split: 70:30; α = 1.44).

3.6 Linear correlation of energies of silylenes and germylenes

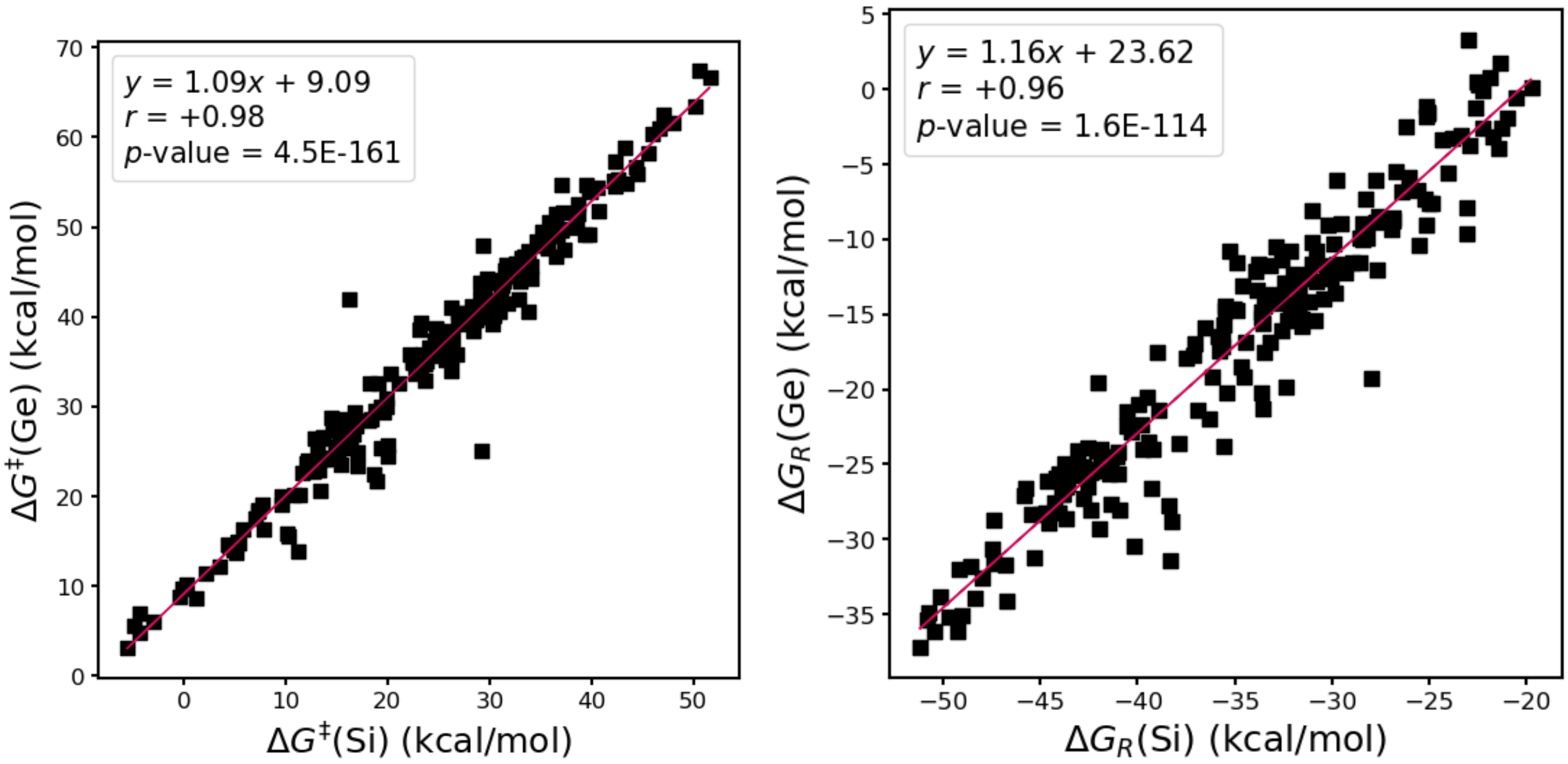

Figure S21: Linear correlation of the activation barriers (left) and reaction energies (right) between silylene and germylene analogues.

3.7 Linear and Non-linear univariate correlations

Pearson correlation analysis was used to investigate how steric and electronic descriptors correlate to the target variable. Since Pearson correlation is unable to detect non-linear correlations, we also tested non-linear univariate correlations to further support conclusions made based on the Pearson correlations.

Table S4 compares the 20 best univariate linear and non-linear correlations of our DFT-level descriptors with the barrier of H_2 activation by silylenes, sorted by their R^2 value. The values for the non-linear correlations demonstrate, that there are no correlations better than the linear ones and that Hardness, Softness and HOMO-LUMO gap are the features that are most correlated with the target. Furthermore, the R^2 values drop down significantly faster for the non-linear correlations, showing that there are fewer meaningful correlations.

Table S5: Comparison of the 20 best linear and non-linear correlations, sorted by the respective R^2 value.

**Linear correlations** $y = mx + b$		**Non-linear correlations** $y = a^{mx} + b$	
Feature	*R²*	*Feature*	*R²*
Hardness	0.67	Hardness	0.67
Softness	0.64	Softness	0.67
HOMO_LUMO_gap	0.64	HOMO_LUMO_gap	0.64
s- @E	0.52	EL_dist	0.50
Charge @E	0.47	EL_dist_min	0.49
Charge @L_1	0.46	Charge @E	0.47
EL_dist	0.44	EA	0.42
Charge @L_1_max	0.44	EL_dist_max	0.39
LUMO	0.43	s0 @E	0.38
Electrophilicity	0.43	EN	0.36
EA	0.42	EL BO_min	0.29
Energy_HOMO(N+1)	0.40	EL BO	0.23
EL_dist_min	0.38	IP	0.15
s0 @E	0.37	EL BO_max	0.14
E_LP_s_char	0.34	Electrophilicity @E	0.09
EL_dist_max	0.32	qvtot_max_delta	0.04
EL BO_min	0.26	s- @L_1	0.04
Charge @L_1_min	0.25	ratio_vbur_vtot	0.03
CDD @E	0.24	ovtot_min	0.03
E_LP_occ	0.23	s- @L_1_max	0.03

3.8 Multiple linear regression: Feature selection

The model search was performed by an algorithm based on forward stepwise selection keeping a set of candidates[22]. The algorithm starts from all single-feature models and systematically adds features, ensuring that no pair of features within a model has squared correlation $R^2 \geq 0.5$. The algorithm starts from all single-feature models and systematically adds features, ensuring that no pair of features within a model has squared correlation $R^2 \geq 0.5$. At each step, we generate all single-feature expansions of the retained models, rank candidates by an initial screen, keep the top K, and compute their cross-validation metrics. The top N by this metric advance to the next step. This algorithm is supposed to generate the best MLR models for every number of parameters. If given 5 steps, the algorithm will generate the best MLR models with 1, 2, 3, 4 and 5 features.
We then calculated the R^2 score and the mean absolute error (MAE) values of the test set for all derived models, and filtered out overfits, defined by a too large deviation of the R^2 and MAE of the test set from that of the training set. The remaining models then were explored, searching for the best combination of statistical performance parameters and chemical interpretability while minimizing the number of features needed for the model. Individual MLR models then were explored in more detail by evaluating feature distributions, collinearity of the features, outliers, etc.

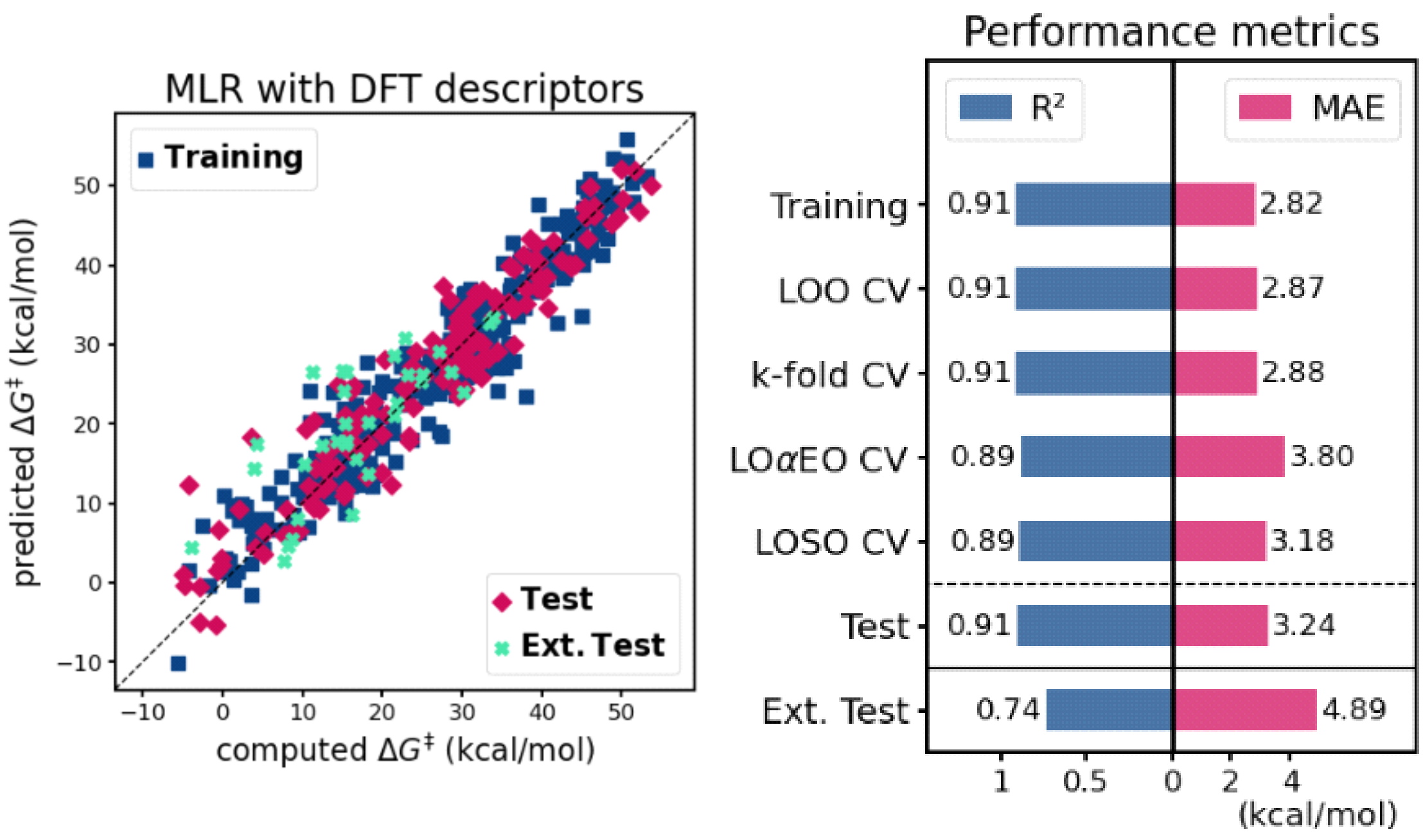

Figure S22: Multiple linear regression (MLR) model on DFT-level descriptors with a 70:30 random train-test-split. LOO CV: Leave-One-Out cross-validation. k-fold CV: 200 times repeated 4-fold cross-validation. LOαEO CV: Leave-One-α-Element-Out cross-validation. LOSO CV: Leave-One-Substituent-Out cross-validation. Feature selection is based on a stepwise algorithm based on internal cross-validation and linear independence of the features

$$\Delta G^{\ddagger}_{pred} = 25.83 - 6.06\ \boldsymbol{\Sigma d}(\text{Si–Subst.}) - 5.15\ \boldsymbol{f^{-}}(\text{Si}) + 4.96\ \boldsymbol{\eta} + 3.43\ \boldsymbol{\Sigma f^{-}}(\text{Subst.}) + 2.01\ \boldsymbol{LP_{occ}}$$

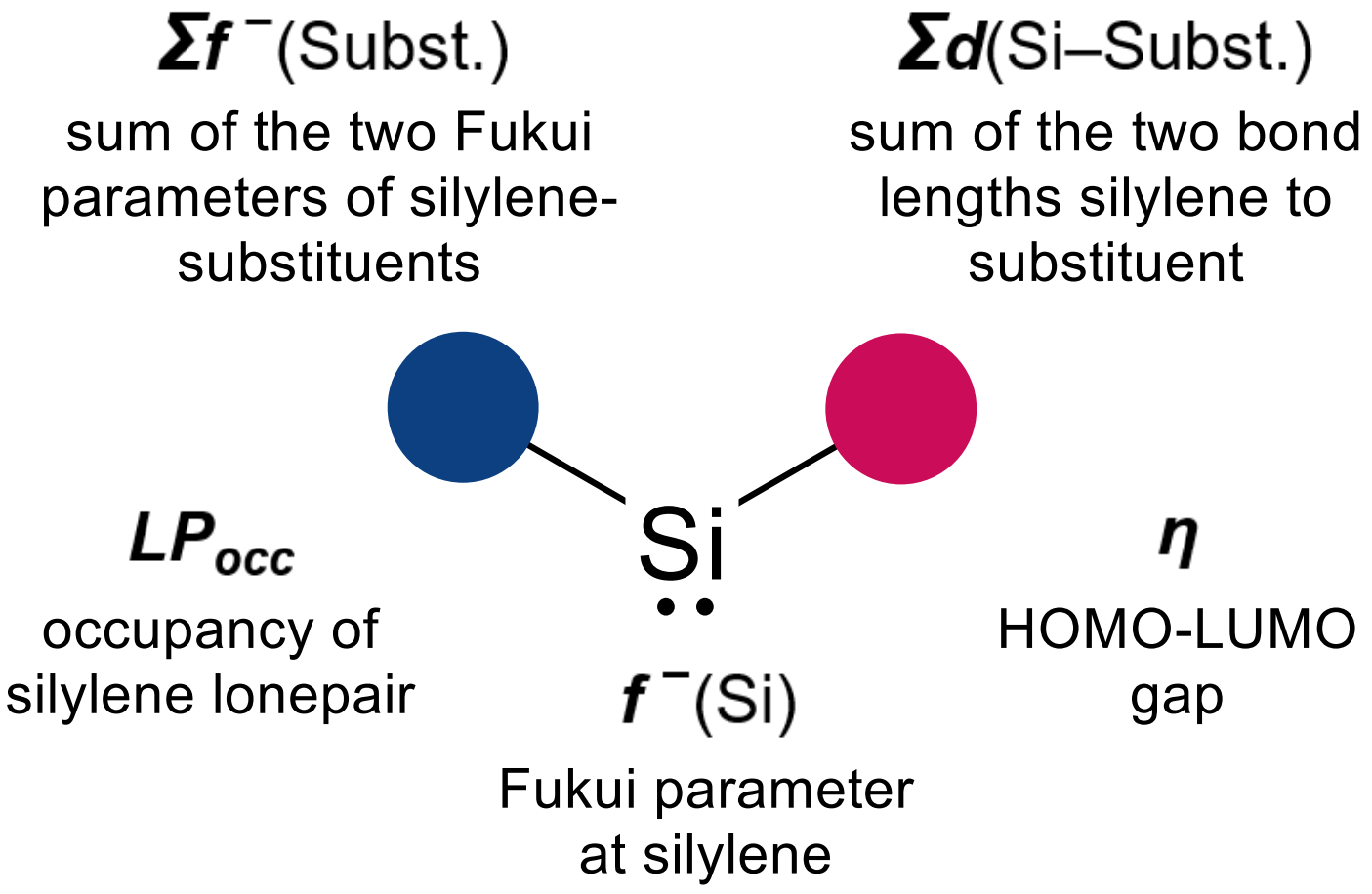

Figure S23: Features in the obtained MLR model.

4 Experimental Studies

4.1 General information

Chemical and Conditions

If not stated otherwise, all experiments were carried out using standard Schlenk techniques under an argon atmosphere, which was dry and free of oxygen. Argon (99.999%) was a product of *Air Liquide* and was used without any further drying. Involved solvents were dried using an MBraun SPS-800 (THF, toluene, diethyl ether, *n*-hexane and *n*-pentane) and stored over molecular sieves. Deuterated solvents were degassed and stored over molecular sieves in an argon-filled glovebox prior to use. Reagents were purchased from Sigma-Aldrich, ABCR, Acros Organics or TCI Chemicals and used without further purification if not stated otherwise. Germylenes **1**,[23] $Ar^{Mes}GeCl$,[23] and $Ge_2Br_2Tbb_2$,[24] and the phosphides Mes_2PNa [25] and $Dipp_2PNa$[25] was were prepared according to literature procedures.

Caution! Strong bases such as organopotassium bases, especially as neat compounds, are severely air-/moisture-sensitive and pyrophoric organometallic compounds. These compounds need to be handled under an inert gas atmosphere to exclude reactions with oxygen and water. Guidelines for their handling can be found in literature: T. L. Rathman, J. A. Schwindeman, *Org. Process Res. Dev.* **2014**, *18*, 1192.

Analytical methods

NMR Spectroscopy. ^{1}H, $^{13}C\{^{1}H\}$, $^{31}P\{^{1}H\}$ NMR spectra were recorded on Avance-III-500 or Avance-III-400 spectrometers at 25 °C if not stated otherwise. All values of the chemical shift are in ppm regarding the δ-scale. All spin-spin coupling constants (*J*) are printed in Hertz (Hz). To display multiplicities and signal forms correctly the following abbreviations were used: s = singlet, d = doublet, t = triplet m = multiplet, dd = doublet of doublet, br = broad signal. Signal assignment was supported by, HSQC (^{1}H / ^{13}C), HMBC (^{1}H / ^{13}C) correlation experiments. The full width at half maximum of broad signals was designated with $\Delta\nu_{1/2}$ and is given in Hz. All coupling constants are given in Hz as absolute values regardless of their signs. The measurement conditions for each spectrum are provided in the corresponding figures included in Chapter **4.2**.

HR-MS. High resolution mass spectrometry experiments were conducted in the in-house facility on a JEOL AccuTof GCV (JMS-T100GCV) in LIFDI mode. All samples were prepared with 1 mg of compound dissolved in 1 mL of toluene. Prior to measurement all samples were filtrated over cotton. The emitter heating current was set to 20 mA min-1 at a constant rate.

For details about the single-crystal Xray diffraction analyses, see chapter **5**.

4.2 Synthesis of germylenes

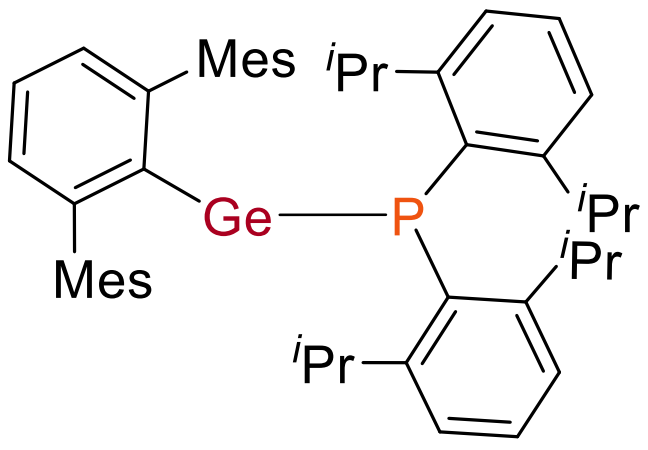

Mes = 2,4,6-Me_3-C_6H_2

2

To a pre-cooled, stirred pale-yellow solution of $Ar^{Mes}GeCl$ (60 mg, 0.142 mmol, 1.0 equiv.) in THF (2.0 mL), an orange solution of the phosphide $Dipp_2PNa$ (55 mg, 0.145 mmol, 1.02 equiv.) in THF (2.0 mL) was added dropwise. The reaction mixture immediately turned red-brown. It was slowly warmed to room temperature and stirred for an additional 1 h. Analysis of an aliquot by in situ ^{31}P NMR spectroscopy showed very selective formation of the product, along with a minor side product, $Dipp_2PH$ (~6 mol%). The reaction mixture was evaporated to dryness under reduced pressure, and the obtained brown residue was extracted with Et_2O (1.5 mL) at room temperature, whereupon an incipient crystallization started. The mother liquor was stored at −40 °C overnight. The resulting microcrystalline residue was separated by filtration using a filter cannula and dried under reduced pressure at room temperature for 15 min to give compound **2** as orange-brown solid (62 mg, 0.084 mmol, 59%).

^{1}H-NMR (400 MHz, THF-d_8, 298 K): δ = 7.35 (t, $^{3}J_{HH}$ = 7.6 Hz, 1H, C^4-*H*, Ar^{Mes}), 7.20-7.13 (m, 4H, $C^{3,5}$-*H* + C4-*H*, Dipp), 7.02 (d, $^{3}J_{HH}$ = 7.5 Hz, 2H, $C^{3,5}$-*H*, Ar^{Mes}), 6.92 (d, $^{3}J_{HH}$ = 7.2 Hz, 2H, $C^{3,5}$-*H*, Dipp), 6.89 (s, br, $\Delta\nu_{1/2}$ = 9.2 Hz, 2H, $C^{3/5}$-*H*, Mes), 6.73 (s, br, $\Delta\nu_{1/2}$ = 11.9 Hz, 2H, $C^{3/5}$-*H*, Mes), 3.95 (m, br, 2H, C*H*Me_2, Dipp), 2.96 (s, br, $\Delta\nu_{1/2}$ = 61.4 Hz, 2H, C*H*Me_2, Dipp), 2.28 (s, br, $\Delta\nu_{1/2}$ = 22.1 Hz, 6H, *Me*, Mes), 2.24 (s, 6H, *Me*, Mes), 1.82 (s, br, $\Delta\nu_{1/2}$ = 39.9 Hz, 6H, *Me*, Mes), 1.17 (d, $^{3}J_{HH}$ = 6.6 Hz, 6H, CHMe_2, Dipp), 1.0 (m, br, 12H, CHMe_2, Dipp), 0.22 (s, br, $\Delta\nu_{1/2}$ = 37.6 Hz, 6H, CHMe_2, Dipp) ppm.

^{31}P{^{1}H} NMR (162 MHz, THF-d_8, 298 K): δ = 67.2 ppm.

^{13}C{^{1}H}-NMR (100 MHz, THF-d_8, 298 K): δ = 159.32 (d, $^{2}J_{CP}$ = 23 Hz, C^1, Ar^{Mes}), 153.45 (d, very br, $C^{2,6}$, Dipp), 147.41 (s, br, $\Delta\nu_{1/2}$ = 26.5 Hz, $C^{2,6}$, Ar^{Mes}), 137.06 (s, br, $\Delta\nu_{1/2}$ = 11.6 Hz, Dipp), 130.32 (s, Dipp), 130.13 (s, br, $\Delta\nu_{1/2}$ = 16.9 Hz, $C^{3/5}$, Mes), 129.41-129.34 (m, br, $C^{3,4,5}$, Ar^{Mes}), 129.15 (s, br, $\Delta\nu_{1/2}$ = 10.4 Hz, $C^{3/5}$, Mes), 125.98 (d, $^{3}J_{CP}$ = 7.5 Hz, $C^{3,5}$, Dipp), 124.49 (m, br, $C^{3,4,5}$, Dipp), 34.24 (d, $^{3}J_{CP}$ = 9.3 Hz, *C*HMe_2, Dipp), 24.67 (very br, CHMe_2, Dipp), 23.95 (s, br, $\Delta\nu_{1/2}$ = 7.8 Hz, CHMe_2, Dipp), 22.69 (s, br, $\Delta\nu_{1/2}$ = 18.8 Hz, *Me*, Mes), 21.68 (s, br, $\Delta\nu_{1/2}$ = 14.7 Hz, *Me*, Mes), 21.33 (s, *Me*, Mes) ppm.

**The full width of half maximum ($\Delta\nu_{1/2}$) of the very broad signals could not be exactly determined.*

***At 298 K, the hindered rotation of the C^1(Dipp)–P and Ge–C^{ipso}(Ar^{Mes}) bonds on the NMR timescale led to broadening of the NMR signals. Furthermore, few resonances were not observed, whereas few others could not be assigned as the characteristic cross-peaks obtained from 2D NMR experiments were too weak.*

****To resolve the broad NMR signals, we performed variable-temperature ^{1}H NMR measurements over the temperature range of 263–328 K. However, within this range, most of the signals did not sharpen. It suggests that significantly lower temperatures (approx. 193 K) or higher temperatures (above 373 K) would likely be required to fully resolve all of the signals. Unfortunately, both temperature regimes are beyond the operational limits of the NMR instrumentation available in our institute facility.*

HRMS-LIFDI (m/z): $[M]^+$ calcd for $C_{48}H_{59}GeP$, 740.3566; found: 740.3567.

*Compound **2** is an air-sensitive orange-brown crystalline solid. It is highly soluble in most aliphatic and aromatic solvents. Under strict exclusion of air, a sample solution of **2** in toluene-d_8 showed minute decomposition after being heated at 110 °C for 3 h.*

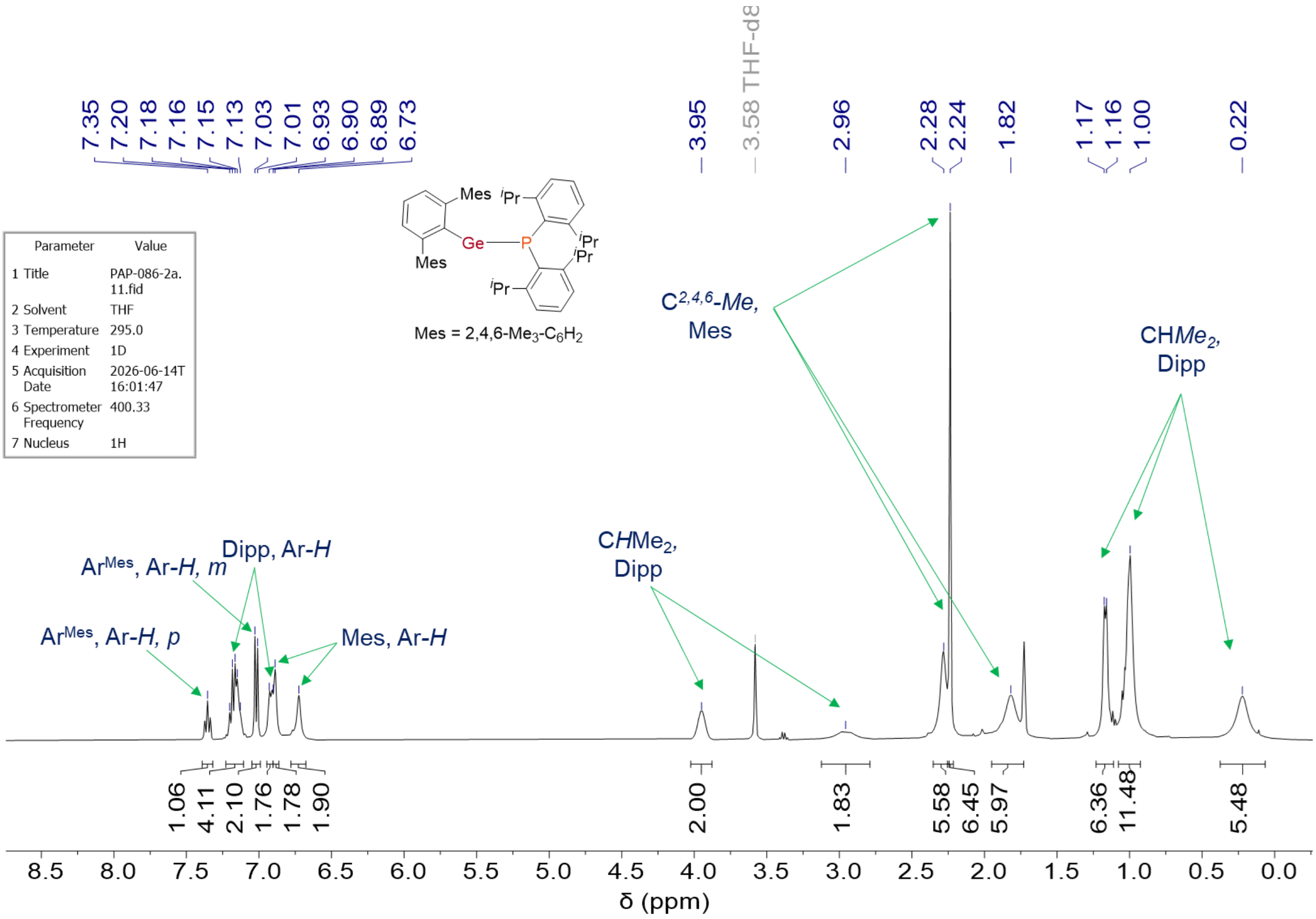

Figure S24. ^1H NMR spectrum of **2**.

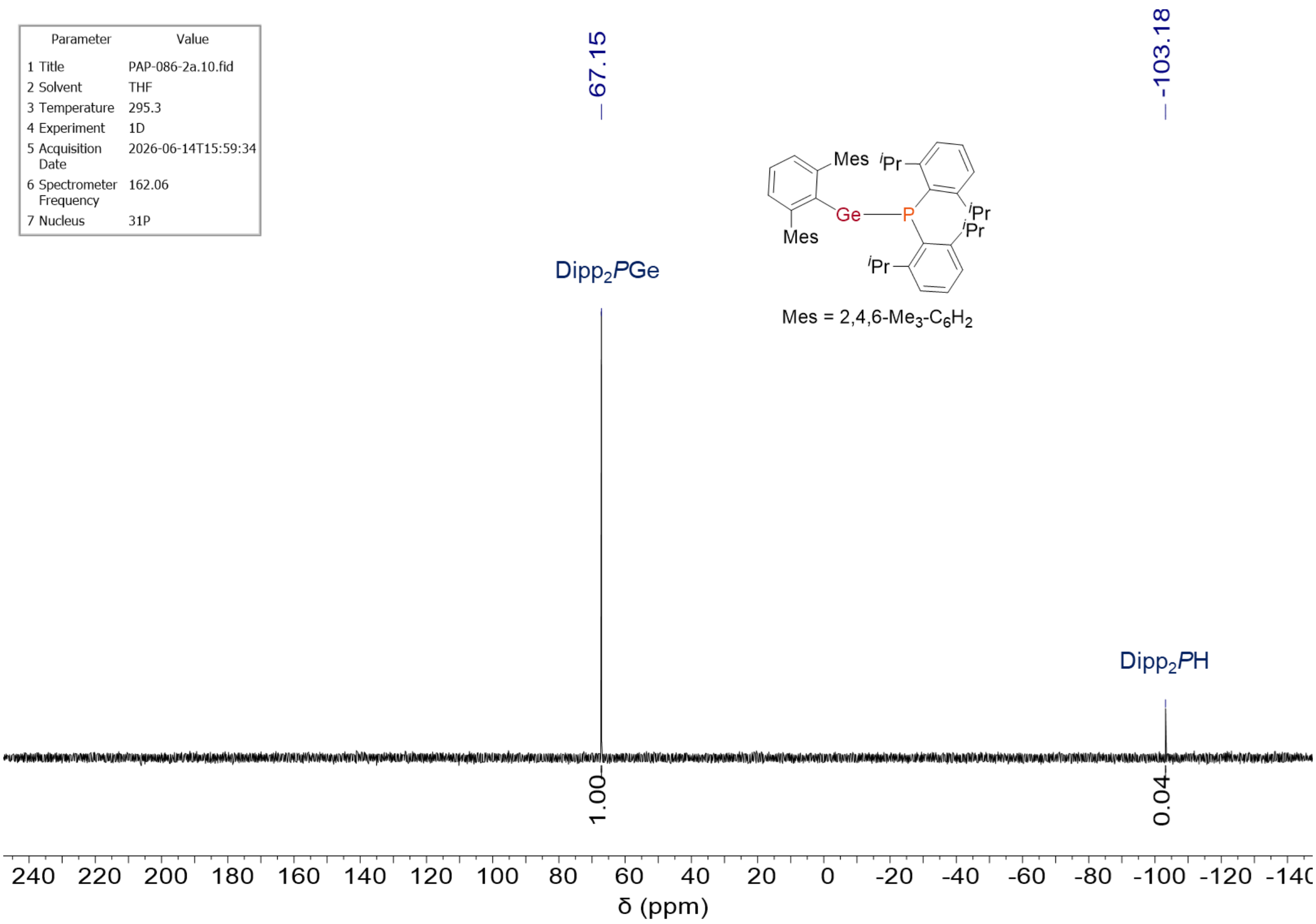

Figure S25. $^{31}P\{^1H\}$ NMR spectrum of **2**.

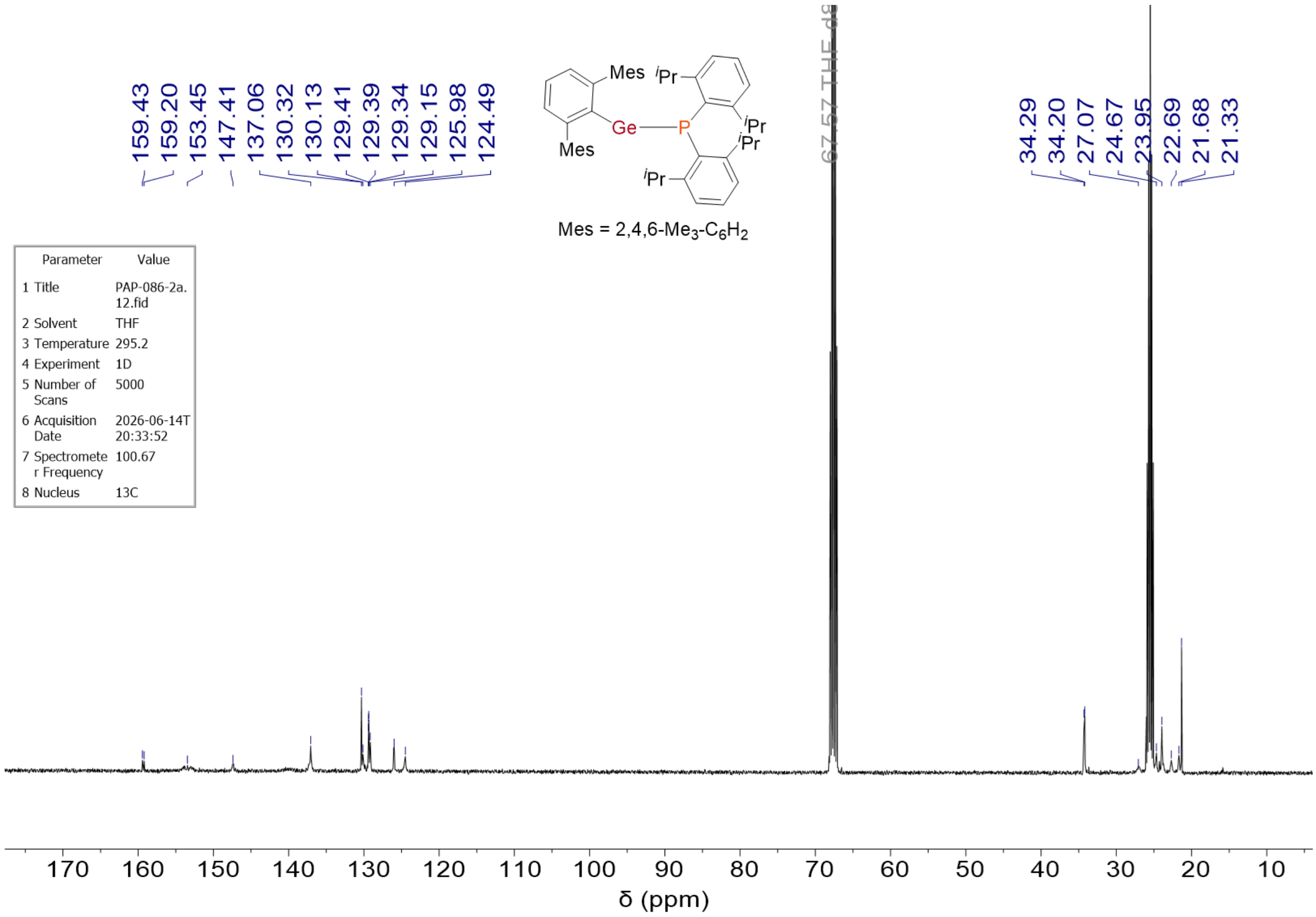

Figure S26. $^{13}C\{^1H\}$ NMR spectrum of **2**.

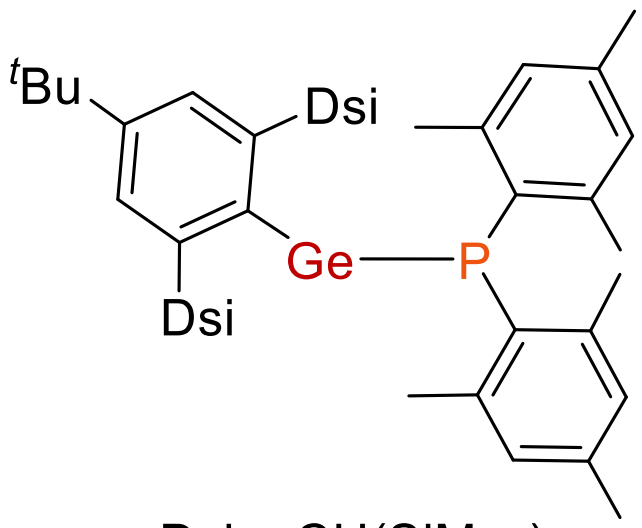

Dsi = $CH(SiMe_3)_2$

3

To a pre-cooled, stirred yellow solution of $Ge_2Br_2Tbb_2$ (80 mg, 0.066 mmol, 1.0 equiv.) in THF (2.0 mL), a dark-orange solution of the phosphide Mes_2PNa (40 mg, 0.14 mmol, 2.05 equiv.) in THF (1.5 mL) was added dropwise. The reaction mixture immediately turned orange-brown. It was slowly warmed to room temperature and stirred for an additional 1 h. Analysis of an aliquot by in situ $^{31}P\{^{1}H\}$ NMR spectroscopy showed very selective formation of the product, along with a minor side product, Mes_2PH (~10 mol%). The reaction mixture was evaporated to dryness under reduced pressure, and the obtained brown residue was extracted with Et_2O (1.3 mL) at room temperature, whereupon an incipient crystallization started. The mother liquor was stored at −40 °C overnight. The resulting microcrystalline residue was separated by filtration using a filter cannula and dried under reduced pressure at room temperature for 30 min to give compound **3** as maroon solid (74 mg, 0.093 mmol, 70%).

^{1}H-NMR (500 MHz, benzene-d$_6$): δ = 6.91 (s, 2H, $C^{3,5}$-*H*, Tbb), 6.72 (s, 4H, $C^{3,5}$-*H*, Mes), 2.54 (s, 12H, $C^{2,6}$-*Me*, Mes), 2.44 (s, 2H, C*H*$(SiMe_3)_2$, Tbb), 2.03 (s, 6H, C^4-*Me*, Mes), 1.36 (s, 9H, C*Me*$_3$, Tbb), 0.18 (s, 36H, Si*Me*$_3$, Tbb) ppm.

^{31}P{^{1}H} NMR (202 MHz, benzene-d$_6$): δ = 71.7 ppm.

^{13}C{^{1}H}-NMR (125 MHz, benzene-d$_6$): δ = 152.82 (d, $^{2}J_{CP}$ = 23.1 Hz, 1C, C^1, Tbb), 150.40 (s, 1C, C^4, Tbb), 147.15 (d, $^{3}J_{CP}$ = 8.2 Hz, 2C, $C^{2,6}$, Tbb), 142.48 (d, $^{2}J_{CP}$ = 5.4 Hz, 4C, $C^{2,6}$, Mes), 138.65 (s, 2C, C^4, Mes), 135.41 (br d, $^{1}J_{CP}$ = 5.09 Hz, 2C, C^1, Mes), 129.81 (d, $^{3}J_{CP}$ = 6.6 Hz, 4C, $C^{3,5}$, Mes), 122.49 (s, 2C, $C^{3,5}$, Tbb), 34.43 (s, 1C, *C*Me$_3$, Tbb), 32.14 (d, $^{4}J_{CP}$ = 6.8 Hz, 2C, *C*H$(SiMe_3)_2$, Tbb), 31.43 (s, 3C, C*Me*$_3$, Tbb), 25.06 (d, $^{3}J_{CP}$ = 7.2 Hz, 4C, $C^{2,6}$-*Me*, Mes), 21.01 (s, 2C, C^4-*Me*, Mes), 1.32 (s, 12C, Si*Me*$_3$, Tbb) ppm.

HRMS-LIFDI (m/z): $[M]^+$ calcd for $C_{42}H_{71}GeSi_4P$, 792.3582; found: 792.3607.

*Compound **3** is an air-sensitive maroon crystalline solid. It is highly soluble in most aliphatic and aromatic solvents. Under strict exclusion of air, a sample solution of **3** in toluene-d$_8$ showed almost no decomposition after being heated at 110 °C for 3 h.*

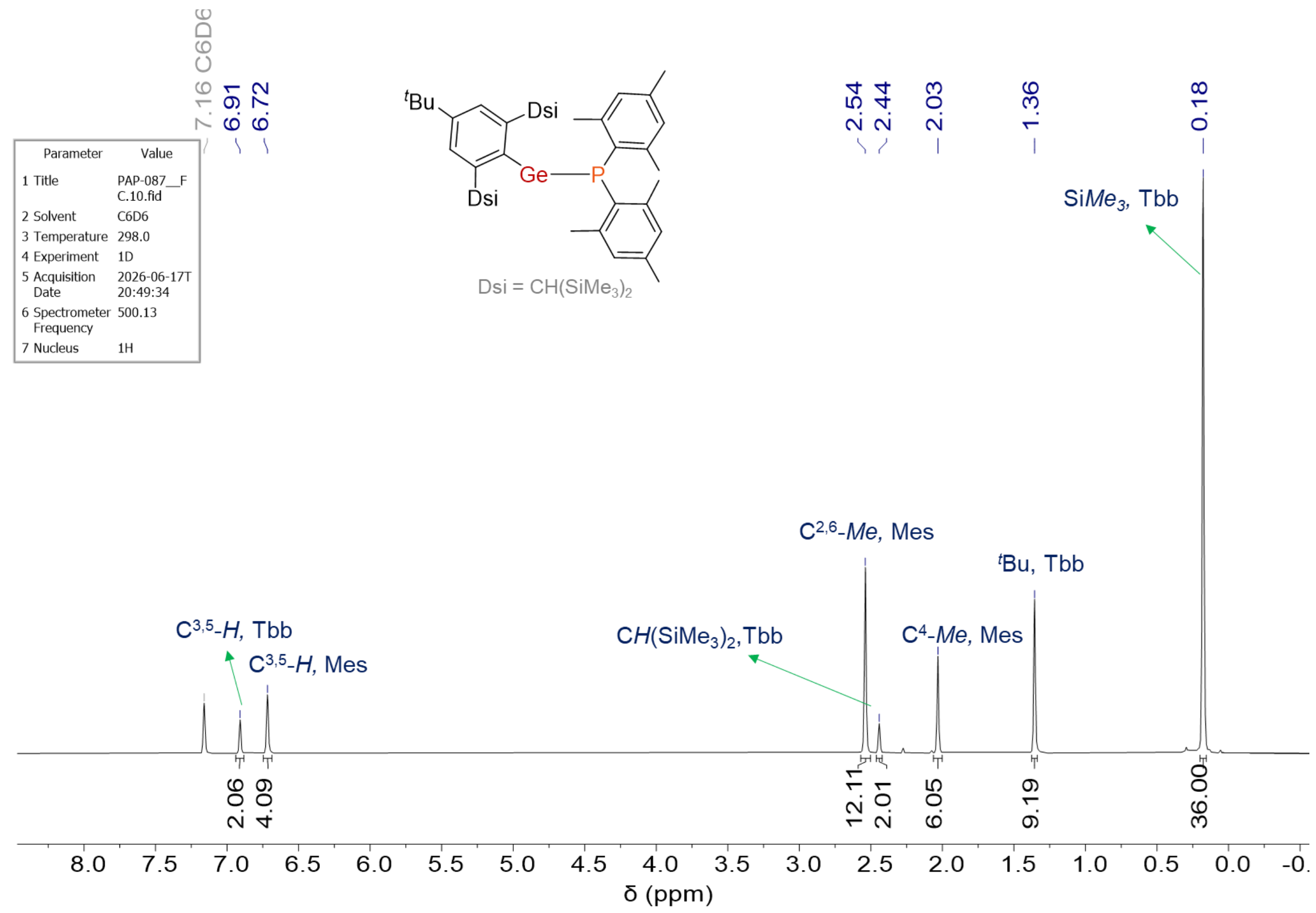

Figure S27. ^{1}H NMR spectrum of **3**.

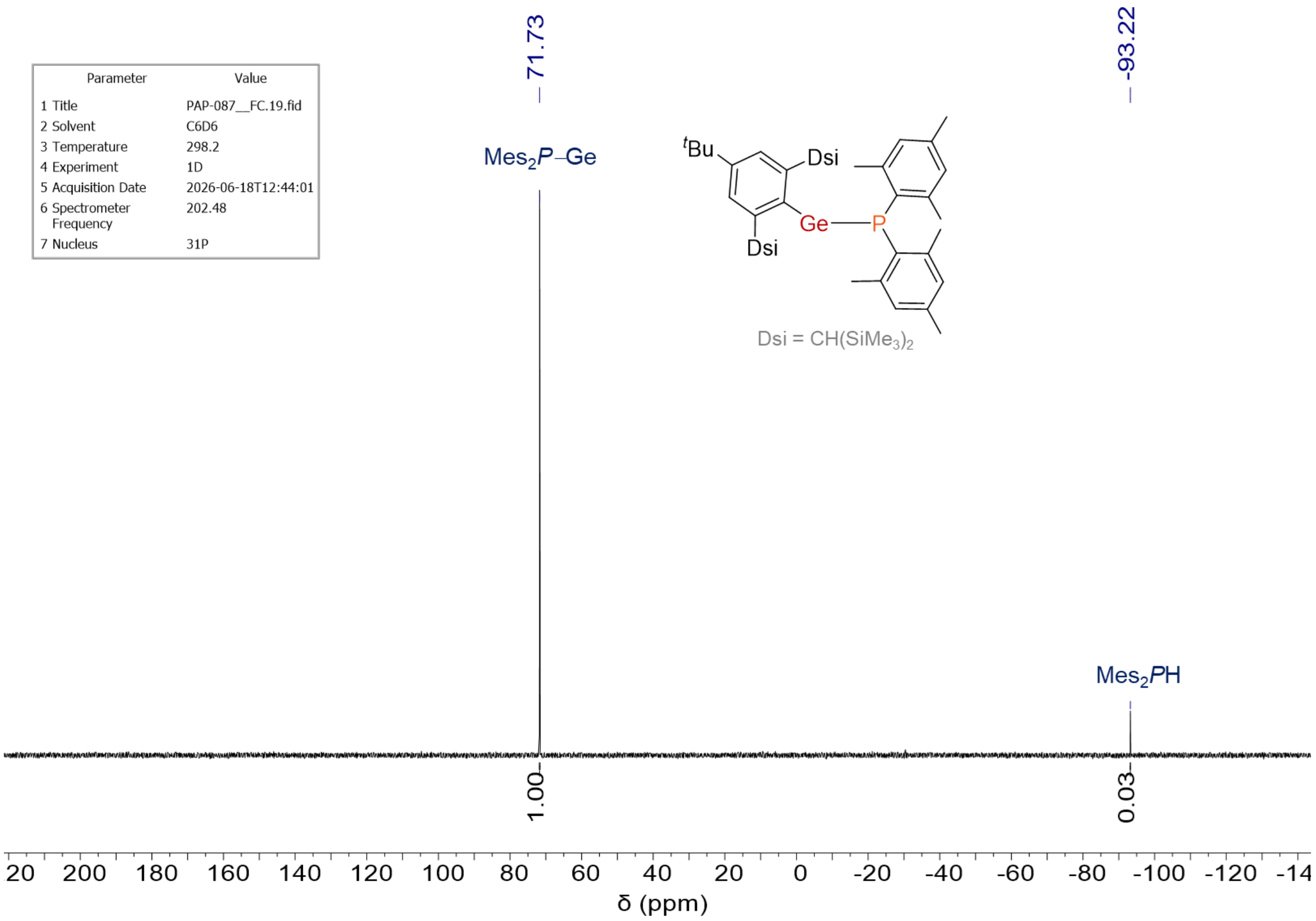

Figure S28. $^{31}P\{^{1}H\}$ NMR spectrum of **3**.

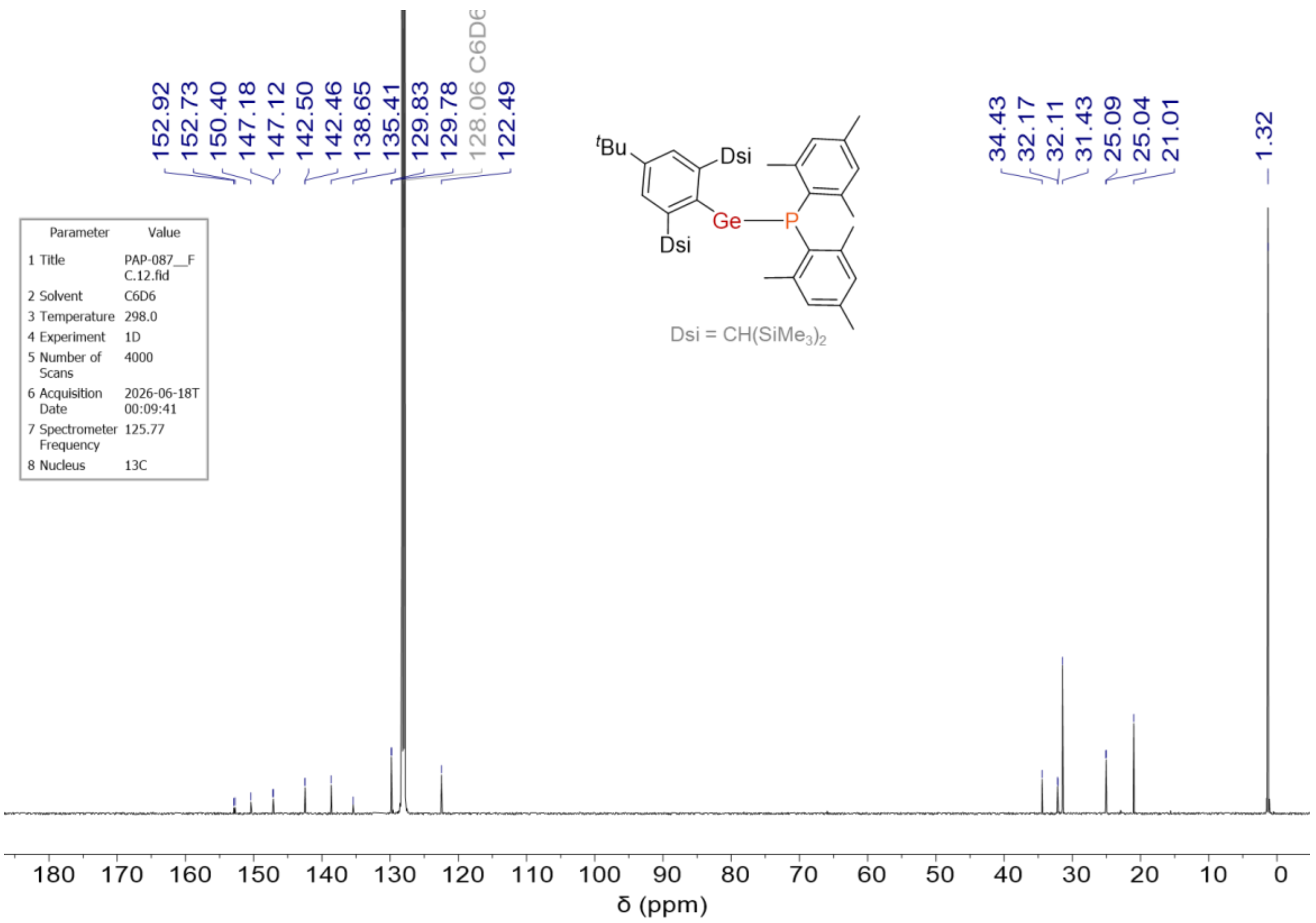

Figure S29. $^{13}C\{^1H\}$ NMR spectrum of **3**.

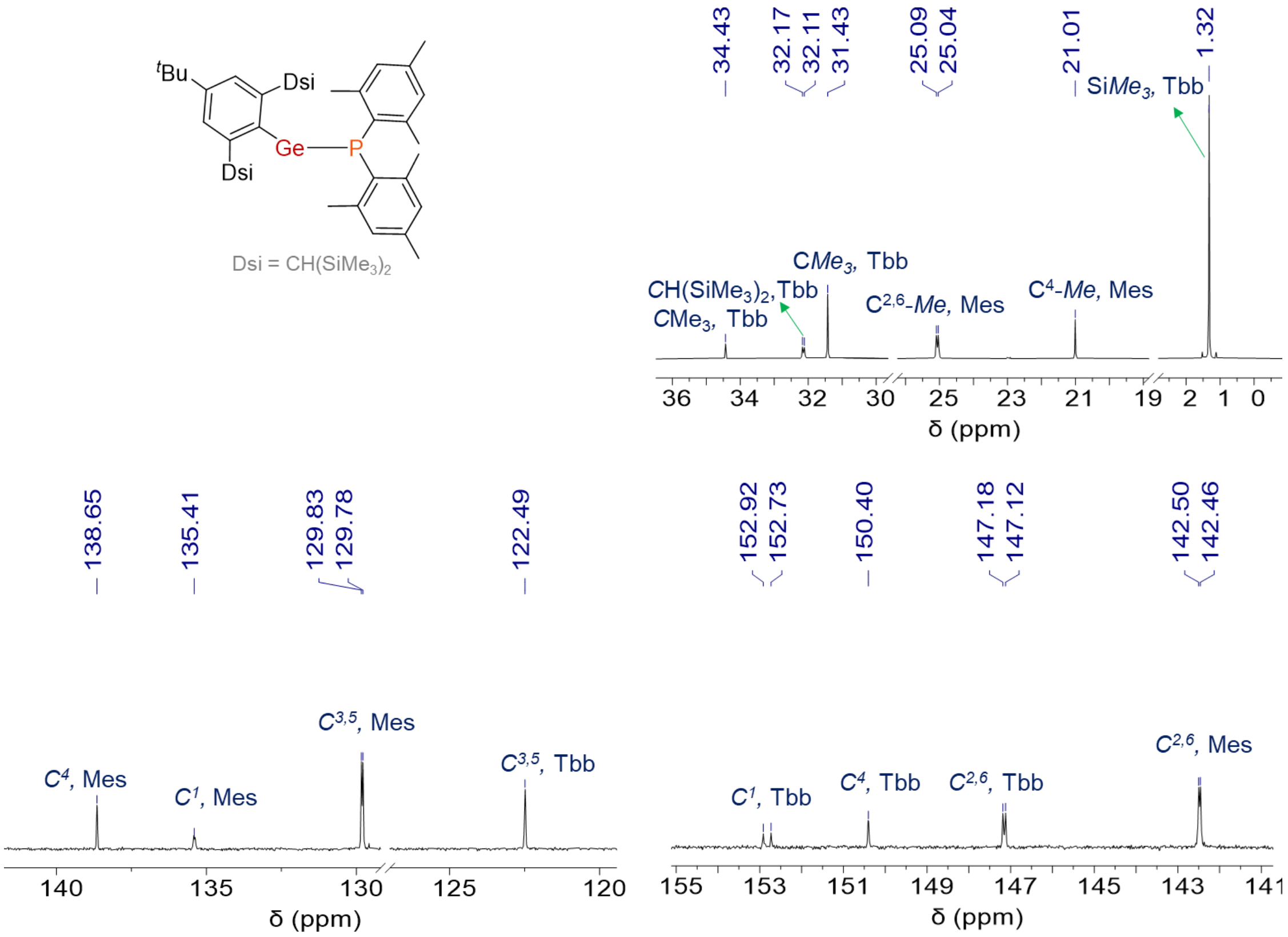

Figure S30. Selected excerpts of the $^{13}C\{^1H\}$ NMR spectrum of **3** depicted in **Figure S29**.

4.3 H_2 activation with diarylgermylene 1

To a solution of germylene (10 mg, 0.0143 mmol) and the internal standard 1,3,5-trimethoxybenzene (3.5 mg, 0.0207 mmol) in C_6D_6 (5.5 mL), an NMR tube was charged with H_2 gas (0.5 bar) at room temperature. The reaction progress was monitored by ^{1}H NMR spectroscopy over time. Kinetic measurements were performed at three different temperatures: 298, 303, and 313 K. Assuming complete conversion of the starting germylene to the dihydrogen activation product and first-order reaction kinetics, plots of [**P**] versus time and ln[**A**] versus time were generated. The latter plot was used to determine the rate constant (**k**) at each temperature. The rate constants obtained at the three temperatures were then used to construct an Eyring plot of ln(**k/T**) versus **1/T**, from which the activation parameters were determined.

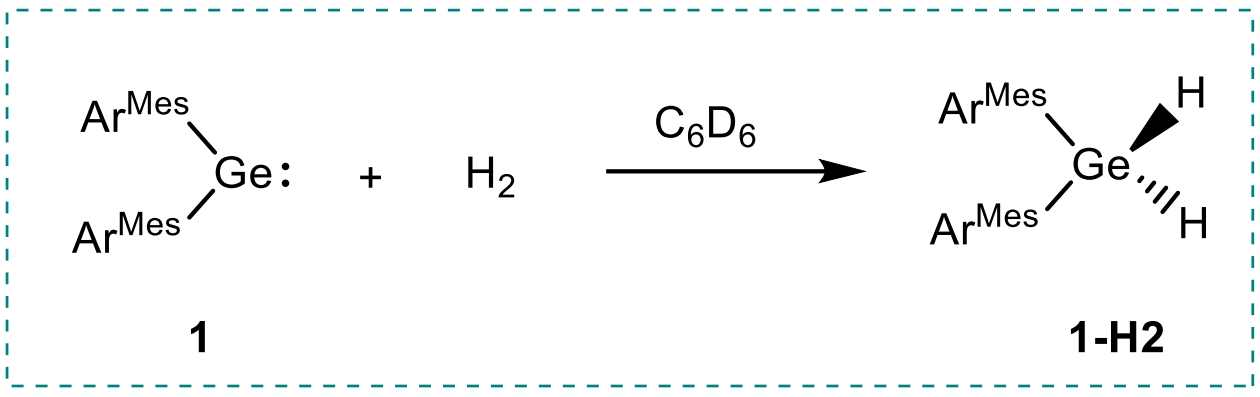

Scheme S2. General equation for H_2 activation by germylene **1**.

At **313 K**:

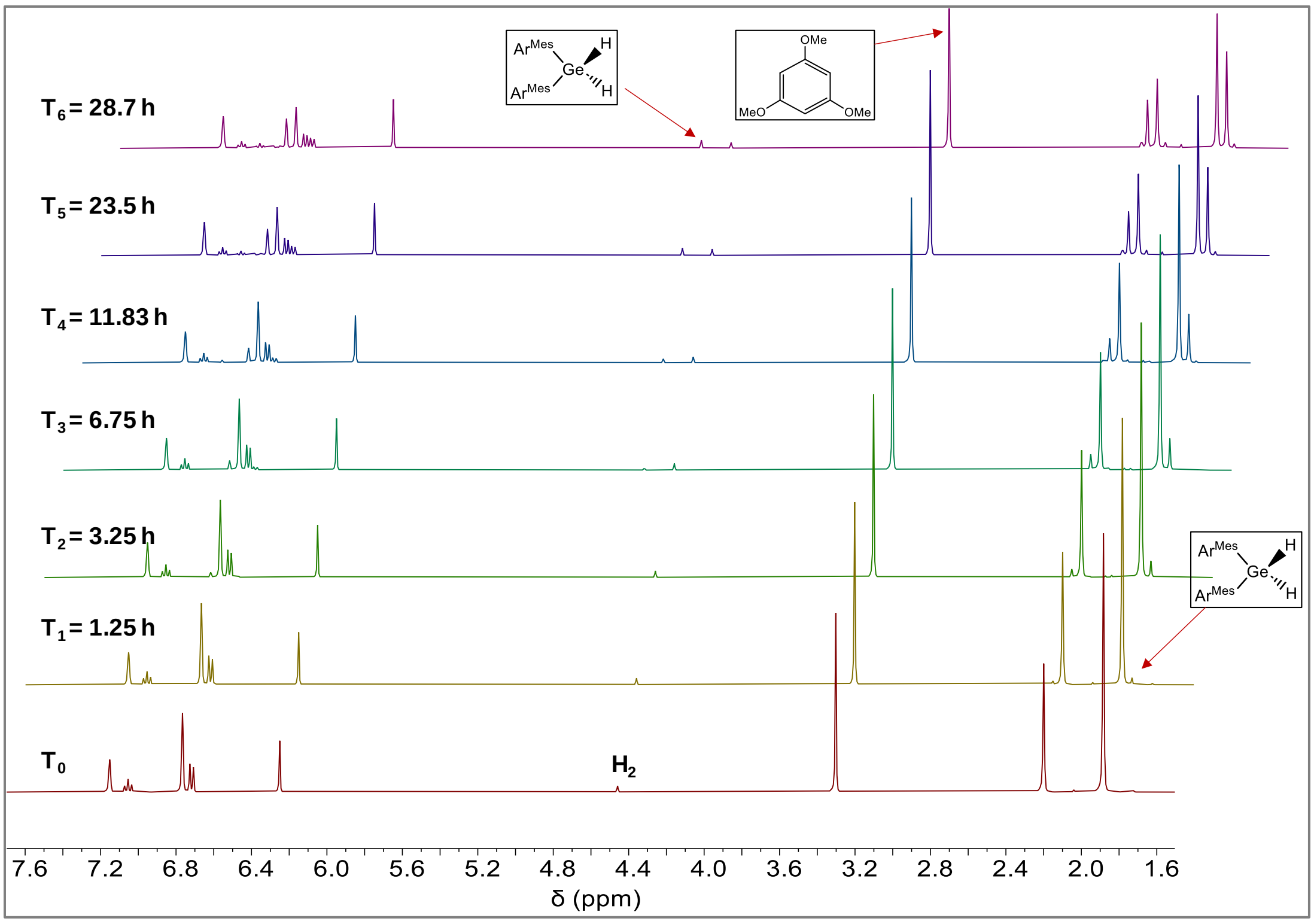

Figure S31. A stack plot of ^{1}**H NMR** spectra monitoring H_2 activation by germylene at 313 K.

Table S6. [1]**H NMR**-derived concentration profiles of the product and reactant at 313 K.

Time (hr)	Int. of P	[P]	[A]	ln[A]
0	0	0	0.026	-3.6497
1.25	0.61	0.6	0.02534	-3.6756
3.25	1.33	1.45	0.02455	-3.7070
6.75	2.62	2.86	0.02314	-3.7661
11.84	4.11	4.48	0.02152	-3.8385
23.5	7.16	7.8	0.0182	-4.0063
28.67	8.54	9.31	0.01669	-4.0920

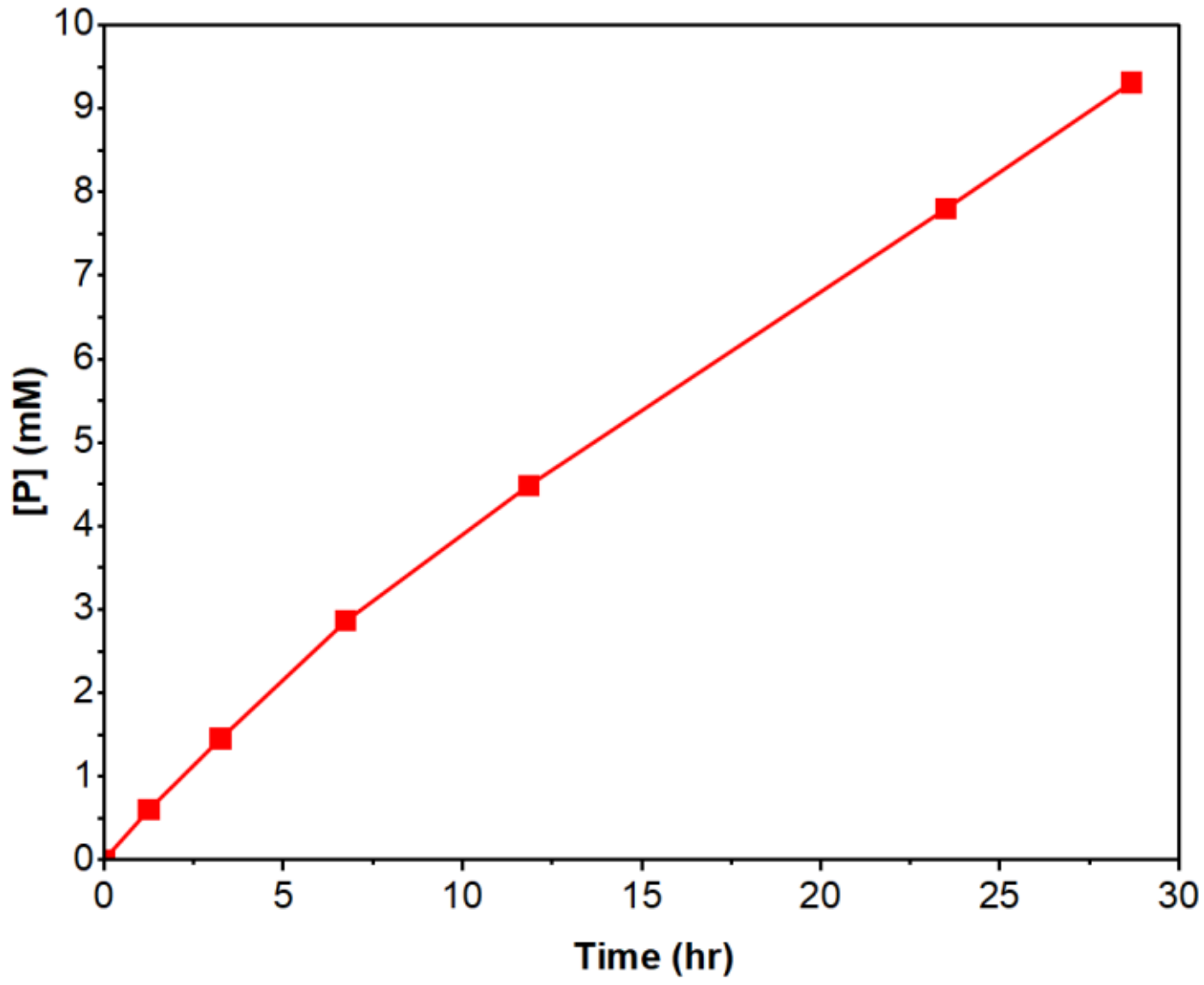

Figure S32. Plot of the conversion to the hydrogen-activated product over time at 313 K.

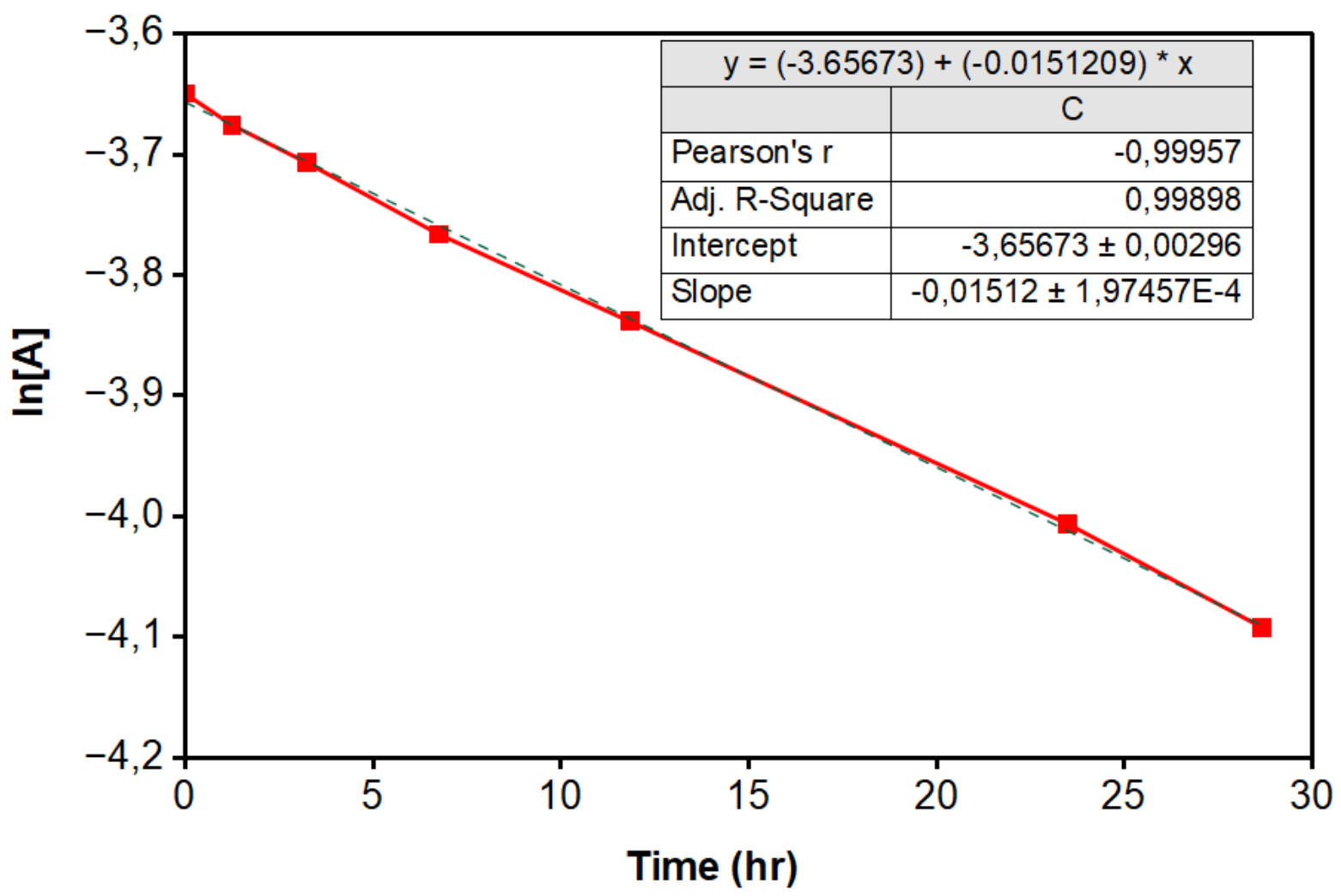

Figure S33. Plot of ln [A] vs time for H_2 activation at 313 K.

At **308 K**:-

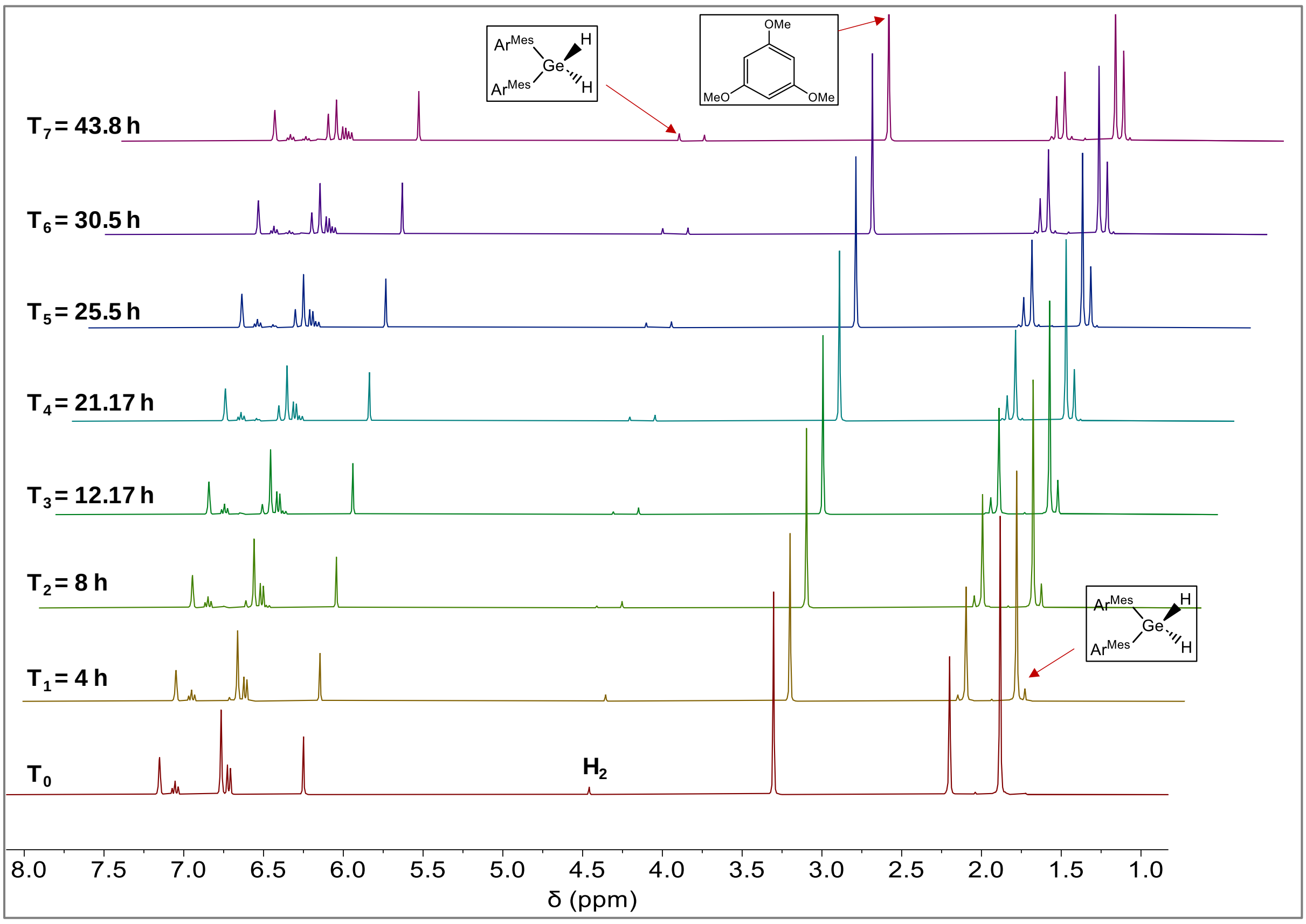

Figure S34. A stack plot of **¹H NMR** spectra monitoring H_2 activation by germylene at 308 K.

Table S7. ¹H NMR-derived concentration profiles of the product and reactant at 308 K.

Time (Sec)	Int. of P	[P]	[A]	ln[A]
0	0	0	0.026	-3.6497
4	1.12	1.22	0.02478	-3.6978
8	2.13	2.32	0.02368	-3.7432
12.17	2.99	3.26	0.02274	-3.7836
21.17	4.52	4.93	0.02107	-3.8598
25.5	5.21	5.68	0.02032	-3.8960
30.5	6.09	6.64	0.01936	-3.9446

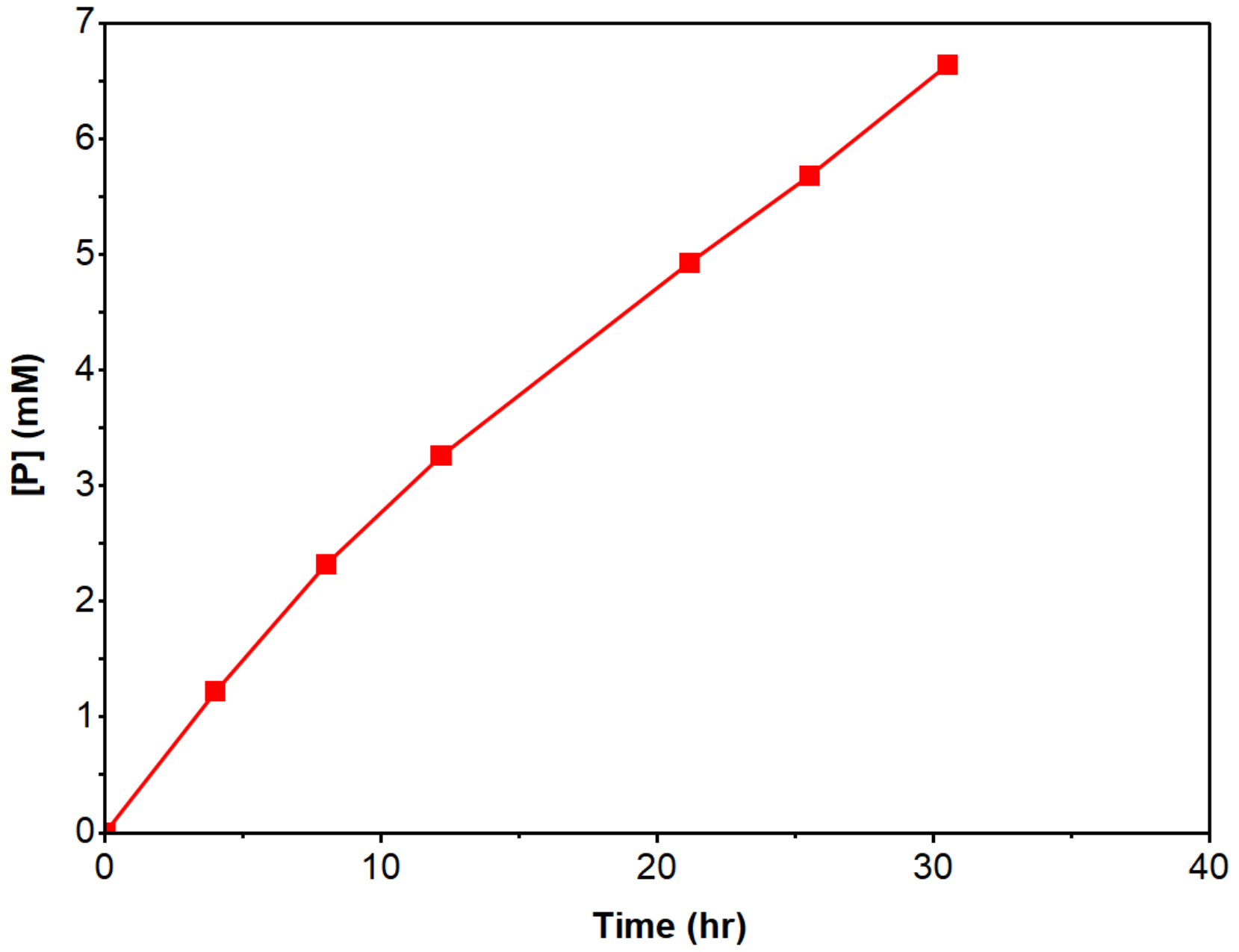

Figure S35. Plot of the conversion to the hydrogen-activated product over time at 308 K.

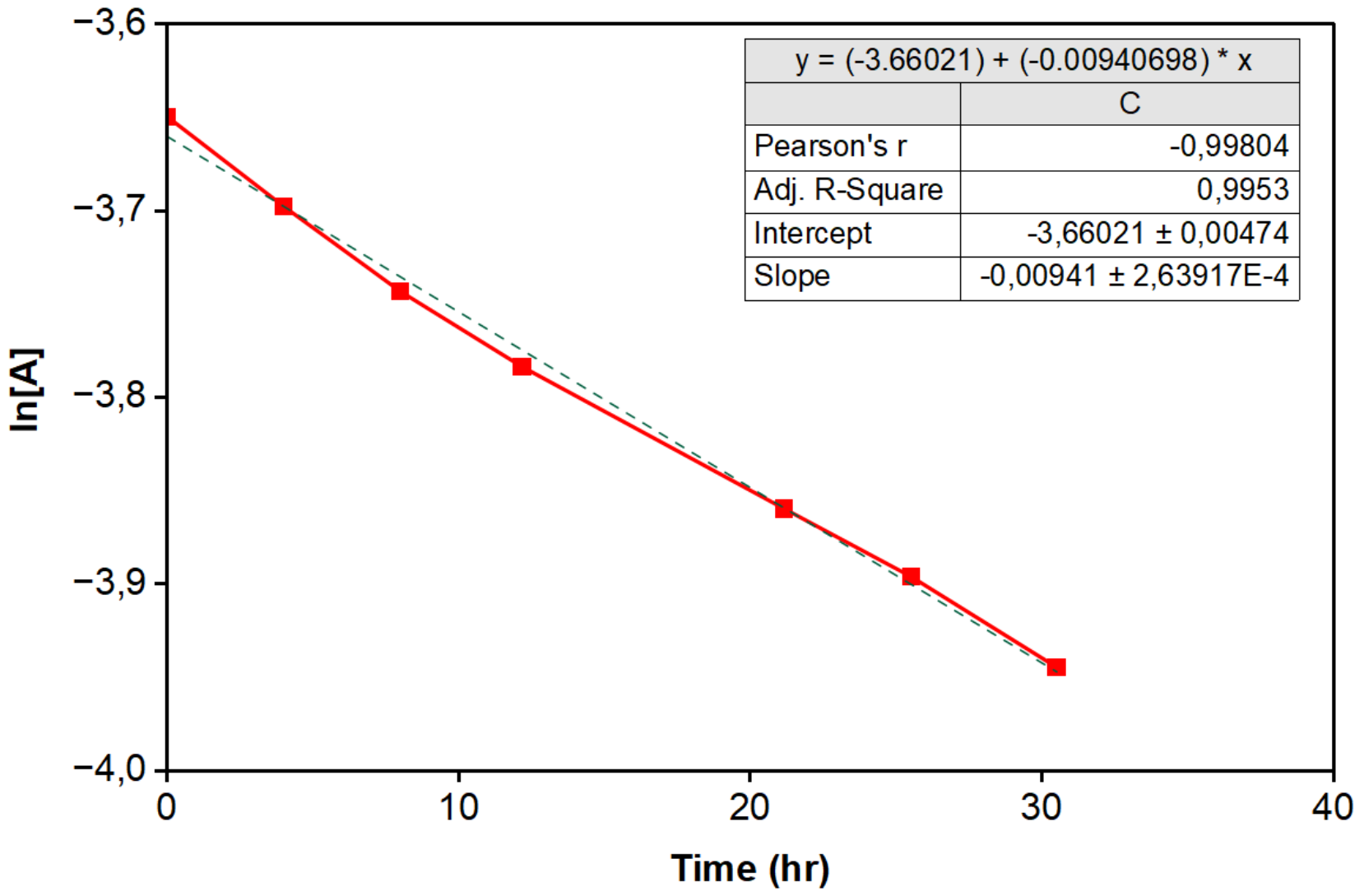

Figure S36. Plot of ln [A] vs time for H_2 activation at 308 K.

At **298 K**:-

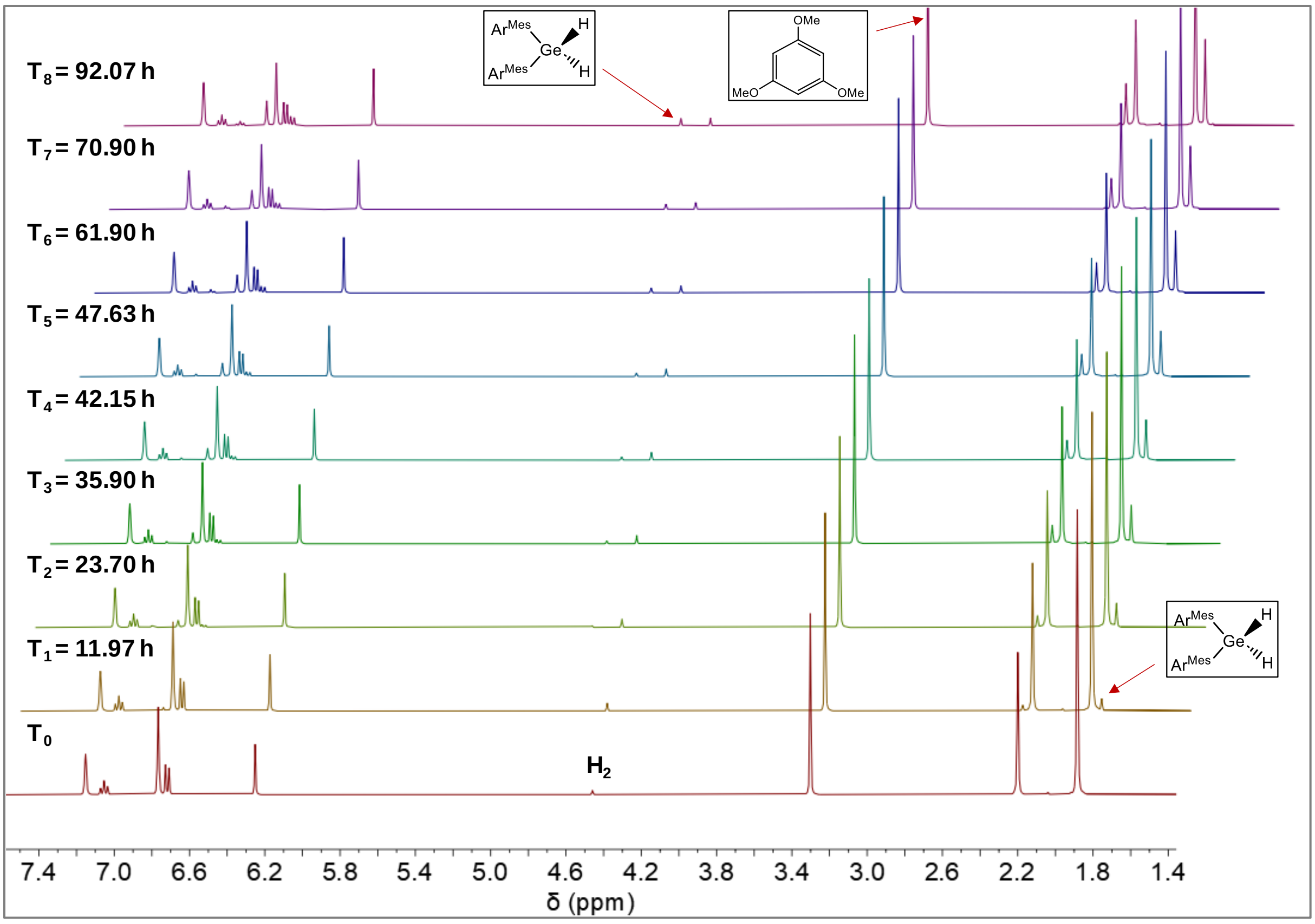

Figure S37. A stack plot of ^{1}H NMR spectra monitoring H_2 activation by germylene at 298 K.

Table S8. ^{1}H NMR-derived concentration profiles of the product and reactant at 298 K.

Time (hr)	Int. of P	[P]	[A]	ln[A]
0	0	0	0.026	-3.6497
11.97	1.09	1.19	0.02481	-3.6964
23.7	2.14	2.33	0.02367	-3.7437
35.9	3.13	3.41	0.02259	-3.7904
42.15	3.6	3.93	0.02207	-3.8133
47.63	3.94	4.3	0.0217	-3.8302
61.9	4.95	5.4	0.0206	-3.8823
70.9	5.63	6.14	0.01986	-3.919

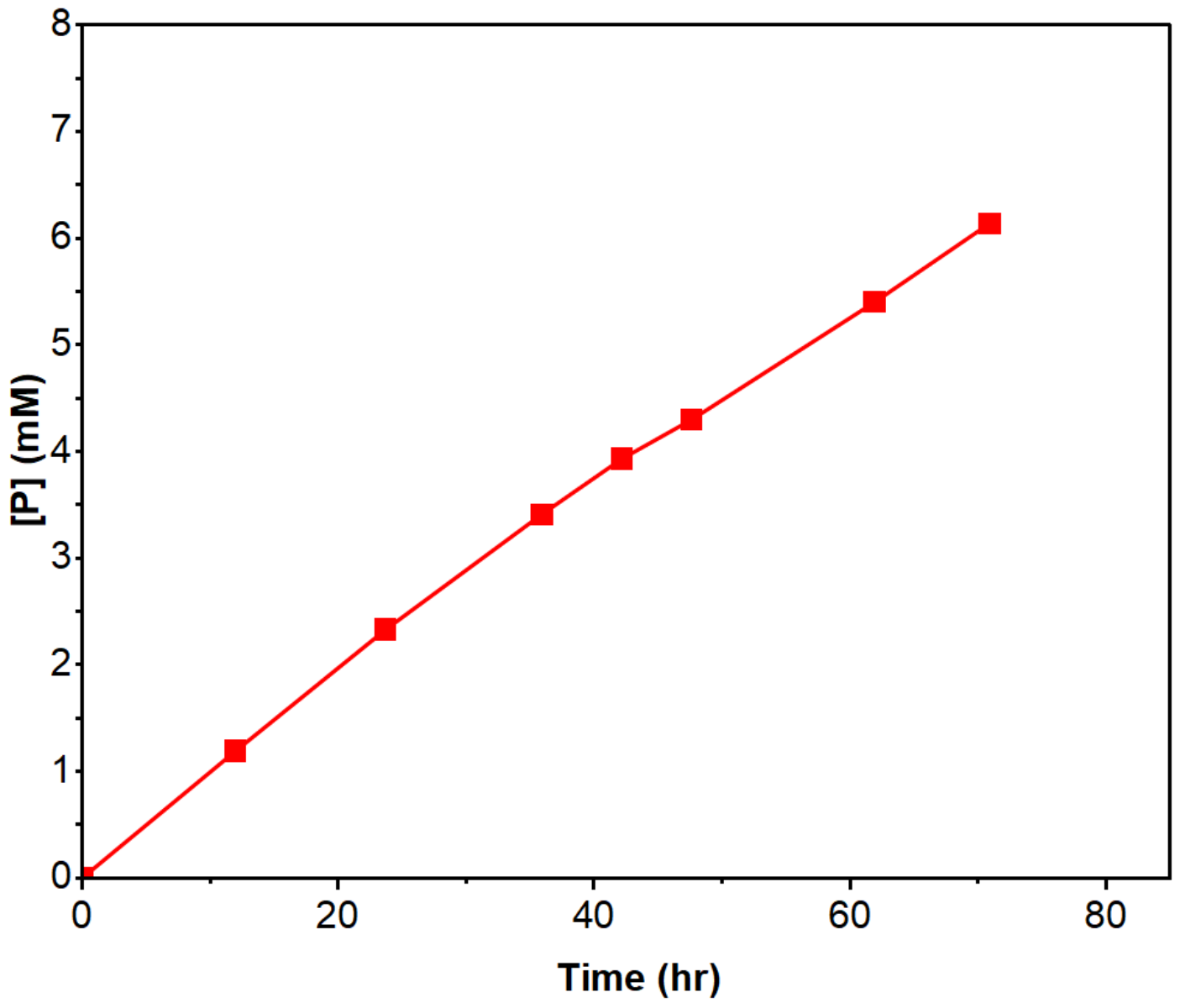

Figure S38. Plot of the conversion to the hydrogen-activated product over time at 298 K.

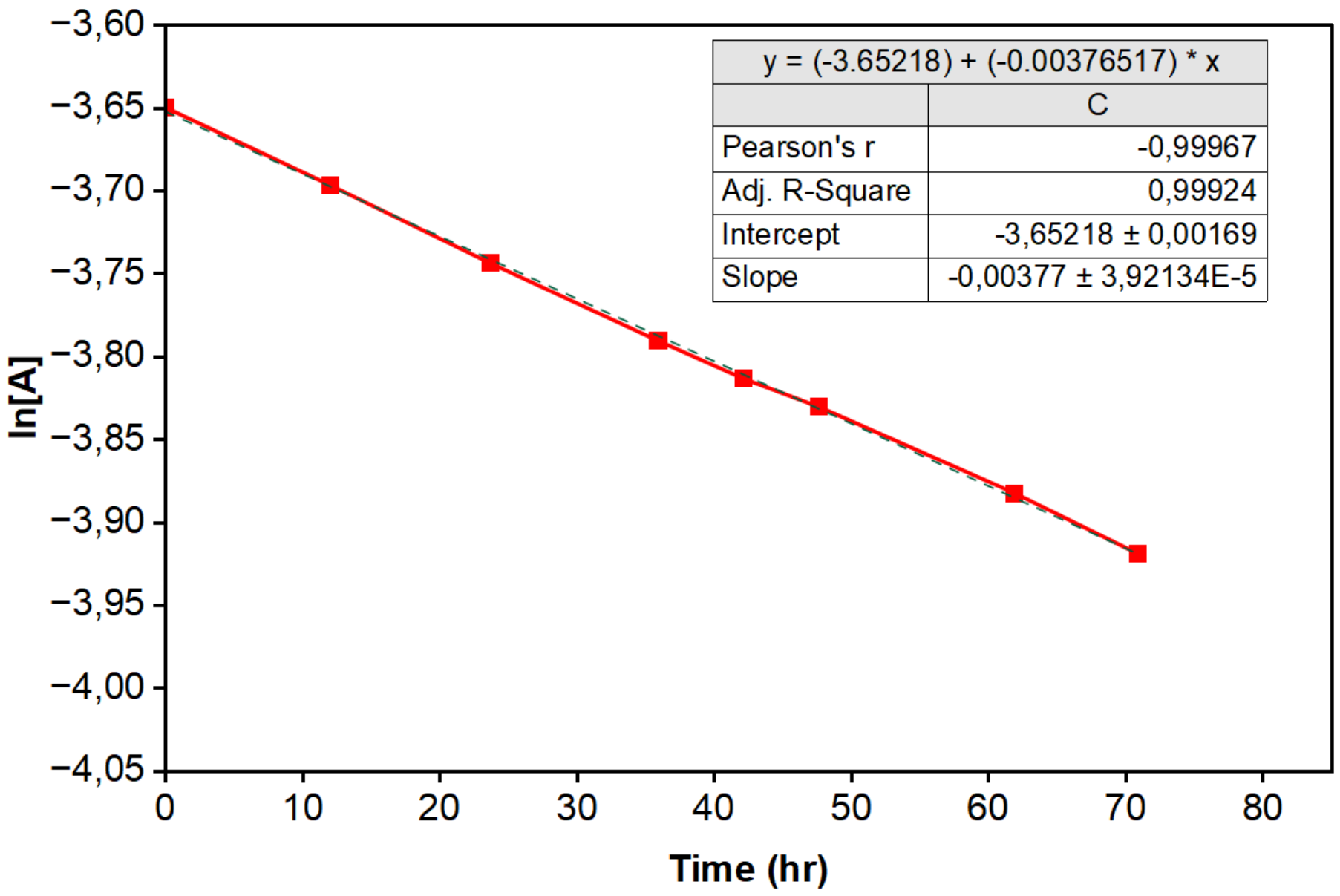

Figure S39. Plot of ln [A] vs time for H_2 activation at 298 K.

Eyring Plot

Table S9. Table of rate constants derived from the NMR experiments at different temperatures.

Temp.	1/T	Rate const. (k)	k/T	ln (k/T)
298 K	0.003356	1.047×10^{-6}	3.513×10^{-9}	-19.467
308 K	0.003247	2.614×10^{-6}	8.487×10^{-9}	-18.585
313 K	0.003195	4.222×10^{-6}	1.349×10^{-8}	-18.121

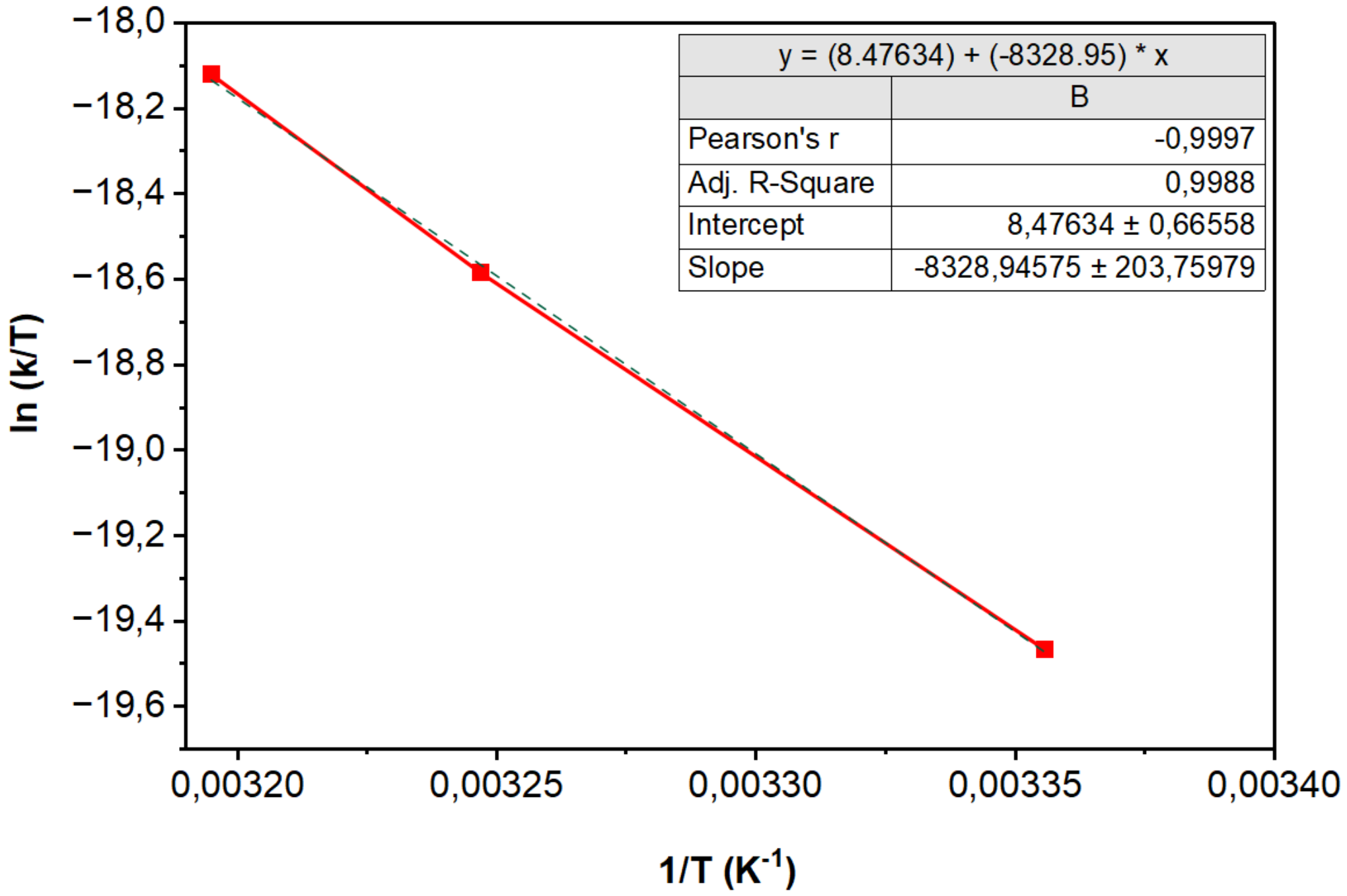

Figure S40. Eyring plot of **ln(k/T)** vs **1/T** derived from the experimentally determined rate constants.

$$\text{Ln}\,(k/T) = -(\Delta H^{\ddagger}/RT) + \ln(k_B/h) + (\Delta S^{\ddagger}/R)$$

$$\text{Slope} = -(\Delta H^{\ddagger}/R),\ \text{Intercept} = \ln(k_B/h) + (\Delta S^{\ddagger}/R)$$

Table S10. Activation parameters determined from the Eyring plot.

$\Delta H^{\ddagger}$.	$\Delta S^{\ddagger}$	$\Delta G^{\ddagger}$
69.3 ± 1,7 kJ mol^{-1}	–127.1 ± 5.5 J $mol^{-1}K^{-1}$	107.1 ± 2.4 kJ mol^{-1}

5 Crystal structure determination

5.1 General Information

Good quality single crystals of appropriate dimensions were placed in an inert oil such as perfluoropolyalkylether, hand-picked under polarized optical microscopy and then mounted on the diffractometer. The data collection was done at 100 K. X-ray intensity data measurements of all compounds were carried out on an Oxford SuperNova diffractometer with graphite-monochromatized (CuK_{α}= 1.54184 Å) radiation. The X-ray generator was operated at 50 kV and 30 mA. All structures were solved by intrinsic phasing and refined by the full-matrix least-squares on F^2 using software package and expanded using Fourier techniques.[26,27,28,29] Non-hydrogen atoms were refined anisotropically, while all hydrogen atoms were placed on the ideal positions using riding models and refined isotropically with displacement parameters constrained to those of the parent atoms (1.5 times Ueq for methyl groups and 1.2 times Ueq for all other carbon bound H). Data collection and structure refinement details for all compounds are given in the following tables. Further details on the structure refinement are provided in the following sections for each individual structure. Crystallographic data including structure factors have been deposited with the Cambridge Crystallographic Data Centre as supplementary publication no. CCDC - 2565935 and CCDC -2565936 Copies of the data can be gained free of charge on application to Cambridge Crystallographic Data Centre, 12 Union Road, Cambridge CB2 1EZ, UK; [fax: (+44) 1223-336-033; email: deposit@ccdc.cam.ac.uk].

5.2 Details on the structure solutions

Table S11. Data collection and structure refinement details for compounds **2** and **3**.

Structure	2	3
CCDC	2565935	2565936
Identification code	pap-076_auto_a_sq.cif	PAP-073_auto_a.cif
Empirical formula	C48 H59 Ge P	C89 H154 Ge2 P2 Si8
Formula weight	739.51	1655.95
Temperature	100(2) K	100(2) K
Wavelength	1.54184 Å (Cu Ka)	1.54184 Å (Cu Ka)
Crystal system	triclinic	triclinic
Space group	P -1 (no. 2)	P -1 (no. 2)
Unit cell dimensions	a = 10.89040(10) Å<br>b = 11.5039(2) Å<br>c = 19.9973(2) Å<br>a= 87.2020(10)°<br>b= 75.5370(10)°<br>g = 63.4110(10)°	a = 11.2558(3) Å<br>b = 13.1391(4) Å<br>c = 17.9047(4) Å<br>a= 78.716(2)°<br>b= 89.016(2)°<br>g = 69.531(2)°
Volume	2163.60(5) Å^3	2429.02(12) Å^3
Z	2	1
Density r(calculated)	1.135 g/cm^3	1.132 g/cm^3

Absorption coefficient μ	1.518 mm^{-1}	2.306 mm^{-1}
F(000)	788	894
Crystal size	0.420 x 0.260 x 0.220 mm^3	0.270 x 0.220 x 0.180 mm^3
Theta range for data collection	4.309 to 67.998°	2.520 to 67.999°
Index ranges	-11 <= h <= 13, -13 <= k< = 13, -23 <= l< = 23	-13 <= h <= 13, -15 <= k< = 15, -21 <= l< = 21
Reflections collected	27361	27787
Independent reflections	7856 [Rint = 0.0372, Rsigma = 0.0332]	8823 [Rint = 0.0396, Rsigma = 0.0365]
Completeness to theta = 67.684°	99.8 %	99.6 %
Absorption correction	gaussian	gaussian
Max. and min. transmission	1.000 and 0.337	1.000 and 0.361
Refinement method	full-matrix least-squares on F^2	full-matrix least-squares on F^2
Data / restraints / parameters	7856 / 0 / 465	8823 / 0 / 501
Goodness-of-fit on F2	1.044	1.041
Final R indices [I > 2 s(I)]	R1 = 0.0315, wR2 = 0.0824	R1 = 0.0311, wR2 = 0.0804
R indices (all data)	R1 = 0.0336, wR2 = 0.0836	R1 = 0.0348, wR2 = 0.0824
Largest diff. peak and hole	0.586 and -0.352 $e^{-}Å^{-3}$	0.616 and -0.342 $e^{-}Å^{-3}$

5.3 Crystal structure analysis of 2

All hydrogen atoms were placed on ideal positions. **2** was refined in ShelXle, and a highly disordered solvent molecule was removed *via* the SQUEEZE function of the platon software package.

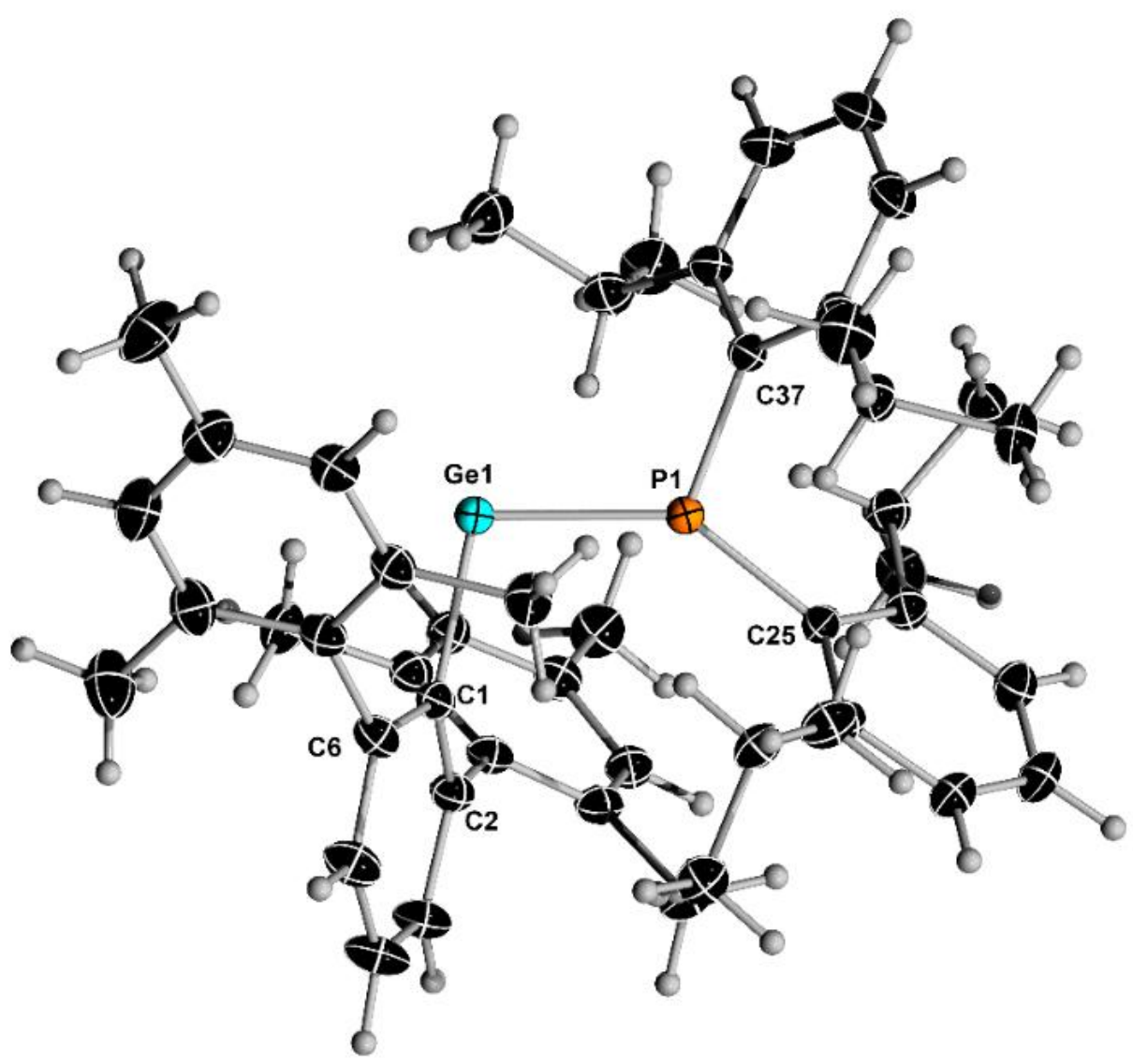

Figure S41. Molecular structure analysis of compound **2**. Thermal ellipsoids at 50% probability level. Selected bons lengths [Å] and angles [°]: Ge1-P1 2.232(1), Ge1-C1 2.002(2), P1-C37 1.839(2), P1-C25 1.848(2), C1-Ge1-P1 102.3(1), Ge1-P1-C37 111.1(1), Ge1-P1-C25 141.3(1), C37-P1-C25 107.6(1).

5.4 Crystal structure analysis of 3

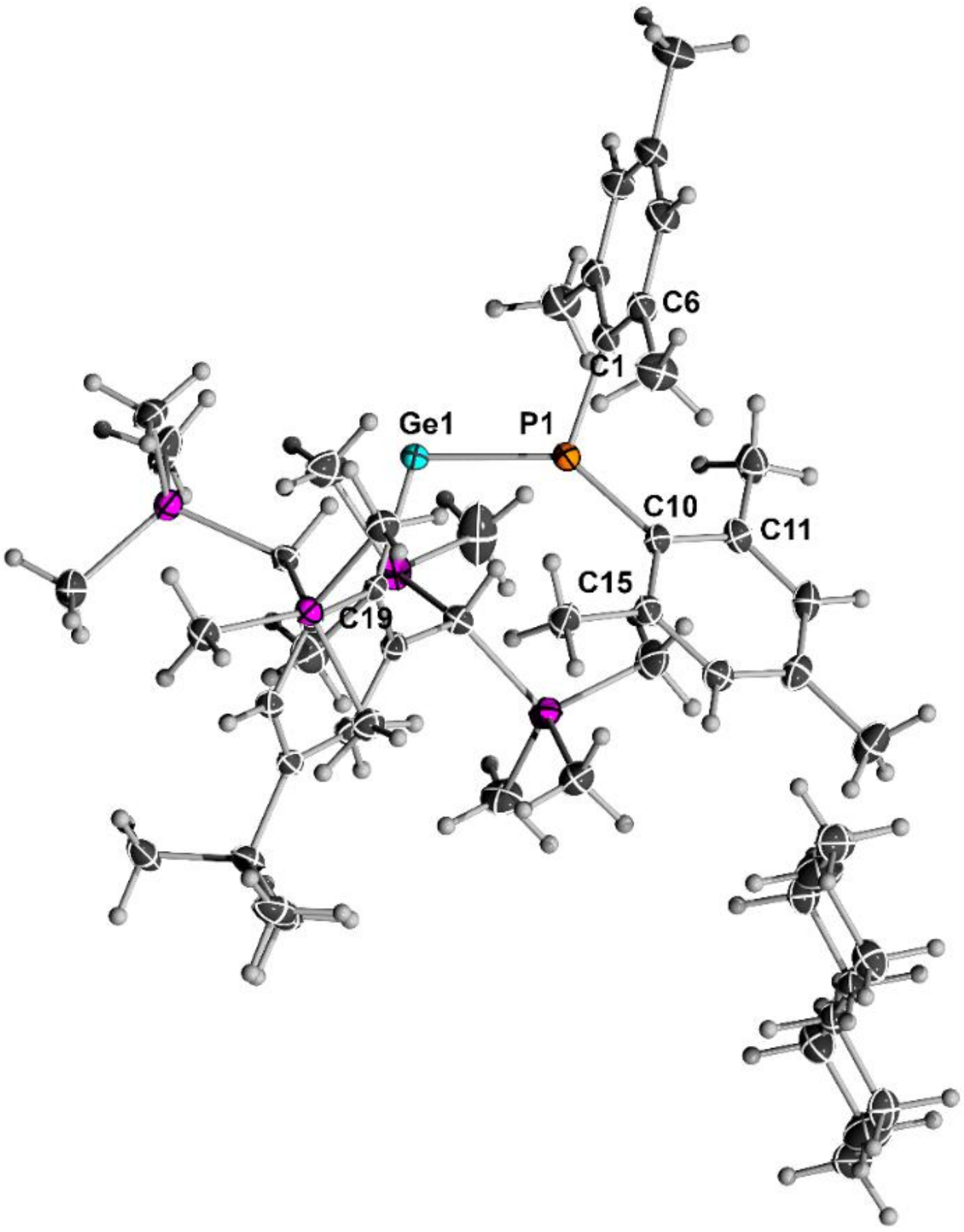

Figure S42. Molecular structure analysis of compound **3**. Thermal ellipsoids at 50% probability level. Selected bons lengths [Å] and angles [°]: Ge1-P1 2.225(1), Ge1-C19 2.009(2), P1-C1 1.825(2), P1-C10 1.827(2), C19-Ge1-P1 106.9(1), Ge1-P1-C1 109.3(1), Ge1-P1-C10 138.1(1), C1-P1-C10 112.2(1).

6 References

[1] Landrum, G. A. RDKit: Open-source cheminformatics, http://www.rdkit.org (**2020**).

[2] a) O'Boyle, N. M.; Banck, M.; James, C. A.; Morley, C.; Vandermeersch, T.; Hutchison, G.R. *J. Cheminf.* **2011**, *33*. b) The Open Babel Package http://openbabel.org (accessed December 2021).

[3] a) C. Bannwarth, S. Ehlert, S. Grimme, *J. Chem. Theory Comput.* **2019**, *15*, 1652–1671. b) C. Bannwarth, E. Caldeweyher, S. Ehlert, A. Hansen, P. Pracht, J. Seibert, S. Spicher, S. Grimme *WIREs Comput. Mol. Sci.* **2020**, *11*, e01493.

[4] a) S. Grimme, *J. Chem. Theory Comput.* **2019**, *155*, 2847–2862. b) P. Pracht, F. Bohle, S. Grimme, *Phys. Chem. Chem. Phys.*, **2020**, *22*, 7169–7192.

[5] Frisch, M. J.; Trucks, G. W.; Schlegel, H. B.; Scuseria, G. E.; Robb, M. A.; Cheeseman, J. R.; Scalmani, G.; Barone, V.; Petersson, G. A.; Nakatsuji, H.; Li, X.; Caricato, M.; Marenich, A. V.; Bloino, J.; Janesko, B. G.; Gomperts, R.; Mennucci, B.; Hratchian, H.P.; Ortiz, J. V.; Izmaylov, A. F.; Sonnenberg, J. L.; Williams-Young, D.; Ding, F.; Lipparini, F.; Egidi, F.; Goings, J.; Peng, B.; Petrone, A.; Henderson, T.; Ranasinghe, D.; Zakrzewski, V. G.; Gao, J.; Rega, N.; Zheng, G.; Liang, W.; Hada, M.; Ehara, M.; Toyota, K.; Fukuda, R.; Hasegawa, J.; Ishida, M.; Nakajima, T.; Honda, Y.; Kitao, O.; Nakai, H.; Vreven, T.; Throssell, K.; Montgomery, J. A., Jr.; Peralta, J. E.; Ogliaro, F.; Bearpark, M. J.; Heyd, J. J.; Brothers, E. N.; Kudin, K. N.; Staroverov, V. N.; Keith, T. A.; Kobayashi, R.; Normand, J.; Raghavachari, K.; Rendell, A. P.; Burant, J. C.; Iyengar, S. S.; Tomasi, J.; Cossi, M.; Millam, J. M.; Klene, M.; Adamo, C.; Cammi, R.; Ochterski, J. W.; Martin, R. L.; Morokuma, K.; Farkas, O.; Foresman, J. B.; Fox, D. J.Gaussian 16 Revision C.01.

[6] a) C. Adamo, V. Barone, *J. Chem. Phys.* **1999**, *110,* 6158–6169. b) M. Ernzerhof., G. E. Scuseria *The Journal of Chemical Physics* **1999**, *110*, 5029–5036.

[7] a) S. Grimme, J. Antony, S. Ehrlich, H. Krieg, *J. Chem. Phys.* **2010**, *132*, 154104. b) S. Grimme, S. Ehrlich, L. Goerigk, *J Comput Chem.* **2011**, *32*, 1456–1465.

[8] A. Schäfer, H. Horn, R. Ahlrichs, *J. Chem. Phys.* **1992**, *97*, 2571.

[9] a) F. Weigend, R. Ahlrichs, *Phys. Chem. Chem. Phys.* **2005**, *7*, 3297–3305. b) F. Weigend, Phys. Chem. Chem. Phys. **2006**, *8*, 1057–1065.

[10] J. Tomasi, B. Mennucci, R. Cammi, *Chem. Rev.* **2005**, *105*, 2999–3093.

[11] NBO 7.0. E. D. Glendening, J. K. Badenhoop, A. E. Reed, J. E. Carpenter, J. A. Bohmann, C. M. Morales, P. Karafiloglou, C. R. Landis, and F. Weinhold, Theoretical Chemistry Institute, University of Wisconsin, Madison, WI (2018).

[12] a) A. C. Hillier, J. W. Sommer, B. S. Yong, J. L. Petersen, L. Cavallo, S. P. Nolan, *Organometallics* **2003**, *22*, 4322–4326. b) L. Falivene, R. Credendino, A. Poater, A. Petta, L. Serra, R. Oliva, V. Scarano, L. Cavallo, *Organometallics* **2016**, *35*, 2286–2293. c) H. Clavier, S. P. Nolan, *Chem. Commun.* **2010**, *46*, 841–861.

[13] Jorner, K. *et al.*, MORFEUS (Python package), https://github.com/digital-chemistry-laboratory/morfeus

[14] A. Verloop, W. Hoogenstraaten, J. Tipker, *Drug Design* **1976**, *11*, 165–207.

[15] A. Shrake, J. A. Rupley, *J. Mol. Bio.* **1973**, *79*, 351–371.

[16] F. Eisenhaber, P. Lijnzaad, P. Argos, C. Sander, M. Scharf, *J. Comput. Chem.* **1995**, *16*, 273–284.

[17] a) R. L. Domingo, M. Ríos-Gutiérrez, P. Pérez, *Molecules*, **2016**, *21*, 6, 748. b) P. W. Ayers, J S. M. Anderson, J. I. Rodriguez, Z. Jawed, *Phys. Chem. Chem. Phys.*, **2005**, *7*, 1918–1925.

[18] a) T. Lu, F. Chen, *J. Comput. Chem.* **2012**, *33*, 580–592.

[19] C. A. Tolman, *J. Am. Chem. Soc.* **1970**, *92*, 2953−2956.

[20] T. Gensch, G. dos Passos Gomes, P. Friederich, E. Peters, T. Gaudin, R. Pollice, K. Jorner, A. Nigam, M. Lindner-D'Addario, M. S. Sigman, A. Aspuru-Guzik, *J. Am. Chem. Soc.* **2022**, *144*, 1205-1217.

[21] Rogers, D.; Hahn, *M. J. Chem. Inf. Model.* **2010**, *50*, 742–754.

[22] J.-Y. Guo, Y. Minko, C. B. Santiago, M. S. Sigman, *ACS Catal.* **2017**, *7*, 4144–4151.
[23] R. S. Simons, L. Pu, M. M. Olmstead, P. P. Power, *Organometallics* **1997**, *16*, 9, 1920–1925.
[24] T. Sasamori, T. Sugahara, T. Agou, J. D. Guo, S. Nagase, R. Streubel, N. Tokitoh, *Organometallics* **2015**, *34*, 11, 2106–2109.
[25] K. Izod, P. Evans, P. G. Waddell, *Dalton Trans.*, **2017**, *46*, 13824-13834.
[26] G. M. Sheldrick, “A short history of SHELX” *Acta Cryst A* **2008**, *64*, 112–122.
[27] G. M. Sheldrick, “Crystal structure refinement with SHELXL” *Acta Cryst C* **2015**, *71*, 3–8.
[28] G. M. Sheldrick, “SHELXT – Integrated space-group and crystal-structure determination” *Acta Cryst A* **2015**, *71*, 3–8.
[29] A. Thorn, B. Dittrich, G. M. Sheldrick, “Enhanced rigid-bond restraints” *Acta Cryst A* **2012**, *68*, 448–451.
